# Conserved molecular pathways underlying biting in two divergent mosquito genera

**DOI:** 10.1111/eva.13379

**Published:** 2022-04-26

**Authors:** Alden Siperstein, Sarah Marzec, Megan L. Fritz, Christina M. Holzapfel, William E. Bradshaw, Peter A. Armbruster, Megan E. Meuti

**Affiliations:** ^1^ Department of Entomology The Ohio State University Columbus Ohio USA; ^2^ Department of Biology Georgetown University Washington District of Columbia USA; ^3^ Department of Entomology University of Maryland College Park Maryland USA; ^4^ Laboratory of Evolutionary Genetics Institute of Ecology and Evolution University of Oregon Eugene Oregon USA

**Keywords:** blood feeding, *Culex pipiens*, life‐history evolution, mosquito‐borne disease, vector control, *Wyeomyia smithii*

## Abstract

Mosquitoes transmit a wide variety of devastating pathogens when they bite vertebrate hosts and feed on their blood. However, three entire mosquito genera and many individual species in other genera have evolved a nonbiting life history in which blood is not required to produce eggs. Our long‐term goal is to develop novel interventions that reduce or eliminate the biting behavior in vector mosquitoes. A previous study used biting and nonbiting populations of a nonvector mosquito, *Wyeomyia smithii*, as a model to uncover the transcriptional basis of the evolutionary transition from a biting to a nonbiting life history. Herein, we ask whether the molecular pathways that were differentially expressed due to differences in biting behavior in *W*. *smithii* are also differentially expressed between subspecies of *Culex pipiens* that are obligate biting (*Culex pipiens pipiens*) and facultatively nonbiting (*Culex pipiens molestus*). Results from RNAseq of adult heads show dramatic upregulation of transcripts in the ribosomal protein pathway in biting *C. pipiens*, recapitulating the results in *W. smithii*, and implicating the ancient and highly conserved ribosome as the intersection to understanding the evolutionary and physiological basis of blood feeding in mosquitoes. Biting *Culex* also strongly upregulate energy production pathways, including oxidative phosphorylation and the citric acid (TCA) cycle relative to nonbiters, a distinction that was not observed in *W*. *smithii*. Amino acid metabolism pathways were enriched for differentially expressed genes in biting versus nonbiting *Culex*. Relative to biters, nonbiting *Culex* upregulated sugar metabolism and transcripts contributing to reproductive allocation (vitellogenin and cathepsins). These results provide a foundation for developing strategies to determine the natural evolutionary transition between a biting and nonbiting life history in vector mosquitoes.

## 1 INTRODUCTION

Mosquitoes transmit a wide variety of vector‐borne diseases, including malaria, dengue, and filariasis (Roberts, 2002). Furthermore, the rapid emergence and global spread of additional mosquito‐borne viruses such as chikungunya and Zika are of increasing public health concern (Bradshaw et al., 2018; Fauci & Morens, 2016; Kilpatrick & Randolph, 2012). Effective vaccines and drug treatments are not available for the majority of mosquito‐borne pathogens. Consequently, efforts to reduce disease transmission have traditionally focused on reducing mosquito abundance, usually by reducing larval habitats (a.k.a. source reduction) or applying insecticides. However, the effectiveness of these traditional approaches is limited by the proliferation of man‐made habitats (e.g., discarded tires and cisterns) and the evolution of insecticide resistance. Hence, novel approaches to control the transmission of mosquito‐borne pathogens are desperately needed (McGraw & O'Neill, 2013).

Over the last two decades, a variety of new and promising strategies have been developed to either reduce mosquito abundance or inhibit pathogen transmission (Crawford et al., 2020; McGraw & O'Neill, 2013; Wang et al., 2021). However, all of these emerging approaches assume that a bite will occur. We are pursuing an alternative strategy to identify existing genetic variation in natural populations that enables a proportion of females to produce eggs without imbibing blood. Our long‐term goal is to develop genetic or chemical interventions that turn blood‐feeding mosquitoes into nonbiters based on the simple but powerful logic that if no bite occurs, transmission of blood‐borne pathogens is not possible (Armbruster, 2018).

The evolutionary transition from a biting to a nonbiting life history has occurred multiple times in mosquitoes. In fact, three complete genera of mosquitoes never bite (*Malaya*, *Topomyia*, *Toxorhynchites*), and several nonbiting species occur in genera comprised mostly of species that do bite (Downes, 1958; Foster, 1995; Miyagi et al., 2012; Rattanarithikul et al., 2007; Wahid et al., 2007; Zhou et al., 2014). Furthermore, many species are able to produce a single clutch of eggs without biting, but then require a blood meal for all subsequent egg clutches (O'Meara, 1985; Rioux et al., 1975; Spielman, 1971). The selective pressures driving the repeated, independent evolution of a nonbiting life history in mosquitoes are likely related to the costs of blood feeding, which are not widely appreciated. These costs include allocating energetic resources to locating vertebrate hosts, preparing to digest a blood meal (Bradshaw et al., 2018), surviving on a host (Edman & Scott, 1987), mitigating the thermal stress of imbibing a hot blood meal (Benoit et al., 2011), as well as detoxifying heme and iron as the blood is digested (Graca‐Souza et al., 2006). Bearing these myriad costs in mind, it is perhaps not surprising that northern, obligate nonbiting populations of the pitcher‐plant mosquito, *Wyeomyia smithii*, achieve higher lifetime reproductive success than southern populations of ancestrally biting mosquitoes, regardless of the presence or absence of a host (Borowczak, 2017; Bradshaw, 1980, 1986).

Bradshaw et al. (2018) used the pitcher‐plant mosquito, *W. smithii*, as a model system to determine the molecular physiology underlying the evolutionary transition from a biting to a nonbiting life history by artificially selecting a genetically variable population of *W*. *smithii* from Florida to generate two populations: avid biters and disinterested biters. The transcriptional response in the presence of a vertebrate host of these artificially selected populations were then compared to a naturally evolved, obligate nonbiting population from Maine. When all three populations were provided with the opportunity to blood‐feed, 1459 genes (<6% of the *W*. *smithii* genome) exhibited parallel differential gene expression between *both* the artificially selected avid biters and the unselected, disinterested biters from within the same Florida population *and* between avid biters from Florida and naturally evolved obligate nonbiters from Maine (Bradshaw et al., 2018). Results based on KEGG pathway analyses found that relative to nonbiting females, biting females of *W*. *smithii* transcriptionally upregulate several physiological processes with clear functional significance to blood feeding *before* blood is actually ingested. These artificially selected and naturally evolved populations of *W*. *smithii* that differed in biting behavior demonstrated an extraordinarily high level of genetic parallelism. We now seek to answer the question: Do the genes and pathways that distinguish blood‐feeding from obligate nonbiting populations of *W*. *smithii* (Bradshaw et al., 2018) predict pathways and genes that distinguish blood‐feeding from facultatively nonbiting individuals in two subspecies of the vector mosquito *Culex pipiens*?

To answer this question, we conducted similar experiments to those with *W*. *smithii* (Bradshaw et al., 2018), utilizing *C. pipiens* L., a primary vector of West Nile virus and filarial worms (Hamer et al., 2008; Lewandowski et al., 1980; Rajagopalan et al., 1977). The *C. pipiens* L. complex includes two interfertile subspecies, *Cx*. *p*. *pipiens* (hereafter, Pipiens) and *Cx*. *p*. *molestus* (hereafter, Molestus). The Pipiens and Molestus subspecies differ in a suite of ecophysiological traits, including above‐ versus below‐ground habitat utilization, mating behavior, host preference, and the ability to reproduce without biting (Haba & McBride, 2022; Noreuil & Fritz, 2021; O'Meara, 1985; Spielman, 1971; Strickman & Fonseca, 2012). Thus, similar to the comparison of *W*. *smithii* populations described above (Bradshaw et al., 2018), the challenge of our experimental design is that even when tissue samples are collected in the context of a behavioral biting assay, transcriptional differences between the Pipiens and Molestus subspecies could be due to differences unrelated to biting behavior. We address this challenge by specifically identifying transcriptional differences between biting Pipiens and nonbiting Molestus that were also associated with differences in biting behavior between populations of *W*. *smithii*. The simplest interpretation of these shared differences between biting and nonbiting mosquitoes from two genera separated by ~200 million years of evolutionary divergence (Reidenbach et al., 2009) is that the differences represent a conserved molecular physiological response to differences in biting behavior. Additionally, we focus on transcriptional differences between biting Pipiens and nonbiting Molestus that have a clear functional relevance to metabolism and reproductive physiology when producing eggs with or without ingesting blood.

Herein, we compare transcriptional differences in heads of biting Pipiens and nonbiting Molestus, utilizing previously characterized populations of these two subspecies with established differences in biting behavior and the capacity to reproduce without biting (Noreuil & Fritz, 2021). We identify KEGG pathways enriched for differentially expressed genes (DEGs) to elucidate the molecular physiology underlying the divergence between a biting versus nonbiting life history. We also determine transcriptional similarities and distinctions between biters versus nonbiters of *Cx*. *pipiens* with biters versus nonbiters of *W*. *smithii* predicted *a priori* from direct selection on biting in *W*. *smithii*. Our results present novel insights that provide a foundation for our long‐term goal of developing pharmacological or genetic strategies to reduce global transmission of pathogens by disease vectors.

## 2 MATERIALS AND METHODS

### 2.1 Insect colony maintenance

Colonies of Molestus and Pipiens utilized in this study correspond to BG1 (Molestus) and AG2 (Pipiens), respectively, of Noreuil and Fritz (2021). The Molestus population was established from the drainage sump in the Calumet Water Reclamation Plant in Chicago, Illinois (USA) in January of 2009 (Mutebi & Savage, 2009). The Pipiens population was collected from above‐ground field sites in Evanston, Illinois (USA) in August of 2016, and the 51st and 52nd lab generation were used in these experiments. Consistent with Noreuil and Fritz (2021), our preliminary experiments confirmed that >90% of Molestus females produced eggs without a blood meal within the first ~100 h of eclosion. Hence, most females from this population were disinterested in biting on the third day of adult life when tissue collections occurred (see below). Females from the Pipiens population required a bloodmeal to complete every gonotrophic cycle.

All mosquito life stages were reared in an environmental chamber at 26°C with ~70% relative humidity and a photoperiod consisting of 16 h of light and 8 h of dark (L:D 16:8). One‐hundred and fifty first instar larvae of Pipiens or Molestus were placed in pans with 600 ml of reverse osmosis (RO) water. Larvae were initially fed a slurry of beef liver powder (3 ml of 2.53% weight/volume solution) and subsequently fed 650 mg of ground Tetramin fish food over a period of 6 days. For both strains, one hundred pupae were placed into circular plastic containers (4.61 cm in diameter) containing ~200 ml of RO water, which previous experiments showed resulted in a high rate of emergence and low pupal mortality. The pupae in these containers were then placed inside 12″ × 12″ × 12″ mesh cages (BioQuip) provisioned with 10% sucrose solution, organic raisins, and honey‐soaked sponges, which served as food sources for male and female mosquitoes. To ensure that the adults in each cage were the same age, cups containing pupae were moved every 24 h to new cages each day at Circadian Time (CT) 8.5 (i.e., 8.5 h after lights had turned on).

### 2.2 Head tissue collections

Nonbiting females of Molestus and biting females of Pipiens were collected using methods that were as close as possible to a previous study of transcriptional changes associated with differences in biting behavior in the pitcher‐plant mosquito, *W*. *smithii* (Bradshaw et al., 2018). All females were collected three‐days postadult emergence during an approximately 1‐hour period between CT12 and CT15, corresponding to between 4 and 1 h(s), respectively, before the lights turned off. This narrow collection window was used to minimize any differences in gene expression that might arise due to the timing of collections. All food sources were removed on Day 2 (CT12; 24 h before collections) to encourage females to bite. Both the Molestus and Pipiens were reared in the same incubator at the same time, and whenever possible, samples of both strains were collected on the same day.

Nonbiting females of Molestus were collected by discarding any females that attempted to bite the human blood source during the one‐hour trial period (0–5 females/cage; <1.7% of total females attempted to bite). After the one‐hour period, 35 of the remaining, non‐biting Molestus were removed from the cage, snap frozen in ethanol on dry ice, and decapitated. These 35 collected heads were pooled into a single biological replicate sample for RNAseq analysis. An additional sample containing 10 heads was collected for qRT‐PCR analyses. One biological replicate sample for RNAseq and one biological replicate sample for qRT‐PCR were collected from a single cage containing 3‐day‐old female mosquitoes each day over a four‐day period (*n* = 3 replicate samples for RNAseq; *n* = 5 replicate samples for qRT‐PCR; see Table S1).

Biting female Pipiens were collected by aspirating any females that landed on the human blood source, probed their mouthparts into the source and inserted their proboscis until the labium was bent and the fascicle was exposed. Importantly, these biting females were collected *before* they had to the opportunity to imbibe any blood. Females were snap frozen and decapitated. Two biological replicate samples containing 35 heads and 1 biological replicate sample containing 31 heads of 3‐day‐old biting Pipiens were collected over 3 days for RNAseq experiments (see Table S1). Any additional females who bit were also collected for subsequent qRT‐PCR analyses (9–15 heads/sample; *n* = 5 samples; Table S1).

### 2.3 RNA extraction, library preparation, and sequencing

All samples for RNAseq analysis were homogenized in 500 µl of TRIzol (Invitrogen) and shipped to the University of Oregon Genomics and Cell Characterization Core Facility (GC3F) where RNA extraction, cDNA library preparation, and sequencing were performed. Briefly, head tissue of each sample was disrupted by beating with Silica grinding beads in a Spex Genogrinder 2100 (2 × 1500 RPM for 2 min). RNA was extracted using a Zymo Direct‐Zol kit (Zymo Research) and mRNA was isolated using Oligo dT beads according to manufacturer's instructions. Integrity assessment was performed on an RNA chip (Bioanalyzer 2100) and all samples that were sequenced had a RNA Quality Number between 6.7–10.0. Six RNA samples (three biting, three nonbiting) were utilized for paired‐end, barcoded, and stranded library construction with the Universal Plus mRNA‐Seq protocol (Tecan Genomics). All six libraries were combined in equimolar ratios and 150‐bp reads were sequenced on single lane of an Illumina HiSeq 4000 instrument. The raw reads are available in NCBI’s sequence read archive (SRA) under accession number [dataset] PRJNA787258 (Siperstein et al., 2022).

### 2.4 Bioinformatics analyses

Details of the bioinformatics workflow can be found on the GitHub repository here: [dataset] https://github.com/srmarzec/Culex_Biting_RNAseq/blob/main/MasterNotes.md (Marzec, 2021). Briefly, reads were cleaned with Trimmomatic (version 0.39) using default settings with the exception of a flag for HEADCROP:15 to remove the first 15 bases from each read. Reads were then mapped using a two‐pass method in STAR (version 2.7.1a) to the most recent available *Culex quinquefasciatus* JHB strain reference genome sequence (GCF_015732765.1). Read counts were obtained from the resulting bam files with HTSeq (version 0.13.5). We included genes that had at least 10 reads across all six samples. To determine if biological replicate samples within treatments (biting, nonbiting) exhibited similar overall transcriptional profiles, read counts were transformed to a log2 scale using rlog and then a principle components analysis (PCA) was performed using plotPCA in DESeq2 (version 1.30.1). Next, differential expression analysis was performed using DESeq2 on normalized read counts in an R environment (version 4.0.2). Differentially expressed genes (DEGs) were identified as genes with a Benjamini–Hochberg false discovery rate adjusted *p*‐value less than 0.05 and a log_2_ fold change >|1|.

Because the most recent *Cx*. *quinquefasciatus* genome sequence (GCF_015732765.1) does not have available KEGG pathway annotations, locus tags (CpipJ_CPIJ IDs) were retrieved from the previous *Cx*. *quinquefasciatus* genome sequence (GCA_000209185.1) using the NCBI efetch utility (Entrez Direct E‐utility). Locus tags corresponding to GCF_015732765.1 gene IDs were used to identify KEGG pathways that were enriched for DEGs. All *Cx*. *quinquefasciatus* KEGG pathways and genes within those pathways were downloaded from KEGG using KEGGREST (version 1.30.1) in R. A custom script was used to identify the number of DEGs relative to the total number of genes in each pathway. Significant enrichment was tested using a Wilcoxon Rank Sum test. Only KEGG pathways that had 5 or more DEGs and a *p*‐value less than 0.05 were considered to be enriched for DEGs.

Finally, common DEGs between biting versus nonbiting mosquitoes were identified for *Cx*. *pipiens* and *W*. *smithii*. To do so, for each differentially expressed gene between biting Pipiens and nonbiting Molestus that was present in the significantly enriched KEGG pathways, putative orthologues between *W*. *smithii* and *Cx*. *pipiens* were identified based on matching CpipJ_CPIJ IDs (locus tags). The *Cx*. *pipiens* CpipJ_CPIJ IDs were obtained as described above for KEGG pathway analysis. For *W*. *smithii* transcripts, the *orthologous Cx*. *pipiens* transcript (CpipJ_CPIJ ID) was obtained as previously described in Bradshaw et al. (2018). As some *W*. *smithii* transcripts were assigned matching CpipJ_CPIJ IDs and thus are duplicated, there are some duplicate occurrences of *Cx*. *pipiens* transcripts as orthologs from our work.

### 2.5 Confirming differential gene expression using qPCR

Quantitative real‐time PCR (qPCR) of independent tissue samples was used to confirm the differential gene expression results from RNAseq analysis. Heads of 3‐day‐old, nonbiting Molestus and 3‐day‐old, biting Pipiens (*n* = 9–15 heads/biological replicate; 5 biological replicates/subspecies; Table S1) were collected as described above and RNA was isolated from the samples using TRIzol according to the manufacturer's protocol, using 1/2 of the reaction volumes. The amount and quality of the RNA were measured using a Nanodrop spectrophotometer. cDNAs for each sample were synthesized using 0.1 mg of total RNA and the Maxima First Strand cDNA Synthesis Kit (Thermo Fisher) according to the manufacturer's instructions. Primers for six genes that were upregulated in nonbiting Molestus and five genes that were upregulated in biting Pipiens were designed using Primer3 (Untergasser et al., 2012; Table S2). Prior to qPCR analyses, melt and standard curves were run to ensure that each primer set met MIQE specificity and efficiency guidelines (Bustin et al., 2009; Table S2). qRT‐PCR was performed in a 96‐well plate using an CFX Connect qPCR detection system (Bio‐Rad). All reactions were performed in triplicate in a total volume of 10 μl containing 5 μl iTaq Universal SYBR green PCR Master Mix (Bio‐Rad), 400 nmol of each primer, and 1 μl sample cDNA.

The qPCR data were analyzed by first averaging the relative cycle threshold (CT) of three technical replicates. The resulting CT value for each gene of interest within each biological replicate was normalized to the geometric average of the CT values of three reference genes (*Rp49*, *RpL19*, and *28S*) by subtracting the average CT of reference genes from the CT value for the gene of interest (2^−ΔCT^ method). A Student's *t*‐test was then used to compare the average relative expression of a gene of interest between the five biological replicates in biting Pipiens and nonbiting Molestus samples (α = 0.05).

## 3 RESULTS

**FIGURE 1:**
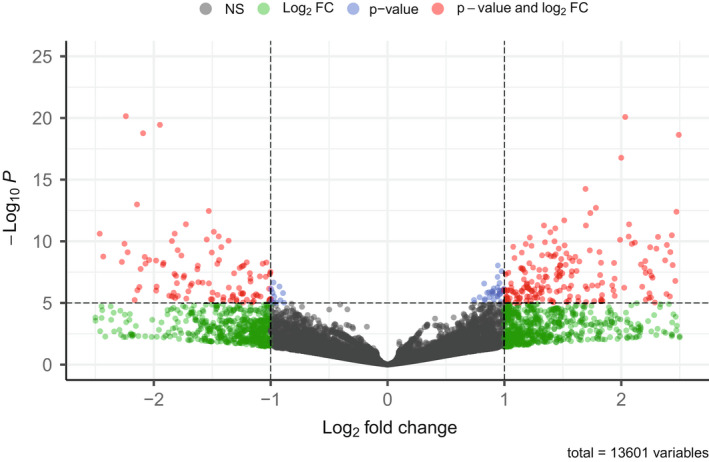
Differential gene expression of 13,601 genes in Pipiens and Molestus. Each point represents differential expression for a single gene. Gray points indicate no significant differences, green points show log_2_ fold change values that are not statistically significant, blue points show statistical significance but low log_2_ fold change values, and red points indicate genes that show both statistical significance and an absolute fold change value greater than 2

RNA sequencing of three biological replicate samples of nonbiting Molestus head tissue and three biological replicate samples of biting Pipiens head tissue produced a total of 369,193,543 raw read pairs (range = 66,888,763–53,998,743 read pairs per sample). Of these, between 86.3% and 78.5% of read pairs per replicate sample remained after filtering with Trimmomatic. The STAR two‐pass alignment produced alignment rates between 72.5% and 62.0% per sample. Finally, HTseq identified between 19,932,197 and 31,239,605 reads pairs per sample that aligned to gene models (Table S3). 13,601 gene models had at least ten mapped read pairs across the six samples, corresponding to 90% of the 15,094 annotated protein coding genes in the *Culex quinquefasciatus* reference genome sequence (GCF_015732765.1). Principal component analysis showed that transcriptional profiles of biting versus nonbiting samples were clustered within treatments and strongly separated on the first principal component axis, which explained 75% of the variance in gene expression (Figure S1). Biting samples were also strongly clustered on the second principal component axis (15% of variance), while one nonbiting sample was distinct from the other two replicate samples (Figure S1). Overall, a total of 1444 genes were significantly differentially expressed (*p *< 0.05, log2FC > 1) between nonbiting Molestus and biting Pipiens samples (Figure 1, Table S4). A list of the top 15 upregulated and downregulated genes in Pipiens relative to Molestus is presented in Table 1.

**TABLE 1:** Top 30 differentially expressed genes by log_2_ fold change (FC) for Pipiens relative to Molestus

Gene ID	Gene name	Log2 fold change	*p*‐Value
LOC6047742	General odorant‐binding protein 72	15.7024718	2.02E‐25
LOC6043552	Microfibril‐associated glycoprotein 4	13.7040153	8.70E‐20
LOC119767898	CLIP domain‐containing serine protease 14D‐like	13.2928199	6.41E‐19
LOC119769981	Cuticle protein 16.5‐like	13.1376982	3.13E‐18
LOC6045845	Phenoloxidase‐activating factor 2	12.8489124	3.08E‐17
LOC6033001	Cuticle protein	12.3405023	4.84E‐15
LOC119767852	Cuticle protein 16.5‐like	10.0954717	2.54E‐14
LOC6032993	Larval cuticle protein A3A	11.7787979	2.57E‐13
LOC6046699	Cuticle protein 8	11.2499226	5.67E‐12
LOC119769968	Cuticle protein 12.5‐like	10.8373473	2.23E‐09
LOC119769830	Cuticle protein 21‐like	10.1740576	9.06E‐09
LOC119770586	Cuticle protein 16.5‐like	10.1029209	1.51E‐08
LOC6049801	Flexible cuticle protein 12	9.99068121	1.64E‐08
LOC119769857	Cuticle protein 38‐like	9.96120093	3.78E‐08
LOC6049115	CD209 antigen	9.92152709	1.08E‐07
LOC6039374	Phenoloxidase 2	−14.366537	3.18E‐21
LOC119766533	Cathepsin B‐like	−9.5326752	1.89E‐18
LOC119770564	Cathepsin B‐like	−11.985519	1.77E‐17
LOC6043264	Probable cytochrome P450 9f2	−12.352691	2.75E‐16
LOC6043252	Vitellogenin‐A1	−9.6525665	1.42E‐11
LOC6030831	Esterase B1	−10.795463	2.91E‐11
LOC6040913	Dynein light chain 1, axonemal	−8.990121	1.21E‐10
LOC6051506	Probable cytochrome P450 6a13	−10.326134	9.73E‐10
LOC6043250	Vitellogenin‐A1	−9.8891799	2.96E‐09
LOC6031554	Cathepsin B	−9.7127498	3.45E‐08
LOC6031556	Cathepsin B	−8.9974469	4.34E‐07
LOC6044517	Polyserase‐2	−9.1632547	1.25E‐06
LOC6031746	Trans‐1,2‐dihydrobenzene‐1,2‐diol dehydrogenase	−9.0679375	1.98E‐06
LOC6038440	EF‐hand calcium‐binding domain‐containing protein 1	−8.9958486	2.00E‐06
LOC6035285	Lamin Dm0	−9.1267936	2.52E‐05

Note

*p*‐Values are adjusted for multiple comparisons using a Benjamini–Hochberg false discovery rate correction.

KEGG pathway enrichment analysis resulted in 17 pathways significantly enriched for DEGs (Table 2). Below, we focus on the specific results for KEGG pathways and related individual genes of particular biological interest. We begin by discussing pathways upregulated in Pipiens relative to Molestus. For example, pathways related to transcription, translation, and energy metabolism were upregulated in biting relative to nonbiting mosquitoes. Concerning translation, the KEGG pathway for “Ribosome” contained 123 annotated *Cx*. *quinquefasciatus* genes, of which 85 were differentially expressed; all of these 85 DEGs were upregulated in Pipiens relative to Molestus. Additionally, the ribosome pathway contained 18 inferred orthologs between *Cx*. *pipiens* and *W*. *smithii*, all of which were upregulated in avid‐ versus reluctant‐biting *W*. *smithii*. Furthermore, the transcript for SUMO, which encodes a protein involved in post‐translational modification, was also upregulated in both biting Pipiens and biting *W*. *smithii* relative to their nonbiting counterparts (Table 1 and Table S4). Relevant to transcription, the “RNA polymerase” KEGG pathway contained 27 annotated *Cx*. *pipiens* genes. Seven genes were DEG, of which six were significantly upregulated in biting relative to nonbiting mosquitoes. The two KEGG pathways related to energy metabolism that were significantly enriched for DEGs were “Citrate cycle (TCA cycle)” and “Oxidative phosphorylation.” In the oxidative phosphorylation pathway, the 80 annotated genes included 50 DEGs, all of which were upregulated in biting Pipiens. The citrate cycle included 27 annotated genes, of which nine were DEG and seven were upregulated in biting Pipiens.

**TABLE 2:** KEGG pathways that were significantly enriched (Total DEGs >5 and *p*‐value < 0.05) for differentially expressed genes of biting Pipiens or nonbiting Molestus mosquitoes

Pathway code	Pathway name	Annotated culex genes in pathway	Total DEGs	Up‐regulated biting DEGS	Up‐regulated nonbiting DEGs	*p*‐Value
cqu03010	Ribosome	123	85	85	0	2.22E‐35
cqu00190	Oxidative phosphorylation	80	50	50	0	5.94E‐18
cqu00520	Amino sugar and nucleotide sugar metabolism	44	15	3	12	0.002
cqu03013	RNA transport	118	28	11	17	0.007
cqu00380	Tryptophan metabolism	21	9	7	2	0.009
cqu04215	Apoptosis—multiple species	21	7	4	3	0.011
cqu04214	Apoptosis—fly	49	12	5	7	0.019
cqu00981	Insect hormone biosynthesis	30	8	4	4	0.019
cqu00310	Lysine degradation	28	8	6	2	0.020
cqu00010	Glycolysis/Gluconeogenesis	35	8	5	3	0.025
cqu00350	Tyrosine metabolism	20	8	2	6	0.026
cqu00620	Pyruvate metabolism	31	11	8	3	0.030
cqu00513	Various types of N‐glycan biosynthesis	30	10	4	6	0.030
cqu00051	Fructose and mannose metabolism	28	6	1	5	0.031
cqu00280	Valine, leucine, and isoleucine degradation	35	8	7	1	0.033
cqu00020	Citrate cycle (TCA cycle)	27	9	7	2	0.047
cqu03020	RNA polymerase	26	7	6	1	0.048

Two KEGG pathways involved in amino acid degradation were upregulated in biting Pipiens. The “Valine, Leucine, and Isoleucine degradation” pathway included 35 annotated *Culex* genes, of which eight were DEG with seven upregulated in biters. The “Lysine degradation” KEGG pathway included 28 annotated *Culex* genes, of which eight were DEG, with six upregulated in biters.

Compared to the results above describing KEGG pathways upregulated in biting Pipiens relative to nonbiting Molestus, fewer KEGG pathways were enriched for upregulated DEGs in non‐biting mosquitoes. The “Fructose and mannose metabolism” KEGG pathway contained 28 annotated *Culex* genes, and six of these were differentially expressed. Five of these six DEGs were upregulated in nonbiting Molestus relative to biting Pipiens. The “Tyrosine metabolism” KEGG pathway was also upregulated in nonbiters relative to biters, with 20 annotated genes, and 8 of these were DEG with 6 upregulated in nonbiters.

A previous study in *W*. *smithii* (Bradshaw et al., 2018) identified 1459 transcripts that were consistently differentially expressed in two comparisons between biting and nonbiting mosquitoes of this species. As noted above, all of these *W*. *smithii* transcripts were assigned putative *Culex* orthologues (CpipJ_CPIJ IDs) as described in Bradshaw et al. (2018). In the current study, we identified 1444 transcripts that were differentially expressed between biting Pipiens and nonbiting Molestus and 1089 of these could be assigned CpipJ_CPIJ IDs using the efetch utility in Entrez Direct E‐utility (see above). Of these 1459 *W*. *smithii* transcripts and 1089 *Cx*. *pipiens* transcripts that were differentially expressed between biters and nonbiters, 172 transcripts in *W*. *smithii* are homologous to 156 transcripts in *Cx*. *pipiens*. Of the transcripts that were unique to *Cx*. *pipiens*, 84 were upregulated in biting Pipiens and avid‐biting *W*. *smithii* relative to their nonbiting and reluctant‐biting counterparts. In contrast, only 11 transcripts were upregulated in nonbiting Molestus and reluctant‐biting *W*. *smithii* relative to biters (Table S5).

qRT‐PCR results support the results of the RNAseq analysis by finding that DEGs identified by RNAseq are differentially expressed in independent samples of biting and nonbiting *Cx*. *pipiens*. Specifically, qRT‐PCR results demonstrate that *angiopoietin*‐*related protein 6* (LOC6052987), *probable cytochrome P450 6a14* (LOC6037495), and *ficolin*‐*3* (LOC6046433) were upregulated in Pipiens head‐tissue samples, similar to the RNAseq results (Table S6). The remaining 3 genes that RNAseq analyses indicate were upregulated in biting Pipiens relative to non‐biting Molestus (*hexamerin 1*.*1*, *larval cuticle protein A3A* and *cuticle protein 38*) were not found to be differentially expressed in our qRT‐PCR analyses (Table S6). However, *vitellogenin*‐*A1* (LOC6043252), *fumarylacetoacetase* (LOC6052229), *esteraseB1* (LOC6030831), *cathepsin B* (LOC6049222), and *L*‐*galactose dehydrogenase* (LOC6037771) were overexpressed in Molestus when measured with both RNAseq (Table 1) and qRT‐PCR (Figure S2), representing all five Molestus‐upregulated genes that we selected for qRT‐PCR analysis. Moreover, the differences in fold change were similar in our qRT‐PCR analyses and in our RNAseq results (Table S6).

## 4 DISCUSSION

The evolutionary transition from a biting to nonbiting life history has occurred multiple times in mosquitoes, including three entire genera of mosquitoes that never bite (*Malaya*, *Topomyia*, *Toxorhynchites*), and several nonbiting species that occur in genera comprised mostly of species that do bite (Downes, 1958; Foster, 1995; Miyagi et al., 2012; Rattanarithikul et al., 2007; Rioux et al., 1975; Spielman, 1971; Wahid et al., 2007; Zhou et al., 2014). Additionally, several mosquito species make a transition from a biting to a nonbiting life history when they enter adult, reproductive diapause in response to short days (reviewed in Denlinger & Armbruster, 2014, 2016). Differences in gene expression between nondiapausing (biting) and diapausing (nonbiting) *Cx*. *pipiens pipiens* have previously been measured using suppression subtractive hybridization (Robich & Denlinger, 2005; Robich et al., 2007). Our long‐term goal is to identify common molecular and physiological differences between biting and nonbiting mosquitoes so that we may develop novel strategies to prevent biting in vector species. A previous study used *W*. *smithii* as a model system to determine the molecular underpinnings of the evolutionary transition from a biting to a nonbiting life history between populations within a single species (Bradshaw et al., 2018). Herein, we extend the range of evolutionary divergence from that study to determine whether molecular pathways involved in the evolutionary divergence of a blood‐feeding versus nonblood‐feeding life history in *W*. *smithii* are also differentially regulated between two previously characterized subspecies of *C. pipiens* that are obligate blood feeding (Pipiens) and facultatively nonbiting (Molestus; Noreuil & Fritz, 2021). This comparison establishes the unprecedented opportunity to identify conserved transcriptional responses related to biting versus nonbiting across a broad evolutionary timescale between different genera of mosquitoes estimated to have diverged in nature ~200 Mya (Reidenbach et al., 2009).

We first briefly review the transcriptional changes associated with the transition from an ancestral blood‐feeding life history to an evolutionarily derived nonblood‐feeding life history in *W*. *smithii*. We then discuss our current experimental results on transcriptional differences in head tissues between populations of biting Pipiens and nonbiting Molestus. We focus on pathways and gene clusters relevant to unifying concepts such as anticipatory costs and metabolic flexibility, in preference to discussing distinctions on a gene‐by‐gene basis. Throughout, we highlight similarities and distinctions between biting and nonbiting *Culex* and *Wyeomyia*. Finally, we discuss directions for future research.

### 4.1 Transcriptional differences between biting versus nonbiting *Wyeomyia smithii*

As described above (see Section 1), the previous study of Bradshaw et al. (2018) utilized a comparison of *both* naturally evolved *and* artificially selected populations of *W*. *smithii* to identify transcriptional differences that contribute to the evolution of a nonbiting life history. Bradshaw et al. (2018) concluded that the evolution of a nonbiting life history resulted in reduced anticipatory costs of biting and increased opportunistic metabolic flexibility. The reduced anticipatory costs include reduced prebiting investment in proteasomal, spliceosomal, ribosomal, and odorant receptor proteins. The opportunistic metabolic flexibility includes increased expression of enzymes that produce metabolic intermediates in the pyruvate metabolic pathway (Acetyl‐CoA) and the purine metabolic pathway (Inosine monophosphate), providing nonbiters with the opportunity to exploit diverse downstream metabolic pathways in response to varied environmental conditions. As we show below, differential gene expression between obligate biting Pipiens and nonbiting Molestus both overlap with and differ from *W*. *smithii*.

### 4.2 Anticipatory upregulation of translational machinery in biting Pipiens

**FIGURE 2:**
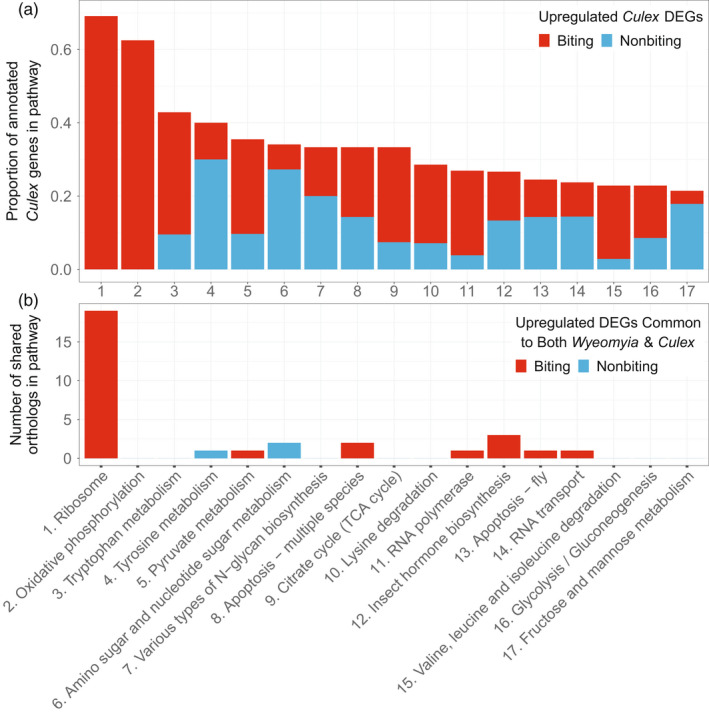
Differentially expressed genes in each significantly enriched KEGG pathway. (a) Proportion of total annotated Culex genes that are differentially expressed genes (upregulated) in either biting Pipiens (red) or nonbiting Molestus (blue). (b) Number of significantly differentially expressed orthologs in each KEGG pathway for either biting (Pipiens and *Wyeomyia smithii*—avid biting; red) or nonbiting (Molestus and *W*. *smithii*—disinterested; blue). Numerical labels on the *x*‐axis of each panel correspond to KEGG pathway labels in Table 2

Biting Pipiens exhibit dramatic upregulation of the translational machinery, starting with three of the six genes encoding components of RNA polymerase I, which specifically transcribes ribosomal RNAs and two components of RNA polymerase III, which, in turn, transcribes transfer RNAs (Khatter et al., 2017). Furthermore, the KEGG pathway associated with ribosomes is highly enriched for DEGs, with 69% of genes encoding ribosomal proteins upregulated in biting female mosquitoes (Table 2, Figure 2a). Specific genes upregulated in Pipiens relative to Molestus include two eukaryotic translation initiation factors (*eIF5A*, LOC6032328 and *eIF6*, LOC6045183) and the post‐translational protein modifier SUMO (*small*, *ubiquitin*‐*related modifier 3*; LOC604408; Table S4). The upregulation of SUMO is particularly interesting as this protein can post‐translationally modify hundreds of different proteins (Hannoun et al., 2010; Hay, 2005) and is involved in a diverse range of pathways (Mauri et al., 2008), including suppressing arboviruses (Stokes et al., 2020).

Upregulation of transcripts encoding ribosomal proteins and SUMO in biting Pipiens closely parallels the transcriptional response of blood‐feeding *Wyeomyia*, with remarkable overlap in the two genera in both the large and small ribosomal subunits (Figure S3). These results represent a clear affirmative answer to our original question: Are homologous genes associated with blood‐feeding in the same functional pathway similarly differentially expressed between selected lines within a population of *W*. *smithii*, between populations of *W*. *smithii*, and between genera of mosquitoes (*Wyeomyia* vs. *Culex*)? Because this upregulation of the translational machinery and SUMO occurs before blood is actually imbibed, and because translation is energetically costly (Kafri et al., 2016; Lynch & Marinov, 2015), we conclude that this response represents an anticipatory cost of blood feeding in blood‐feeding Pipiens and *W*. *smithii* (Bradshaw et al., 2018).

### 4.3 Differential energy production and reproductive allocation in biters versus nonbiters

**FIGURE 3:**
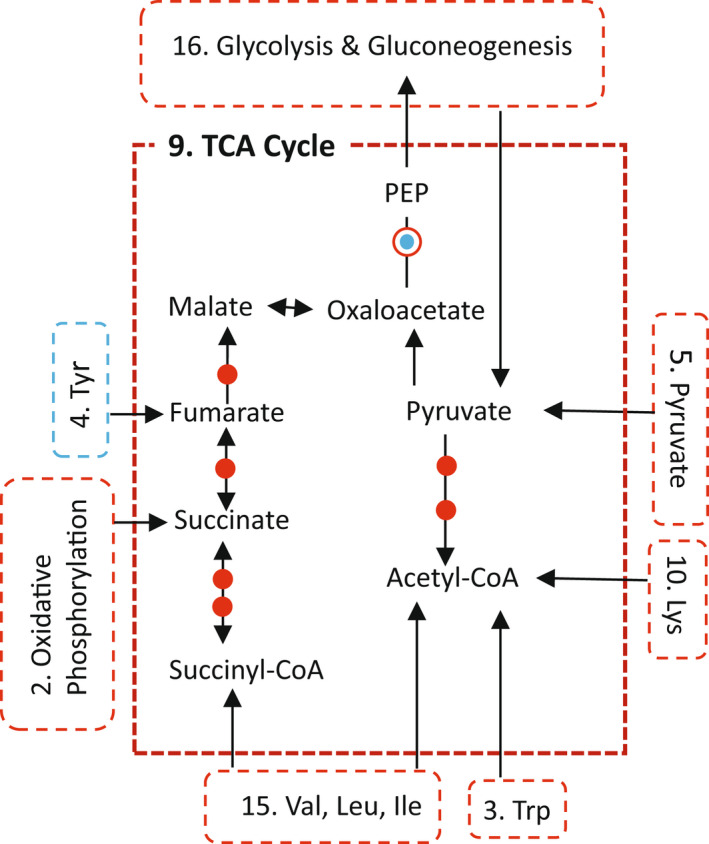
Citric Acid (TCA) cycle. Dashed outlines, significantly enriched KEGG pathways in *Cx*. *pipiens*: red, upregulated in Pipiens; blue, upregulated in Molestus; arrows indicate tracks from/to/within pathways. Pathway numeric labels correspond to those in Table 2 and Figure 2. Red dots and circle, upregulated in Pipiens; blue dot, upregulated in Molestus

The energetically demanding investment in translation by biting Pipiens coincides with a strong upregulation of energy production pathways. Both the oxidative phosphorylation and citric acid (TCA) KEGG pathways are strongly enriched for DEGs that are upregulated in Pipiens relative to Molestus (Table 2). Upregulation of the citric acid (TCA) cycle in Pipiens relative to Molestus includes not only many TCA enzymes, but involvement of the by‐ or end‐products of at least six other KEGG pathways enriched for DEGs that are primarily upregulated in Pipiens versus Molestus (Figure 3, Table 2). These inputs include contributions from pathways involved in glycolysis and gluconeogenesis, pyruvate metabolism, metabolism of five amino acid metabolic pathways, and oxidative phosphorylation, including proteins involved in the terminal electron transfer of all five complexes (Figure S4). Additionally, biting Pipiens strongly upregulate *hexamerin* 1.1 (LOC6041441), a key storage protein (Table S4). This finding is consistent with a previous study comparing gene expression in whole bodies of Pipiens and Molestus (Kang et al., 2021). Thus, biting Pipiens exhibit an anticipatory and coordinated response in which they initiate translation and upregulate enzymes involved in energy production pathways *before* the blood meal is actually consumed.

Nonbiting Molestus invest in sugar metabolism and reproductive allocation pathways that represent a commitment to ovary maturation without blood. First, the fructose and mannose metabolism KEGG pathway is enriched for DEGs, with five of six transcripts upregulated in Molestus relative to Pipiens (Table 2). Adult sugar metabolism supports the synthesis of glycogen and triacylglycerols; the latter are the primary constituent of lipid droplets in the ooplasm (Clements, 1992, p. 363). Additionally, the upregulation of four genes in nonbiting Molestus imply that these females have initiated the deposition of yolk protein into their eggs or vitellogenesis; these genes include two isoforms of *vitellogenin*‐*A1* [LOC6043252; LOC6043250] as well as two *Cathepsin B* transcripts (LOC6031544, LOC6031556) and two Ca*thepsin B*‐*like protein* transcripts (LOC119766533, LOC119770564; Table 1). While Cathepsin‐family proteases can have diverse physiological functions (Mort & Buttle, 1997), Cathepsin B proteins are known to be involved in degrading vitellogenin during embryogenesis in *Ae*. *aegypti* (Cho et al., 1999). Additionally, Moura et al. (2015) demonstrate that two *cathepsin B* transcripts are highly expressed in vitellogenic females of *Cx*. *quinquefasciatus*, and their associated proteins are subsequently active within the ovaries of females. Moreover, Kang et al. (2021) found that *Cathepsin C* (CIPJ000566) is also upregulated in whole bodies of nonbiting Molestus relative to biting Pipiens. Taken together, these results illustrate that nonbiting Molestus constitutively produce the transcripts necessary to provision their embryos with energy. In nonbiting *W*. *smithii*, c*athepsin B* mRNAs were also upregulated in reluctant‐biting relative to biting females (Bradshaw et al., 2018), highlighting c*athepsin B* as a critical gene involved in the evolution of a nonbiting life history across mosquito genera.

### 4.4 Metabolism of excess amino acids from acquired or stored resources

Two different amino acid metabolism pathways offset contrasting excesses of specific amino acids in biting Pipiens and nonbiting Molestus. These differences reflect a metabolic response to acquired resources in adult Pipiens (i.e., blood) versus stored larval resources in Molestus (i.e., hexamerin storage proteins). Interestingly, neither of these pathways was detected in the comparison of biting versus nonbiting *W*. *smithii*.

In biting Pipiens, the principal environmental source of protein is hemoglobin acquired in vertebrate blood. In particular, valine and leucine comprise over 20% of the amino acids in hemoglobin and Lysine another 7.6% (UniProtKB, 2021). Upregulating the valine–leucine–isoleucine and lysine metabolic pathway not only results in end products that can enter the TCA cycle (Figure 3) but also serves to deplete an upstream excess of valine, leucine, and lysine that are released when hemoglobin is catabolized.

**FIGURE 4:**
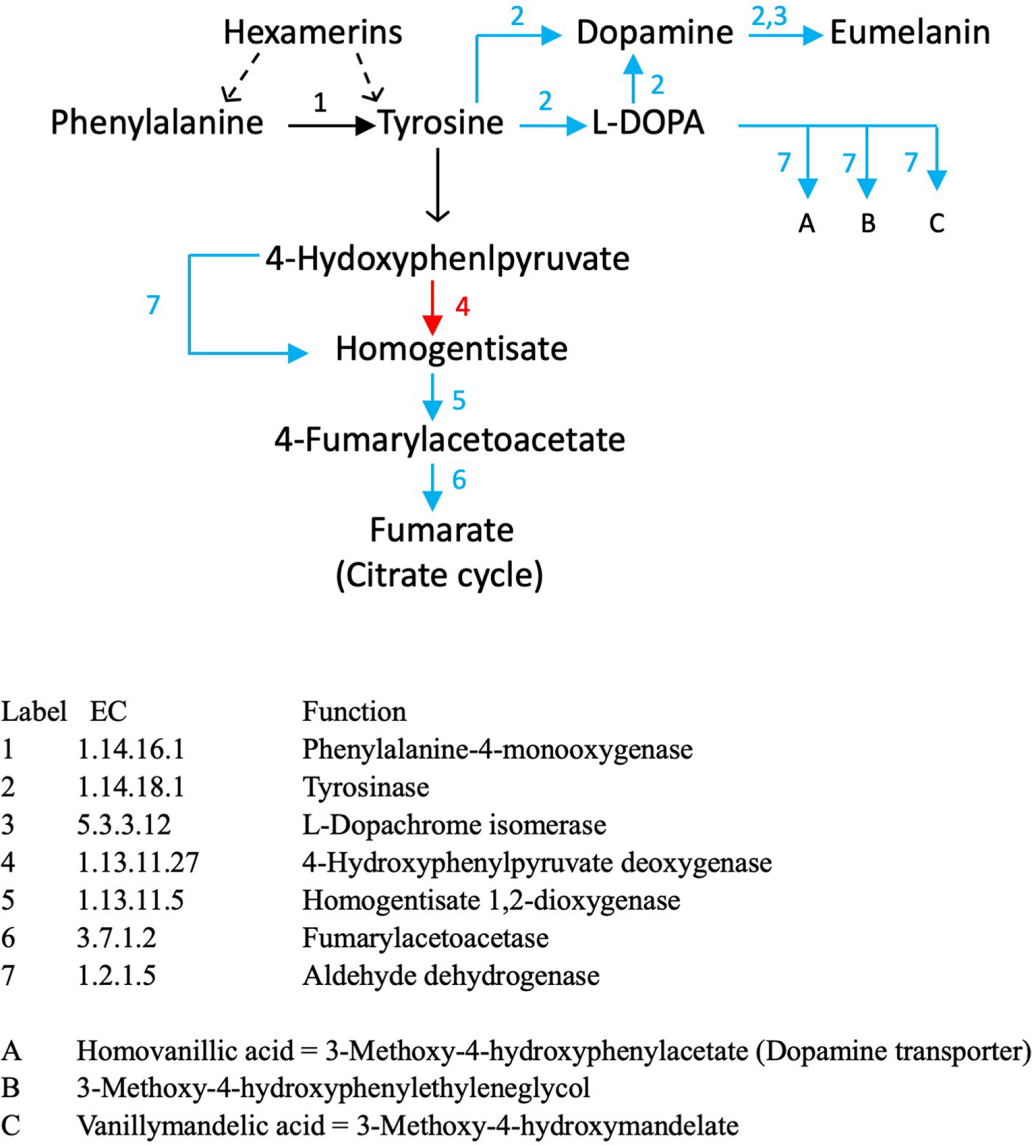
Tyrosine metabolism in *Culex pipiens*. Relevant enzymes are indicated by arrows: Red, upregulated in Pipiens; Blue, upregulated in Molestus; Black solid, non‐DEG steps; Black dashed, inferred. Specific enzymes are indicated by numbers or letters associated with arrows

In nonbiting Molestus, reproduction must be fueled from proteins accumulated and stored as larvae. The main storage proteins in the fat body and hemolymph of larvae are hexamerins (Beintema et al., 1994; Burmester, 1999), which in addition to their role as a source of amino acids during metamorphosis, have been implicated as a source of amino acids that supports vitellogenesis in nonblood feeding mosquitoes (Wheeler & Buck, 1996; Zakharkin et al., 2001). Hexamerins are particularly rich in tyrosine and phenylalanine (Burmester, 1999; Crampton et al., 1999; Korochkina et al., 1997, Table 1; UniProtKB, 2021), the latter being catalyzed to the former by *phenyalanine*‐*4*‐*hydroxylase* (Figure 4; Li & Christensen, 1993). This enzyme is not a DEG; nonetheless, depletion of tyrosine should enhance the phenylalanine to tyrosine reaction through mass action. Upregulation of tyrosine metabolism in Molestus (Table 2, Figure 4) is then consistent with metabolism of stored hexamarins and compensatory degradation of excess of tyrosine and phenylalanine (Figure 4).

In addition to the benefit of metabolizing excess tyrosine and phenylalanine, Tyrosine metabolism in Molestus also generates fumarate that enters directly into the TCA cycle and generates dopamine that acts as a substrate for eumelanin synthesis. Generation of both products is consistent with ongoing embryogenesis: *Fumarylacetoacetase* (Figure 4) enhances late‐stage embryogenesis and successful hatching in *Rhodnius prolixus* (Sterkel & Oliveira, 2017). A transcriptional commitment to melanization in nonbiting Molestus is also indicated by upregulation of *serine protease Hayan* (Table S4), which is responsible for eventual hardening of egg chorions (Dudzic et al., 2019; Li, 1994). Additionally, *chitin synthase* (*ch*‐*2*) and *endochitinase* (Table S4) are upregulated in Molestus and are important for recycling old to new chitin (Hamid et al., 2013; Muthukrishnan et al., 2019). In sum, tyrosine metabolism in nonbiting Molestus females supports energy production, embryonic viability, and the eventual development and hardening of egg chorions, as well as serving to maintain amino acid balance generated by metabolizing stored larval protein (Figure 4).

## 5 CONCLUSIONS AND FUTURE DIRECTIONS

Anticipatory upregulation of transcripts in the ribosomal protein pathway in biting mosquitoes relative to their nonbiting counterparts exhibits remarkably strong overlap in both the mosquito genera *Wyeomyia* and *Culex*. All 18 upregulated ribosomal proteins in biting *W*. *smithii* overlapped with the upregulated ribosomal proteins in biting *Cx*. *pipiens* (Figure 2), making the ancient and highly conserved ribosome the intersection to understanding the evolutionary and physiological basis of the anticipation of blood‐feeding in mosquitoes. In blood‐feeding Pipiens, this anticipatory commitment to the energetically costly process of translation coincides with increased energy production by oxidative phosphorylation, the TCA cycle, and multiple diverse pathways feeding into the TCA cycle. In contrast, sugar metabolism is upregulated in nonbiting Molestus, as well as constitutively provisioning the maturing ovaries with energy in the absence of a blood meal by upregulating *vitellogenin* and *cathepsin B* transcripts. Finally, contrasting patterns of amino acid metabolism in biting Pipiens and nonbiting Molestus are both mechanisms that maintain amino acid homeostasis in response to the utilization of acquired resources (blood for Pipiens) or stored resources (hexamerins for Molestus).

In sum, blood‐feeding in both *W*. *smithii* and *Cx*. *pipiens* was associated with anticipation of acquired resources and ribosomal protein synthesis was strikingly upregulated in blood‐feeders of both species. Nonbiting in *W*. *smithii* was associated with an opportunistic life history, characterized by sensory input and by metabolic pathways ending at “gateway” branch points; nonbiting in *Cx*. *pipiens* was associated with alternative pathways including metabolizing sugar and stored larval resources to support ongoing ovarian maturation. Uncovering a conserved, highly overlapping transcriptional response in biters of both species, and more diverse, nonoverlapping transcriptional responses in nonbiters, reflects biting as the ancestral character state in the Culicidae and the independent evolution of nonbiting in *W*. *smithii* and *Cx*. *pipiens molestus* (Grimaldi & Engel, 2005; Mans, 2011). An immediate next step will be to confirm the broad generality of anticipatory upregulation of the ribosome pathway by performing similar experiments in additional vector mosquito species. The commonality of this highly conserved response may provide a novel opportunity to interrupt or inhibit pathways necessary for the transmission of blood‐borne disease due to biting. Because the biting rate has a large impact on disease transmission as estimated by vectorial capacity, even relatively modest decreases in the biting rates of vector species are expected to have a large impact on reducing disease transmission (Black & Moore, 2005). An additional goal will be to perform mechanistic studies to determine whether enhanced sugar metabolism and constitutively provisioning maturing ovaries with both lipids and yolk proteins in nonbiting mosquitoes is necessary for reproduction without a blood meal. Ultimately, understanding the costs of biting and the molecular pathways underlying the evolution of a nonbiting life history will provide a foundation to develop pharmacological or genetic strategies to recapitulate this evolutionary transition, which has already occurred multiple times in nature.

## ACKNOWLEDGEMENTS

The views and opinions presented here are solely those of the authors and do not necessarily represent those of their employers or funding sources. The authors thank members of the Armbruster and Meuti labs for their thoughtful critiques on earlier drafts of this manuscript. This research was supported by a grant from the National Institutes of Health awarded to Peter Armbruster (R21‐AI144266) and a grant from the National Science Foundation awarded to Megan Meuti (IOS‐1944324).

## CONFLICT OF INTEREST

The authors declare no conflict of interest.

## Supporting information

Supplementary Material

## Data Availability

Data for this manuscript are available at *to be completed after manuscript is accepted for publication*. The raw reads are available in NCBI’s sequence read archive (SRA) under accession number PRJNA787258.
