# Supplementary material for: Conserved molecular pathways underlying biting in two divergent mosquito genera
Source: Evol Appl. 2022 Apr 26;15(5):878–90. doi: 10.1111/eva.13379 (PMC9108309; doi:10.1111/eva.13379)

Supplemental Information for:

Conserved Molecular Pathways Underlying Biting in Two Divergent Mosquito Genera

Siperstein, A., Marzec, S., Fritz, M.L., Holzapfel, C.M., Bradshaw, W.E., Armbruster, P.A., Meuti, M.E.

E-mail: meuti.1@osu.edu

**This PDF file includes:**

Tables S1 to S6

Figures S1 to S4

**Other supplementary materials for this manuscript include the following:**

Datasets S1 to S2

Table S1. Samples collected for RNAseq and qPCR. *All mosquitoes were 3 days old at the time of exposure to a human blood source, and were exposed and collected from a single cage.*

| **Sample name** | **Biting status** | **Collection Date** | **Exposure time** | **Number of female heads** | **Used for RNAseq** |
| --- | --- | --- | --- | --- | --- |
| Molestus.RNAseq.1 | Non-biting | 27-Aug-20 | CT12-13 | 35 | Yes |
| Molestus.RNAseq.2 | Non-biting | 28-Aug-20 | CT12-13 | 35 | Yes |
| Molestus.RNAseq.3 | Non-biting | 28-Aug-20 | CT13-14 | 35 | No |
| Molestus.RNAseq.4 | Non-biting | 29-Aug-20 | CT12-13 | 35 | Yes |
| Molestus.RNAseq.5 | Non-biting | 30-Aug-20 | CT12-13 | 35 | Yes |
| Pipiens.RNAseq.1 | Biting | 28-Aug-20 | CT14-15 | 35 | Yes |
| Pipiens.RNAseq.2 | Biting | 29-Aug-20 | CT14-15 | 35 | Yes |
| Pipiens.RNAseq.3 | Biting | 30-Aug-20 | CT13-14 | 31 | Yes |
| Pipiens.RNAseq.4 | Biting | 30 & 31 Aug-20 | CT14-15 (8/30); CT13-14 (8/31) | 33 | No |
| Pipiens.RNAseq.5 | Biting | 31 Aug-20 | CT12-13 | 35 | No |
| Pipiens.RNAseq.6 | Biting | 29-Aug-20 | CT13-14 | 35 | No |
| Molestus.qPCR.B1 | Biting | 28-Aug-20 | CT12-13 | 1 | NA |
| Molestus.qPCR.B2 | Biting | 28-Aug-20 | CT13-14 | 3 | NA |
| Molestus.qPCR.B3 | Biting | 29-Aug-20 | CT12-13 | 5 | NA |
| Molestus.qPCR.B4 | Biting | 30-Aug-20 | CT12-13 | 1 | NA |
| Molestus.qPCR.N1 | Non-biting | 27-Aug-20 | CT12-13 | 10 | NA |
| Molestus.qPCR.N2 | Non-biting | 28-Aug-20 | CT12-13 | 10 | NA |
| Molestus.qPCR.N3 | Non-biting | 28-Aug-20 | CT13-14 | 10 | NA |
| Molestus.qPCR.N4 | Non-biting | 29-Aug-20 | CT12-13 | 10 | NA |
| Molestus.qPCR.N5 | Non-biting | 30-Aug-20 | CT12-13 | 10 | NA |
| Pipiens.qPCR.B1 | Biting | 28-Aug-20 | CT14-15 | 10 | NA |
| Pipiens.qPCR.B2 | Biting | 29-Aug-20 | CT14-15 | 9 | NA |
| Pipiens.qPCR.B3 | Biting | 29-Aug-20 | CT13-14 | 10 | NA |
| Pipiens.qPCR.B4 | Biting | 29-Aug-20 | CT13-14 | 15 | NA |
| Pipiens.qPCR.B5 | Biting | 31-Aug-2020 & 1-Sept-2021 | CT12-13 | 10 | NA |

Table S2. Primers used for qRT-PCR.

| **Primer Name** | **Sequence** | **Melt temp (°C)** | **Gene ID** | **Primer efficiency** | **R^2^** |
| --- | --- | --- | --- | --- | --- |
| Cp.angio6.qFw1 | ctggaaccggtgcatcagat | 60.11 | LOC6052987 | 96.4% | 0.992 |
| Cp.angio6.qRv1 | gtccacttctccagcaagct | 59.96 |  |  |  |
| Cp.capBl.qFw2 | cggaccagggatgttgtgat | 59.75 | LOC6049222 | 92.6% | 0.996 |
| Cp.capBl.qRv2 | catcgaacgcactgaacacc | 59.84 |  |  |  |
| Cp.cp38.qFw2 | cgctgcctatccctatgctt | 59.68 | LOC6031357 | 96.0% | 0.991 |
| Cp.cp38.qRv2 | catagggatagtacggggaagc | 59.5 |  |  |  |
| Cp.cP450.qFw1 | ttcggaattgcacagctcct | 59.96 | LOC6037495 | 104.0% | 0.988 |
| Cp.cP450.qRv1 | gtccaaccacaatccaccct | 59.89 |  |  |  |
| Cp.eB1.qFw1 | gccacctttctgatcgacct | 59.75 | LOC6030831 | 113.7% | 0.990 |
| Cp.eB1.qRv1 | acggaatcccctgaaagctc | 59.75 |  |  |  |
| Cp.faa.qFw2 | cgaacttagctggaagggca | 60.04 | LOC6052229 | 93.8% | 0.988 |
| Cp.faa.qRv2 | ctcatcgttccggcaaaagc | 60.18 |  |  |  |
| Cp.fic3.qFw1 | aacttggcttacatgcggga | 59.96 | LOC6046433 | 91.3% | 0.999 |
| Cp.fic3.qRv1 | aaaccaccagcctcccaaaa | 60.03 |  |  |  |
| Cp.hex1.qFw1 | CCGGCCATCTACGAAATCTA | 60.05 | LOC6041441 | 114.0% | 0.984 |
| Cp.hex1.qRev1 | TTGCTAGCGAAACCGAACTT | 60.02 |  |  |  |
| Cp.lcA3A.qFw1 | acccgaaccctcagtactctt | 60.2 | LOC6033003 | 96.4% | 0.983 |
| Cp.lcA3A.qRv1 | agtacgatccctggacgaca | 60.03 |  |  |  |
| Cp.Lgal.dhyd.qFw2 | gggacgttacgagcaggatt | 60.04 | LOC6037771 | 82.7% | 0.982 |
| Cp.Lgal.dhyd.qRv2 | cagataacccacaccgagca | 60.11 |  |  |  |
| Cp.vit.qFw1 | CAACTGCAAGATTGCCAGAA | 59.99 | LOC6043252 | 98.3% | 0.977 |
| Cp.vit.qRev1 | AGTCCAGTTACGGGCATACG | 60.02 |  |  |  |

Table S3. Summary of reads pairs at sequential steps of processing for differential expression analysis. Sample labels correspond to Table S1.

Table S4. Differently expressed genes (DEGs) between non-biting Molestus and biting Pipiens (1,444 genes total). Log2Foldchange refers to biting Pipiens relative to non-biting Molestus.

| **GeneID** | **Gene annotation** | **log2FoldChange** | **Adjusted P-value** |
| --- | --- | --- | --- |
| LOC6042152 | uncharacterized LOC6042152 | 6.961564384 | 2.70E-80 |
| LOC6053994 | uncharacterized LOC6053994 | 9.189298596 | 2.70E-80 |
| LOC6048581 | bifunctional protein STORR | 6.481613528 | 2.29E-72 |
| LOC6052832 | uncharacterized LOC6052832 | 8.606171668 | 1.65E-70 |
| LOC6051412 | glycine-rich cell wall structural protein 1.8 | 5.289368596 | 1.82E-58 |
| LOC6050480 | uncharacterized LOC6050480 | 5.910835391 | 2.59E-58 |
| LOC119771227 | 40S ribosomal protein S20-like | 6.892264481 | 5.96E-58 |
| LOC6033116 | uncharacterized LOC6033116 | 6.016871927 | 5.83E-53 |
| LOC6051295 | A-kinase anchor protein 14 | 6.450664355 | 2.44E-51 |
| LOC6042539 | uncharacterized LOC6042539 | 6.291999988 | 1.98E-50 |
| LOC119770052 | uncharacterized LOC119770052 | 7.437059535 | 1.01E-47 |
| LOC6041589 | proline-rich protein 4 | 7.959661057 | 5.51E-40 |
| LOC6041441 | hexamerin-1.1 | 6.626887559 | 1.44E-39 |
| LOC6052987 | angiopoietin-related protein 6 | 6.98335822 | 1.16E-35 |
| LOC119768758 | uncharacterized LOC119768758 | 4.544796555 | 3.87E-35 |
| LOC6035976 | serine protease Hayan | -5.934624522 | 2.01E-31 |
| LOC119770010 | probable serine/threonine-protein kinase kinX | 4.898812139 | 6.62E-30 |
| LOC6046433 | ficolin-3 | 4.873117266 | 7.67E-28 |
| LOC6041945 | hexamerin-1.1 | 5.661381594 | 2.96E-27 |
| LOC6051584 | larval cuticle protein A3A | 4.614772485 | 8.07E-26 |
| LOC6047742 | general odorant-binding protein 72 | 15.70247176 | 2.02E-25 |
| LOC119769321 | zonadhesin-like | 6.661773874 | 3.06E-25 |
| LOC6038231 | peptidoglycan recognition protein | 3.99744932 | 3.37E-24 |
| LOC6031357 | cuticle protein 38 | 9.522292206 | 5.09E-22 |
| LOC6043156 | chymotrypsin-C | 8.322500707 | 2.04E-21 |
| LOC6039374 | phenoloxidase 2 | -14.36653702 | 3.18E-21 |
| LOC6050894 | uncharacterized LOC6050894 | -3.975856555 | 3.96E-21 |
| LOC119768788 | uncharacterized LOC119768788 | -5.632549471 | 5.67E-21 |
| LOC119766624 | skin secretory protein xP2-like | 4.538493601 | 5.95E-21 |
| LOC6040262 | uncharacterized LOC6040262 | 5.677025003 | 6.57E-21 |
| LOC6037495 | probable cytochrome P450 6a14 | 3.718930505 | 1.93E-20 |
| LOC6042440 | uncharacterized LOC6042440 | 8.530182696 | 2.11E-20 |
| LOC6033003 | larval cuticle protein A3A | 9.401268982 | 2.15E-20 |
| LOC6040253 | uncharacterized LOC6040253 | 6.913053292 | 5.02E-20 |
| LOC6032999 | larval cuticle protein A2B | 9.202209894 | 5.72E-20 |
| LOC6043552 | microfibril-associated glycoprotein 4 | 13.70401532 | 8.70E-20 |
| LOC6052809 | cuticle protein 19.8 | 3.796793291 | 2.50E-19 |
| LOC119767898 | CLIP domain-containing serine protease 14D-like | 13.29281994 | 6.41E-19 |
| LOC6034355 | ejaculatory bulb-specific protein 3 | 3.408900318 | 1.18E-18 |
| LOC119766533 | cathepsin B-like | -9.532675231 | 1.89E-18 |
| LOC6037284 | receptor-like protein 9a | -2.239115087 | 2.25E-18 |
| LOC6048468 | protein LLP homolog | 2.033342747 | 2.55E-18 |
| LOC119765215 | uncharacterized LOC119765215 | 13.46911825 | 2.92E-18 |
| LOC119769981 | cuticle protein 16.5-like | 13.13769816 | 3.13E-18 |
| LOC6045847 | general odorant-binding protein 56d | 2.823449262 | 3.61E-18 |
| LOC6035742 | uncharacterized LOC6035742 | -4.919036567 | 5.73E-18 |
| LOC6050036 | uncharacterized LOC6050036 | -1.947679062 | 9.96E-18 |
| LOC119770564 | cathepsin B-like | -11.98551936 | 1.77E-17 |
| LOC6047890 | uncharacterized LOC6047890 | 5.812976452 | 1.96E-17 |
| LOC6045845 | phenoloxidase-activating factor 2 | 12.84891239 | 3.08E-17 |
| LOC119767712 | zinc finger CCHC domain-containing protein 24-like | 2.888412218 | 3.67E-17 |
| LOC6035994 | nidogen | -2.091735694 | 4.32E-17 |
| LOC6038733 | uncharacterized LOC6038733 | 5.635331119 | 4.78E-17 |
| LOC6049723 | nucleolin | 2.491598005 | 5.61E-17 |
| LOC119765090 | uncharacterized LOC119765090 | 8.48110278 | 7.65E-17 |
| LOC6040713 | uncharacterized LOC6040713 | 7.53454282 | 1.62E-16 |
| LOC6042326 | uncharacterized LOC6042326 | -3.093031723 | 1.69E-16 |
| LOC6043264 | probable cytochrome P450 9f2 | -12.35269133 | 2.75E-16 |
| LOC6044250 | ras-related and estrogen-regulated growth inhibitor-like protein | -4.023232637 | 3.49E-16 |
| LOC119766026 | uncharacterized LOC119766026 | -12.24543881 | 5.27E-16 |
| LOC6042434 | uncharacterized LOC6042434 | 12.52362596 | 8.44E-16 |
| LOC6046700 | larval cuticle protein A2B | 6.664516579 | 1.05E-15 |
| LOC119767980 | uncharacterized LOC119767980 | 3.158536415 | 1.48E-15 |
| LOC6037411 | uncharacterized LOC6037411 | -12.82655959 | 2.30E-15 |
| LOC6034616 | paternally-expressed gene 3 protein | 1.999008225 | 3.35E-15 |
| LOC6033001 | cuticle protein | 12.34050234 | 4.84E-15 |
| LOC6037494 | probable cytochrome P450 6a14 | 3.022064661 | 6.63E-15 |
| LOC6035809 | larval cuticle protein A2B | 7.464199937 | 7.08E-15 |
| LOC6039872 | uncharacterized LOC6039872 | 11.86404854 | 9.92E-15 |
| LOC119766799 | uncharacterized LOC119766799 | 7.676932565 | 2.12E-14 |
| LOC6035481 | mucin-5AC | 3.925837687 | 2.12E-14 |
| LOC119767852 | cuticle protein 16.5-like | 10.09547168 | 2.54E-14 |
| LOC6032243 | extensin | 6.036076022 | 2.64E-14 |
| LOC6051867 | histidine-rich protein PFHRP-II | 3.299130964 | 2.97E-14 |
| LOC119766095 | uncharacterized LOC119766095 | -11.67425085 | 5.11E-14 |
| LOC6050891 | uncharacterized LOC6050891 | -2.551509839 | 7.33E-14 |
| LOC6040251 | uncharacterized LOC6040251 | 6.735644587 | 2.51E-13 |
| LOC6032993 | larval cuticle protein A3A | 11.77879794 | 2.57E-13 |
| LOC6045354 | larval cuticle protein 1 | 3.696734622 | 3.03E-13 |
| LOC6034369 | ejaculatory bulb-specific protein 3 | 4.037429812 | 3.38E-13 |
| LOC6035813 | larval cuticle protein A2B | 5.645238878 | 3.82E-13 |
| LOC119769666 | coiled-coil domain-containing protein 1 | 3.951453404 | 4.85E-13 |
| LOC6051327 | myosin regulatory light chain 2 | 1.693687689 | 8.83E-13 |
| LOC6048226 | antigen 5 like allergen Cul n 1 | 5.187727124 | 1.33E-12 |
| LOC6033118 | cytochrome B5-like protein | -2.856261802 | 1.45E-12 |
| LOC6032559 | vitellogenin-A1 | -7.794050539 | 1.46E-12 |
| LOC6053712 | plant intracellular Ras-group-related LRR protein 3 | 4.493856293 | 2.12E-12 |
| LOC6036475 | uncharacterized LOC6036475 | 11.98493637 | 2.57E-12 |
| LOC119770219 | uncharacterized LOC119770219 | -11.25911844 | 2.80E-12 |
| LOC119766517 | uncharacterized LOC119766517 | 11.37545461 | 3.37E-12 |
| LOC6032238 | uncharacterized LOC6032238 | 2.519895611 | 3.99E-12 |
| LOC6046699 | cuticle protein 8 | 11.24992264 | 5.67E-12 |
| LOC6032136 | kynurenine formamidase | 3.124475358 | 8.32E-12 |
| LOC6037771 | L-galactose dehydrogenase | -7.477296699 | 8.39E-12 |
| LOC6049222 | cathepsin B | -7.941185121 | 9.94E-12 |
| LOC119767981 | uncharacterized LOC119767981 | 7.795300595 | 1.08E-11 |
| LOC119768891 | PR domain zinc finger protein 2-like | -2.143716617 | 1.37E-11 |
| LOC6043252 | vitellogenin-A1 | -9.652566543 | 1.42E-11 |
| LOC119766096 | uncharacterized LOC119766096 | -9.247013381 | 1.58E-11 |
| LOC119767907 | uncharacterized LOC119767907 | -10.85576057 | 2.24E-11 |
| LOC6049186 | cytochrome c oxidase subunit 7A, mitochondrial | 1.78264232 | 2.48E-11 |
| LOC6034664 | DNA-directed RNA polymerase II subunit RPB1 | 3.014232738 | 2.70E-11 |
| LOC6037602 | hemicentin-1 | -4.290754102 | 2.70E-11 |
| LOC6030831 | esterase B1 | -10.79546339 | 2.91E-11 |
| LOC6052229 | fumarylacetoacetase | -1.529554086 | 4.31E-11 |
| LOC6047250 | esterase B1 | 2.471656794 | 4.97E-11 |
| LOC119769493 | glycine-rich cell wall structural protein | 3.222618326 | 5.67E-11 |
| LOC6042426 | putative aminopeptidase W07G4.4 | -2.511103032 | 5.99E-11 |
| LOC6043429 | 40S ribosomal protein S2 | 1.734894438 | 6.13E-11 |
| LOC6037887 | transcription factor MafB | -5.387300542 | 6.66E-11 |
| LOC119767396 | apolipoprotein D-like | 3.741955199 | 7.14E-11 |
| LOC119767853 | DNA-directed RNA polymerase II subunit RPB1-like | 4.341958324 | 7.37E-11 |
| LOC6040913 | dynein light chain 1, axonemal | -8.990120982 | 1.21E-10 |
| LOC6043039 | ryncolin-4 | 5.830512627 | 1.21E-10 |
| LOC119770675 | uncharacterized LOC119770675 | 10.75477228 | 1.40E-10 |
| LOC119766528 | cathepsin B-like | -7.691854493 | 1.43E-10 |
| LOC119770282 | uncharacterized LOC119770282 | 1.511901127 | 2.23E-10 |
| LOC6047249 | esterase B1 | 3.470969939 | 2.62E-10 |
| LOC6050883 | kynurenine 3-monooxygenase | -3.681992751 | 3.11E-10 |
| LOC6035128 | uncharacterized LOC6035128 | -10.48748371 | 3.68E-10 |
| LOC6054693 | glycogen debranching enzyme | -1.725435117 | 4.44E-10 |
| LOC6047589 | ribosome production factor 2 homolog | 2.066136039 | 4.49E-10 |
| LOC119765617 | uncharacterized LOC119765617 | -10.45933068 | 5.16E-10 |
| LOC6043884 | uncharacterized LOC6043884 | 1.338214739 | 5.43E-10 |
| LOC6053051 | cytochrome c-2 | 1.696849913 | 5.43E-10 |
| LOC119765295 | uncharacterized LOC119765295 | -10.42278802 | 6.94E-10 |
| LOC119765062 | uncharacterized LOC119765062 | -10.56346952 | 7.18E-10 |
| LOC6047266 | xanthine dehydrogenase/oxidase | 5.045834661 | 8.36E-10 |
| LOC119769628 | uncharacterized LOC119769628 | -10.43444344 | 8.75E-10 |
| LOC6040134 | 60S ribosomal protein L37a | 1.43489505 | 8.92E-10 |
| LOC6051506 | probable cytochrome P450 6a13 | -10.3261339 | 9.73E-10 |
| LOC119765139 | uncharacterized LOC119765139 | -2.996207886 | 1.22E-09 |
| LOC6034842 | adult cuticle protein 1 | 3.550917626 | 1.22E-09 |
| LOC6034088 | ecdysone-induced protein 74EF | -7.104730088 | 1.33E-09 |
| LOC6042062 | cuticle protein 65 | 2.710412487 | 1.35E-09 |
| LOC6042962 | uncharacterized LOC6042962 | 10.5274014 | 1.36E-09 |
| LOC119769786 | uncharacterized LOC119769786 | 10.38999812 | 1.56E-09 |
| LOC6033979 | eukaryotic translation initiation factor 3 subunit A | -1.486262426 | 1.60E-09 |
| LOC119768899 | uncharacterized LOC119768899 | -10.32307111 | 1.70E-09 |
| LOC6051852 | selenide, water dikinase 2 | 1.393241669 | 1.72E-09 |
| LOC6031912 | H/ACA ribonucleoprotein complex subunit 4 | 2.520011474 | 1.73E-09 |
| LOC119769697 | uncharacterized LOC119769697 | 3.406059329 | 2.06E-09 |
| LOC119770460 | uncharacterized LOC119770460 | -8.501569103 | 2.06E-09 |
| LOC119766798 | basement membrane proteoglycan-like | -1.821197023 | 2.17E-09 |
| LOC6043117 | ornithine decarboxylase | -2.462959718 | 2.17E-09 |
| LOC119769968 | cuticle protein 12.5-like | 10.83734734 | 2.23E-09 |
| LOC119769640 | uncharacterized LOC119769640 | -10.23645037 | 2.45E-09 |
| LOC6035646 | cilia- and flagella-associated protein 58 | 5.121669385 | 2.83E-09 |
| LOC6051965 | uncharacterized LOC6051965 | 2.43227326 | 2.83E-09 |
| LOC6043250 | vitellogenin-A1 | -9.889179908 | 2.96E-09 |
| LOC119768039 | uncharacterized LOC119768039 | -10.16182068 | 3.15E-09 |
| LOC6039471 | poly(A) RNA polymerase gld-2 homolog B | -1.444589461 | 3.45E-09 |
| LOC6036898 | H/ACA ribonucleoprotein complex subunit 2-like protein | 2.062921886 | 3.53E-09 |
| LOC6041565 | short/branched chain specific acyl-CoA dehydrogenase, mitochondrial | 2.313889329 | 3.70E-09 |
| LOC119768829 | uncharacterized LOC119768829 | -10.08537509 | 3.74E-09 |
| LOC6036573 | pescadillo homolog | 1.217491198 | 4.13E-09 |
| LOC119770815 | putative uncharacterized protein DDB_G0271606 | 4.486374213 | 4.43E-09 |
| LOC6047405 | myrosinase 1 | 2.532625203 | 4.43E-09 |
| LOC6041540 | homocysteine S-methyltransferase | -3.120349817 | 4.76E-09 |
| LOC119770180 | uncharacterized LOC119770180 | 10.19417545 | 5.19E-09 |
| LOC6043895 | uncharacterized LOC6043895 | 5.618450511 | 5.34E-09 |
| LOC6045611 | proteoglycan 4 | -1.547207184 | 5.74E-09 |
| LOC6031630 | phenoloxidase-activating factor 3 | 1.989377408 | 6.09E-09 |
| LOC6031019 | probable cytochrome P450 308a1 | -1.359958822 | 7.14E-09 |
| LOC6033002 | larval cuticle protein A2B | 7.040335319 | 7.43E-09 |
| LOC6044950 | ribosomal protein S6 kinase alpha-5 | -1.845000555 | 7.46E-09 |
| LOC119766603 | uncharacterized LOC119766603 | -10.64068336 | 7.77E-09 |
| LOC6045132 | trimeric intracellular cation channel type 1B.1 | 1.428581235 | 7.84E-09 |
| LOC119769830 | cuticle protein 21-like | 10.17405757 | 9.06E-09 |
| LOC119765404 | uncharacterized LOC119765404 | 1.347434223 | 9.22E-09 |
| LOC6039323 | 10 kDa heat shock protein, mitochondrial | 2.116329709 | 9.67E-09 |
| LOC6044253 | phospholipase A2 group XV | -6.798291962 | 1.03E-08 |
| LOC119770717 | uncharacterized LOC119770717 | -9.971635172 | 1.06E-08 |
| LOC119769247 | uncharacterized LOC119769247 | -5.217678765 | 1.18E-08 |
| LOC6033165 | general odorant-binding protein 56d | -2.25090426 | 1.18E-08 |
| LOC6036347 | troponin C | 2.089687871 | 1.21E-08 |
| LOC6040955 | muscle LIM protein 1 | 1.182190684 | 1.23E-08 |
| LOC6037992 | NHP2-like protein 1 homolog | 3.01237996 | 1.24E-08 |
| LOC119765226 | N-alpha-acetyltransferase 80-like | 2.389837572 | 1.44E-08 |
| LOC119770586 | cuticle protein 16.5-like | 10.10292087 | 1.51E-08 |
| LOC6041411 | complement component 1 Q subcomponent-binding protein, mitochondrial | 1.515467939 | 1.56E-08 |
| LOC6049801 | flexible cuticle protein 12 | 9.990681213 | 1.64E-08 |
| LOC6037396 | guanine nucleotide-binding protein-like 3 homolog | 1.23282315 | 1.67E-08 |
| LOC119767054 | uncharacterized LOC119767054 | -9.842808577 | 1.87E-08 |
| LOC6038058 | C-terminal-binding protein | 1.075823008 | 1.96E-08 |
| LOC6050563 | uncharacterized LOC6050563 | -10.08080183 | 1.96E-08 |
| LOC6054403 | polyserase-2 | 3.51437647 | 1.96E-08 |
| LOC6042239 | DNA topoisomerase 2 | -1.422469477 | 2.06E-08 |
| LOC119770742 | sentrin-specific protease-like | 2.246722088 | 2.08E-08 |
| LOC6034827 | uncharacterized LOC6034827 | 9.909486671 | 2.37E-08 |
| LOC6042504 | uncharacterized LOC6042504 | -9.821187377 | 2.42E-08 |
| LOC6047137 | tropomyosin-1 | 1.464552232 | 2.63E-08 |
| LOC119770892 | 5.8S ribosomal RNA | 2.300102296 | 2.65E-08 |
| LOC6052013 | uncharacterized LOC6052013 | -2.949481104 | 2.94E-08 |
| LOC119765335 | uncharacterized LOC119765335 | 3.86769468 | 3.35E-08 |
| LOC6031554 | cathepsin B | -9.712749783 | 3.45E-08 |
| LOC6035189 | protein cereblon | -1.795905855 | 3.45E-08 |
| LOC6053801 | uncharacterized LOC6053801 | 5.402185873 | 3.45E-08 |
| LOC6040012 | glia maturation factor gamma | 1.730555132 | 3.54E-08 |
| LOC119769857 | cuticle protein 38-like | 9.961200933 | 3.78E-08 |
| LOC119769027 | uncharacterized protein K02A2.6-like | -9.777894425 | 4.11E-08 |
| LOC119768985 | uncharacterized protein K02A2.6-like | -9.993456363 | 4.53E-08 |
| LOC6047672 | alpha-L-iduronidase | -5.222019065 | 4.64E-08 |
| LOC6049674 | uncharacterized LOC6049674 | 6.353111357 | 4.64E-08 |
| LOC6035103 | forkhead box protein K1 | 1.812841166 | 4.72E-08 |
| LOC6036392 | uncharacterized LOC6036392 | 2.419206399 | 4.74E-08 |
| LOC6038227 | peptidoglycan-recognition protein LA | -2.223829058 | 4.88E-08 |
| LOC6037336 | probable ATP-dependent RNA helicase DDX43 | -1.503977524 | 5.09E-08 |
| LOC6032965 | carbonic anhydrase 2 | 1.149218166 | 5.13E-08 |
| LOC6030842 | hexamerin-1.1 | 4.060295812 | 5.19E-08 |
| LOC6034666 | cuticle protein 16.5 | 2.983246786 | 5.62E-08 |
| LOC6035344 | nucleoplasmin-like protein | 1.53042798 | 5.84E-08 |
| LOC6042095 | endocuticle structural glycoprotein ABD-4 | 3.976180728 | 5.86E-08 |
| LOC6042072 | proline-rich protein 4 | 3.926137923 | 6.16E-08 |
| LOC6049236 | histone H3.v1 | 3.045497253 | 6.16E-08 |
| LOC6048459 | 40S ribosomal protein SA | 1.439360756 | 6.83E-08 |
| LOC6037519 | calexcitin-1 | 1.372749483 | 6.90E-08 |
| LOC119770359 | uncharacterized protein K02A2.6-like | -9.754091353 | 6.92E-08 |
| LOC6037632 | uncharacterized LOC6037632 | -6.510787033 | 7.00E-08 |
| LOC119770378 | uncharacterized LOC119770378 | -9.67022857 | 7.48E-08 |
| LOC6054402 | lactoylglutathione lyase | 1.322038644 | 8.08E-08 |
| LOC6039660 | alpha-catulin | -1.764177053 | 8.37E-08 |
| LOC6044205 | uncharacterized LOC6044205 | 3.921080565 | 9.32E-08 |
| LOC6042208 | 60S ribosomal protein L44 | 1.398971086 | 9.46E-08 |
| LOC6038275 | 40S ribosomal protein S11 | 1.425200955 | 9.48E-08 |
| LOC6050293 | triokinase/FMN cyclase | -2.432477436 | 9.91E-08 |
| LOC6042063 | cuticle protein 12.5 | 1.60689743 | 1.03E-07 |
| LOC6035412 | saccharopine dehydrogenase-like oxidoreductase | -2.077801005 | 1.05E-07 |
| LOC6048310 | cuticle protein 16.5 | 5.246384889 | 1.06E-07 |
| LOC6049115 | CD209 antigen | 9.921527094 | 1.08E-07 |
| LOC119765683 | uncharacterized LOC119765683 | -9.69354717 | 1.11E-07 |
| LOC6050002 | mucin-2 | 3.276844295 | 1.12E-07 |
| LOC119769273 | uncharacterized LOC119769273 | 6.925634642 | 1.13E-07 |
| LOC6038340 | 40S ribosomal protein S20 | 1.557794719 | 1.22E-07 |
| LOC6033032 | NADH dehydrogenase [ubiquinone] 1 beta subcomplex subunit 8, mitochondrial | 1.086632432 | 1.26E-07 |
| LOC6032821 | uncharacterized LOC6032821 | 6.114861215 | 1.28E-07 |
| LOC6044075 | putative carbonic anhydrase 3 | 1.485791693 | 1.51E-07 |
| LOC6049754 | uncharacterized LOC6049754 | 9.587546161 | 1.60E-07 |
| LOC6039201 | TRAF3-interacting protein 1 | -2.039185771 | 1.79E-07 |
| LOC6050933 | Ca(2+)/calmodulin-responsive adenylate cyclase | -1.441500453 | 1.79E-07 |
| LOC119770670 | uncharacterized LOC119770670 | 6.023468051 | 1.87E-07 |
| LOC6054413 | cysteine dioxygenase 1 | 2.374843711 | 1.88E-07 |
| LOC119768917 | uncharacterized LOC119768917 | 3.671770989 | 1.95E-07 |
| LOC6035686 | uncharacterized LOC6035686 | -1.980132669 | 1.95E-07 |
| LOC119769599 | uncharacterized protein K02A2.6-like | 1.583547812 | 2.10E-07 |
| LOC6053603 | ribosomal RNA-processing protein 8 | 2.20492138 | 2.12E-07 |
| LOC6036217 | ribose-phosphate pyrophosphokinase 1 | 1.640839804 | 2.27E-07 |
| LOC6038688 | uncharacterized LOC6038688 | -2.273190388 | 2.47E-07 |
| LOC6051129 | basic salivary proline-rich protein 2 | -1.20485907 | 2.66E-07 |
| LOC6040349 | dynein heavy chain, cytoplasmic | -1.036064662 | 2.70E-07 |
| LOC6031538 | uncharacterized LOC6031538 | 1.826327074 | 2.83E-07 |
| LOC6032286 | putative helicase MOV-10 | -3.1418787 | 2.87E-07 |
| LOC6051663 | mediator of RNA polymerase II transcription subunit 25 | -1.916983207 | 2.90E-07 |
| LOC6031976 | probable DNA-directed RNA polymerases I and III subunit RPAC2 | 1.937996374 | 2.93E-07 |
| LOC6035616 | paramyosin, long form | 1.213020998 | 3.07E-07 |
| LOC6036550 | uncharacterized LOC6036550 | 3.42099879 | 3.10E-07 |
| LOC119768696 | uncharacterized LOC119768696 | 9.551793117 | 3.16E-07 |
| LOC119765102 | uncharacterized LOC119765102 | -9.704569247 | 3.22E-07 |
| LOC6035536 | opsin-1 | -2.071288265 | 3.25E-07 |
| LOC6036016 | paired amphipathic helix protein Sin3a | -1.068682403 | 3.30E-07 |
| LOC6033399 | protein MMS22-like | -2.826367133 | 3.31E-07 |
| LOC119766994 | uncharacterized LOC119766994 | -1.456598858 | 3.36E-07 |
| LOC6032802 | venom carboxylesterase-6 | 2.168194421 | 3.73E-07 |
| LOC119771118 | uncharacterized protein K02A2.6-like | 3.658679729 | 3.79E-07 |
| LOC6035075 | heterogeneous nuclear ribonucleoprotein Q | 1.524238438 | 3.99E-07 |
| LOC6046589 | aminoacylase-1B | -1.921725932 | 3.99E-07 |
| LOC6033671 | probable transcriptional regulatory protein TTE1135 | 1.477752086 | 4.04E-07 |
| LOC6033345 | antigen 5 like allergen Cul n 1 | 2.439562608 | 4.12E-07 |
| LOC6035113 | uncharacterized LOC6035113 | -9.25225443 | 4.23E-07 |
| LOC6045346 | calponin homology domain-containing protein DDB_G0272472 | 3.604026676 | 4.27E-07 |
| LOC6031556 | cathepsin B | -8.997446875 | 4.34E-07 |
| LOC6052976 | cuticle protein 8 | 8.540830818 | 4.35E-07 |
| LOC6039526 | regulator of gene activity | -1.231278463 | 4.43E-07 |
| LOC6052150 | serine protease snake | 1.476794201 | 4.43E-07 |
| LOC6042026 | protein TRC8 homolog | -1.623582536 | 4.48E-07 |
| LOC119768049 | uncharacterized LOC119768049 | -9.238310862 | 4.55E-07 |
| LOC6032982 | hexamerin-1.1 | 4.680746718 | 4.77E-07 |
| LOC6039734 | hexamerin-1.1 | 7.434231067 | 4.91E-07 |
| LOC6034384 | integrator complex subunit 1 | -1.2455261 | 5.09E-07 |
| LOC6046478 | myosin light chain alkali | 1.245747772 | 6.22E-07 |
| LOC6045598 | facilitated trehalose transporter Tret1 | -1.264910928 | 6.61E-07 |
| LOC6042074 | repetitive proline-rich cell wall protein 2 | 2.19898716 | 6.69E-07 |
| LOC6038431 | 40S ribosomal protein S15Aa | 1.140942714 | 6.85E-07 |
| LOC6042082 | fibrillin-1 | -3.233130457 | 7.01E-07 |
| LOC6052597 | 60S ribosomal protein L10 | 1.156294381 | 7.12E-07 |
| LOC119770320 | uncharacterized LOC119770320 | -9.171015587 | 7.17E-07 |
| LOC119766756 | uncharacterized LOC119766756 | 7.778097083 | 7.65E-07 |
| LOC6042564 | uncharacterized LOC6042564 | 1.454047884 | 7.65E-07 |
| LOC6036305 | uncharacterized LOC6036305 | -2.112846948 | 7.78E-07 |
| LOC6045552 | von Willebrand factor D and EGF domain-containing protein | 3.564631838 | 7.78E-07 |
| LOC6049721 | uncharacterized LOC6049721 | -1.627737665 | 8.31E-07 |
| LOC119767347 | protein FAM32A-like | 1.349349514 | 8.86E-07 |
| LOC6043021 | proteasome subunit alpha type-5 | 1.161522106 | 8.86E-07 |
| LOC119765657 | protein bangles and beads | 1.298271492 | 9.18E-07 |
| LOC119768711 | uncharacterized LOC119768711 | -9.170404472 | 1.04E-06 |
| LOC119770237 | uncharacterized LOC119770237 | -3.530145218 | 1.09E-06 |
| LOC6049791 | endocuticle structural glycoprotein SgAbd-5 | 1.617020184 | 1.12E-06 |
| LOC6045499 | ribosome biogenesis protein BRX1 homolog | 1.576760389 | 1.18E-06 |
| LOC6053278 | general odorant-binding protein 56d | 2.242599687 | 1.20E-06 |
| LOC119769520 | probable methyltransferase-like protein 15 homolog | 1.478368376 | 1.25E-06 |
| LOC6044517 | polyserase-2 | -9.163254705 | 1.25E-06 |
| LOC119766646 | uncharacterized LOC119766646 | -2.68308723 | 1.29E-06 |
| LOC6033770 | uncharacterized LOC6033770 | -1.001934184 | 1.29E-06 |
| LOC6036078 | acetylcholinesterase | 2.51837852 | 1.34E-06 |
| LOC6047714 | troponin I | 1.177721977 | 1.41E-06 |
| LOC6035808 | cuticle protein 7 | 3.592750871 | 1.44E-06 |
| LOC6035680 | nucleolar protein dao-5 | 1.035924897 | 1.49E-06 |
| LOC6043140 | carboxypeptidase B | 2.703624485 | 1.52E-06 |
| LOC6031168 | CD109 antigen | 2.208451118 | 1.53E-06 |
| LOC6041686 | cuticle protein 16.5 | 9.235243705 | 1.57E-06 |
| LOC119766412 | uncharacterized protein K02A2.6-like | -9.065402579 | 1.59E-06 |
| LOC6034881 | signal-induced proliferation-associated 1-like protein 2 | -1.17777935 | 1.60E-06 |
| LOC119767684 | succinate dehydrogenase cytochrome b560 subunit, mitochondrial-like | 7.550280046 | 1.61E-06 |
| LOC6034116 | ribosome biogenesis protein NSA2 homolog | 1.496080225 | 1.64E-06 |
| LOC6049436 | lysosomal alpha-mannosidase | -1.313352235 | 1.64E-06 |
| LOC6039172 | uncharacterized LOC6039172 | -9.150215098 | 1.68E-06 |
| LOC6033417 | uncharacterized LOC6033417 | -1.823174093 | 1.81E-06 |
| LOC6052243 | ATP-binding cassette sub-family B member 6, mitochondrial | -1.006630739 | 1.87E-06 |
| LOC6037321 | zinc finger protein 593 homolog | 1.447904055 | 1.92E-06 |
| LOC6042975 | protein Gawky | 1.012178282 | 1.96E-06 |
| LOC119767177 | uncharacterized LOC119767177 | -9.085299853 | 1.98E-06 |
| LOC6031746 | trans-1,2-dihydrobenzene-1,2-diol dehydrogenase | -9.067937489 | 1.98E-06 |
| LOC6038440 | EF-hand calcium-binding domain-containing protein 1 | -8.995848565 | 2.00E-06 |
| LOC6033111 | myosin-VIIa | -3.615852527 | 2.13E-06 |
| LOC6032797 | queuosine salvage protein | 1.818848721 | 2.18E-06 |
| LOC6031440 | protein obstructor-E | 2.592432661 | 2.27E-06 |
| LOC6047916 | omega-amidase NIT2 | -1.739894181 | 2.40E-06 |
| LOC6031860 | CKLF-like MARVEL transmembrane domain-containing protein 4 | 1.472327176 | 2.40E-06 |
| LOC6037479 | ADP,ATP carrier protein 2 | 1.573309521 | 2.41E-06 |
| LOC6036729 | bifunctional methylenetetrahydrofolate dehydrogenase/cyclohydrolase, mitochondrial | 2.25746894 | 2.43E-06 |
| LOC6038486 | zinc finger SWIM domain-containing protein 8 | -1.809375844 | 2.48E-06 |
| LOC6035171 | serine protease grass | 2.939698814 | 2.55E-06 |
| LOC119765980 | uncharacterized LOC119765980 | -8.951274343 | 2.55E-06 |
| LOC6032249 | 40S ribosomal protein S23 | 1.350673229 | 2.69E-06 |
| LOC6036970 | N-acetyl-D-glucosamine kinase | -1.092651728 | 2.69E-06 |
| LOC119770288 | uncharacterized LOC119770288 | 1.47271561 | 2.77E-06 |
| LOC6050064 | DNA-directed RNA polymerase II subunit RPB1 | -1.042563413 | 2.88E-06 |
| LOC6035805 | neural/ectodermal development factor IMP-L2 | 1.297225645 | 2.91E-06 |
| LOC6046936 | O-acyltransferase like protein | -3.455957507 | 2.91E-06 |
| LOC6038919 | uncharacterized LOC6038919 | -8.885930573 | 3.03E-06 |
| LOC6040257 | uncharacterized LOC6040257 | 4.844833761 | 3.08E-06 |
| LOC119769054 | NADH-ubiquinone oxidoreductase chain 6-like | 1.36269102 | 3.09E-06 |
| LOC6040008 | acetylcholinesterase | -2.61344385 | 3.09E-06 |
| LOC119766148 | uncharacterized LOC119766148 | 7.439198537 | 3.21E-06 |
| LOC6035564 | trypsin-1 | 2.880866872 | 3.21E-06 |
| LOC6038051 | uncharacterized LOC6038051 | -1.745625669 | 3.28E-06 |
| LOC6050707 | cytochrome P450 6a8 | 2.26728819 | 3.34E-06 |
| LOC119765488 | zinc finger protein 16-like | 4.484314008 | 3.48E-06 |
| LOC119768969 | uncharacterized LOC119768969 | 6.061851041 | 3.48E-06 |
| LOC119768106 | ATP-dependent helicase brm | -1.173181954 | 3.70E-06 |
| LOC6046236 | sodium-independent sulfate anion transporter | -2.893575327 | 3.76E-06 |
| LOC119770491 | microfibril-associated glycoprotein 4-like | 1.942342745 | 3.92E-06 |
| LOC6042432 | uncharacterized LOC6042432 | 3.63724977 | 3.98E-06 |
| LOC6052852 | hexamerin-1.1 | 3.679853019 | 3.98E-06 |
| LOC6043892 | allatostatin-A receptor | -2.637577257 | 4.34E-06 |
| LOC6032252 | 60S ribosomal protein L32 | 1.167407576 | 4.47E-06 |
| LOC6031460 | uncharacterized LOC6031460 | 1.444449109 | 4.53E-06 |
| LOC6034631 | adult-specific cuticular protein ACP-20 | 2.703996388 | 4.97E-06 |
| LOC6033780 | uncharacterized LOC6033780 | 2.461917194 | 5.89E-06 |
| LOC6032891 | protein henna | 1.333151566 | 5.99E-06 |
| LOC119766511 | uncharacterized LOC119766511 | -8.870552885 | 6.47E-06 |
| LOC6036442 | repressor of RNA polymerase III transcription MAF1 homolog | -1.17244749 | 6.61E-06 |
| LOC6042717 | uncharacterized LOC6042717 | 2.796169602 | 6.61E-06 |
| LOC119767593 | uncharacterized LOC119767593 | -8.740585058 | 6.75E-06 |
| LOC6041884 | probable 60S ribosomal protein L37-A | 1.233787382 | 6.86E-06 |
| LOC119765278 | uncharacterized LOC119765278 | -1.61891871 | 7.29E-06 |
| LOC6046146 | endochitinase | -1.582144005 | 7.91E-06 |
| LOC6033682 | sodium-coupled monocarboxylate transporter 1 | 1.836110265 | 7.96E-06 |
| LOC6031287 | C-type lectin 37Da | 8.852137136 | 8.19E-06 |
| LOC6032269 | transcription factor GAGA | -1.802528951 | 8.63E-06 |
| LOC6042364 | structural maintenance of chromosomes protein 2 | -1.528551318 | 8.92E-06 |
| LOC6043699 | 60S ribosomal protein L14 | 1.057644417 | 8.92E-06 |
| LOC119769450 | uncharacterized LOC119769450 | -8.821979586 | 8.98E-06 |
| LOC6037346 | actin-87E | 1.194049927 | 9.05E-06 |
| LOC6048151 | protein argonaute-2 | -1.674131504 | 9.13E-06 |
| LOC119769719 | cuticle protein 38-like | 3.872025215 | 9.32E-06 |
| LOC6051097 | uncharacterized LOC6051097 | -8.985278529 | 1.08E-05 |
| LOC6042433 | uncharacterized LOC6042433 | 3.572169765 | 1.08E-05 |
| LOC6033704 | bifunctional purine biosynthesis protein ATIC | 1.190928341 | 1.11E-05 |
| LOC6048453 | transmembrane protease serine 9 | 3.673910135 | 1.16E-05 |
| LOC6034147 | translation machinery-associated protein 7 homolog | 1.283632289 | 1.18E-05 |
| LOC119765418 | uncharacterized LOC119765418 | 4.951171576 | 1.22E-05 |
| LOC6032731 | ecdysone 20-monooxygenase | 1.92266833 | 1.28E-05 |
| LOC6045183 | eukaryotic translation initiation factor 6 | 1.750368929 | 1.28E-05 |
| LOC6034434 | mpv17-like protein | 1.571188407 | 1.30E-05 |
| LOC6044737 | opsin-1 | 1.798177248 | 1.31E-05 |
| LOC6045595 | serine--pyruvate aminotransferase, mitochondrial | -1.686081289 | 1.33E-05 |
| LOC6037019 | uncharacterized LOC6037019 | 3.214716763 | 1.36E-05 |
| LOC6035807 | cuticle protein 21 | 8.543147709 | 1.43E-05 |
| LOC6043925 | chitotriosidase-1 | 5.163074049 | 1.44E-05 |
| LOC6054142 | uncharacterized LOC6054142 | 1.694538863 | 1.46E-05 |
| LOC119769878 | uncharacterized protein K02A2.6-like | -5.413075222 | 1.46E-05 |
| LOC6047791 | sugar transporter SWEET1 | 3.192774328 | 1.49E-05 |
| LOC119769855 | cuticle protein 19-like | 9.844069321 | 1.50E-05 |
| LOC6033638 | 116 kDa U5 small nuclear ribonucleoprotein component | 8.746720123 | 1.51E-05 |
| LOC6047744 | putative mediator of RNA polymerase II transcription subunit 26 | -1.975945583 | 1.52E-05 |
| LOC119769267 | uncharacterized LOC119769267 | -8.671443445 | 1.53E-05 |
| LOC6040004 | caspase-1 | -8.683296722 | 1.53E-05 |
| LOC6049371 | 3-oxoacyl-[acyl-carrier-protein] reductase FabG | 2.178875339 | 1.55E-05 |
| LOC6052583 | DNA-directed RNA polymerases I, II, and III subunit RPABC2 | 1.370091782 | 1.58E-05 |
| LOC6039221 | probable 28S ribosomal protein S6, mitochondrial | 1.509743772 | 1.59E-05 |
| LOC6033160 | uncharacterized LOC6033160 | -2.12323369 | 1.62E-05 |
| LOC6040567 | 40S ribosomal protein S12 | 1.202981929 | 1.68E-05 |
| LOC6044195 | uncharacterized LOC6044195 | -3.119151949 | 1.68E-05 |
| LOC6047052 | 60S acidic ribosomal protein P2 | 1.324006432 | 1.69E-05 |
| LOC6036275 | 60S ribosomal protein L29 | 1.16888194 | 1.78E-05 |
| LOC6044030 | probable serine/threonine-protein kinase kinX | -1.358427268 | 1.78E-05 |
| LOC6043330 | maternal protein exuperantia | -3.871045135 | 1.81E-05 |
| LOC6053871 | probable maltase | 2.024784374 | 1.81E-05 |
| LOC119765381 | uncharacterized LOC119765381 | -8.608330512 | 1.83E-05 |
| LOC119767966 | uncharacterized LOC119767966 | 8.670512534 | 1.84E-05 |
| LOC6037215 | probable prefoldin subunit 6 | 1.19934486 | 1.84E-05 |
| LOC6048183 | 40S ribosomal protein S13 | 1.061376741 | 1.84E-05 |
| LOC6049167 | beta-lactamase-like protein 2 homolog | -1.27016341 | 1.88E-05 |
| LOC6050709 | probable cytochrome P450 6a14 | 2.873636517 | 1.93E-05 |
| LOC6031439 | protein obstructor-E | 3.13645393 | 1.93E-05 |
| LOC119770474 | uncharacterized LOC119770474 | -5.447414777 | 1.95E-05 |
| LOC6038921 | deoxynucleotidyltransferase terminal-interacting protein 2 | 1.552673011 | 2.11E-05 |
| LOC6042275 | carboxypeptidase D | -1.013592352 | 2.14E-05 |
| LOC6038061 | ER membrane protein complex subunit 6 | 1.211527768 | 2.17E-05 |
| LOC6036297 | farnesol dehydrogenase | 1.708636479 | 2.24E-05 |
| LOC6045998 | arrestin domain-containing protein 2 | -2.640573324 | 2.24E-05 |
| LOC6048522 | protein boule | 1.317193087 | 2.34E-05 |
| LOC119767019 | cytochrome c oxidase subunit 6A, mitochondrial-like | 1.258517933 | 2.38E-05 |
| LOC6035285 | lamin Dm0 | -9.12679355 | 2.52E-05 |
| LOC119766751 | uncharacterized LOC119766751 | -8.86343323 | 2.54E-05 |
| LOC6032416 | palmitoyltransferase Hip14 | -4.631614266 | 2.55E-05 |
| LOC119767517 | uncharacterized LOC119767517 | 2.504999528 | 2.62E-05 |
| LOC6038339 | 40S ribosomal protein S7 | 1.025826326 | 2.69E-05 |
| LOC6039379 | proteasome subunit alpha type-2 | 1.003604171 | 2.71E-05 |
| LOC6033157 | uncharacterized LOC6033157 | -2.140660285 | 2.73E-05 |
| LOC119767364 | uncharacterized LOC119767364 | -8.432283176 | 2.75E-05 |
| LOC119771189 | 4-coumarate--CoA ligase 1-like | -8.397975948 | 2.82E-05 |
| LOC119767806 | uncharacterized LOC119767806 | -8.699523441 | 2.83E-05 |
| LOC6036926 | uncharacterized LOC6036926 | 1.932517575 | 2.83E-05 |
| LOC119765548 | uncharacterized LOC119765548 | 8.904082192 | 2.86E-05 |
| LOC6036330 | carbohydrate sulfotransferase 13 | 1.474838964 | 2.86E-05 |
| LOC6043268 | kielin/chordin-like protein | 1.146333223 | 2.89E-05 |
| LOC6044791 | cytosolic endo-beta-N-acetylglucosaminidase | -1.979519593 | 2.89E-05 |
| LOC6052628 | transcriptional adapter 3 | 1.386332197 | 2.92E-05 |
| LOC6041757 | 46 kDa FK506-binding nuclear protein | 1.232340759 | 2.94E-05 |
| LOC6051202 | uncharacterized LOC6051202 | 1.060938275 | 2.95E-05 |
| LOC6040537 | mitochondrial import receptor subunit TOM20 homolog | 1.023156543 | 2.96E-05 |
| LOC119769445 | uncharacterized LOC119769445 | 1.236019129 | 3.13E-05 |
| LOC119769805 | uncharacterized LOC119769805 | -8.410034245 | 3.13E-05 |
| LOC119765081 | uncharacterized LOC119765081 | 9.236311269 | 3.18E-05 |
| LOC6033162 | uncharacterized LOC6033162 | 7.127273857 | 3.18E-05 |
| LOC6049618 | glycine N-methyltransferase | -1.704456323 | 3.21E-05 |
| LOC6051853 | histidine protein methyltransferase 1 homolog | 2.927741662 | 3.32E-05 |
| LOC119768997 | glycine-rich cell wall structural protein 1.8-like | -6.468068824 | 3.41E-05 |
| LOC6039047 | proton-coupled amino acid transporter 4 | 2.851841987 | 3.43E-05 |
| LOC6044008 | small ubiquitin-related modifier 3 | 1.155150122 | 3.47E-05 |
| LOC6036833 | protein hairy | 1.447669461 | 3.48E-05 |
| LOC119768670 | uncharacterized LOC119768670 | -8.340144439 | 3.61E-05 |
| LOC6030844 | tyrosine-protein kinase receptor torso | -2.679142748 | 3.61E-05 |
| LOC6046705 | larval cuticle protein A2B | 6.821589104 | 3.61E-05 |
| LOC6048844 | leucine-rich repeat-containing protein 57 | 1.096903673 | 3.61E-05 |
| LOC6050225 | actin, muscle | 1.320173033 | 3.67E-05 |
| LOC6039036 | uncharacterized LOC6039036 | -8.524999753 | 3.76E-05 |
| LOC6032307 | 60S ribosomal protein L10a | 1.077264109 | 3.83E-05 |
| LOC6032672 | RNA polymerase II degradation factor 1 | -1.497472213 | 3.87E-05 |
| LOC6046495 | senecionine N-oxygenase | -8.364260146 | 3.91E-05 |
| LOC6045038 | glutenin, high molecular weight subunit PW212 | -1.829776971 | 3.97E-05 |
| LOC6034839 | adult cuticle protein 1 | 2.824524582 | 3.97E-05 |
| LOC119766571 | uncharacterized LOC119766571 | -2.764281451 | 4.14E-05 |
| LOC6043484 | phosphoenolpyruvate carboxykinase [GTP] | 2.552561741 | 4.24E-05 |
| LOC6048537 | uncharacterized LOC6048537 | -1.235967176 | 4.24E-05 |
| LOC6031539 | uncharacterized LOC6031539 | 1.303607553 | 4.34E-05 |
| LOC6044594 | STE20-related kinase adapter protein alpha | 2.240434017 | 4.38E-05 |
| LOC6042849 | uncharacterized LOC6042849 | -1.255435002 | 4.43E-05 |
| LOC119769127 | uncharacterized LOC119769127 | 8.666770756 | 4.80E-05 |
| LOC119770827 | uncharacterized LOC119770827 | -5.245672936 | 4.85E-05 |
| LOC119771139 | speckle-type POZ protein-like | 2.386442659 | 4.93E-05 |
| LOC6041038 | sterol regulatory element-binding protein 2 | -1.240801334 | 4.96E-05 |
| LOC119770207 | uncharacterized LOC119770207 | -8.349878778 | 5.04E-05 |
| LOC119771083 | uncharacterized LOC119771083 | -8.310163978 | 5.04E-05 |
| LOC6032532 | serine/threonine-protein kinase/endoribonuclease IRE1 | -1.124881931 | 5.06E-05 |
| LOC6031536 | N-acylneuraminate cytidylyltransferase A | 3.846810768 | 5.27E-05 |
| LOC6050302 | uncharacterized LOC6050302 | 1.26615082 | 5.46E-05 |
| LOC119767381 | uncharacterized LOC119767381 | 5.489095312 | 5.53E-05 |
| LOC6039504 | gamma-tubulin complex component 5 | -1.454111267 | 5.65E-05 |
| LOC6039926 | aprataxin and PNK-like factor | -1.321383983 | 5.77E-05 |
| LOC6034117 | ubiquitin-40S ribosomal protein S27a | 1.108841805 | 5.89E-05 |
| LOC6042103 | uncharacterized LOC6042103 | -8.325047166 | 5.89E-05 |
| LOC6054224 | probable cytochrome P450 6a23 | 1.835250361 | 5.89E-05 |
| LOC6031767 | cytochrome b5-related protein | -1.112919457 | 6.45E-05 |
| LOC6048461 | uncharacterized LOC6048461 | 2.742478229 | 6.45E-05 |
| LOC6042079 | uncharacterized LOC6042079 | -1.867994477 | 6.56E-05 |
| LOC6049442 | uncharacterized LOC6049442 | 1.943457648 | 6.70E-05 |
| LOC119768849 | uncharacterized LOC119768849 | -8.174573588 | 6.71E-05 |
| LOC6041364 | ornithine decarboxylase 1 | 8.339590642 | 6.74E-05 |
| LOC6034628 | cuticle protein 19 | 4.908668389 | 6.78E-05 |
| LOC119767904 | nuclear polyadenylated RNA-binding protein 3-like | -1.793161597 | 6.82E-05 |
| LOC6040753 | protein spinster | -1.146289966 | 6.86E-05 |
| LOC119768741 | uncharacterized LOC119768741 | -4.275803248 | 6.89E-05 |
| LOC6039333 | uncharacterized LOC6039333 | 3.154211995 | 7.14E-05 |
| LOC6031732 | uncharacterized LOC6031732 | 1.561470513 | 7.17E-05 |
| LOC6036596 | putative odorant receptor 83c | 2.415414209 | 7.32E-05 |
| LOC6041917 | putative aldehyde dehydrogenase family 7 member A1 homolog | 1.194266498 | 7.32E-05 |
| LOC6039718 | KAT8 regulatory NSL complex subunit 3 | -1.0865358 | 7.38E-05 |
| LOC6042953 | proteasome-associated protein ECM29 homolog | -1.842576203 | 7.44E-05 |
| LOC6037828 | 60S ribosomal protein L27 | 1.016610473 | 7.50E-05 |
| LOC6033284 | protein MLP1 homolog | 1.217231831 | 7.60E-05 |
| LOC6049292 | uncharacterized LOC6049292 | -1.327014364 | 7.68E-05 |
| LOC6039397 | uncharacterized LOC6039397 | 1.630638723 | 8.13E-05 |
| LOC6048046 | protein ST7 homolog | -1.40406864 | 8.31E-05 |
| LOC6032829 | NADH dehydrogenase [ubiquinone] 1 beta subcomplex subunit 5, mitochondrial | 1.024733627 | 8.32E-05 |
| LOC6042108 | endocuticle structural glycoprotein SgAbd-2 | 2.914044924 | 8.66E-05 |
| LOC6033274 | dTTP/UTP pyrophosphatase | 2.253824082 | 8.78E-05 |
| LOC6043112 | 60S ribosomal protein L39 | 1.262580958 | 8.87E-05 |
| LOC6048695 | uncharacterized LOC6048695 | 2.714832067 | 9.06E-05 |
| LOC6038327 | ficolin-1 | 6.917831581 | 9.12E-05 |
| LOC6041912 | glutathione S-transferase 1-1 | 1.135598134 | 9.12E-05 |
| LOC6049171 | 40S ribosomal protein S28 | 1.280056072 | 9.17E-05 |
| LOC6039971 | protein takeout | 4.312844928 | 9.27E-05 |
| LOC6031148 | UDP-glycosyltransferase UGT4 | -1.817396198 | 9.58E-05 |
| LOC6046309 | Krueppel homolog 1 | 2.237118103 | 9.63E-05 |
| LOC6052698 | NADH dehydrogenase [ubiquinone] flavoprotein 2, mitochondrial | 1.107011883 | 9.63E-05 |
| LOC119770058 | uncharacterized LOC119770058 | -8.108226886 | 9.69E-05 |
| LOC6046148 | probable serine/threonine-protein kinase kinX | 1.04683498 | 9.96E-05 |
| LOC119765279 | peptidyl-prolyl cis-trans isomerase-like | 1.129815645 | 9.97E-05 |
| LOC6039222 | Bloom syndrome protein homolog | -1.721912361 | 0.000103414 |
| LOC6048346 | uncharacterized LOC6048346 | 1.023783646 | 0.000104396 |
| LOC119767803 | uncharacterized LOC119767803 | -4.039290769 | 0.000107972 |
| LOC119769696 | uncharacterized LOC119769696 | 8.474075135 | 0.00011128 |
| LOC6045169 | myelin expression factor 2 | -1.002748508 | 0.000111797 |
| LOC6048349 | protein spaetzle 4 | 1.324765254 | 0.000113718 |
| LOC6054219 | probable cytochrome P450 6a14 | -5.846895147 | 0.000113718 |
| LOC6048308 | neprilysin-4 | -1.511938147 | 0.00011601 |
| LOC6037642 | maltase A1 | -1.498904687 | 0.000117394 |
| LOC6047706 | TAR DNA-binding protein 43 | -1.293347077 | 0.000117529 |
| LOC119766585 | uncharacterized LOC119766585 | 4.405134381 | 0.000123334 |
| LOC6052930 | H/ACA ribonucleoprotein complex subunit 3 | 2.209275305 | 0.000123334 |
| LOC6039635 | uncharacterized LOC6039635 | -1.009185549 | 0.000124359 |
| LOC6049711 | pro-resilin | 1.827374783 | 0.000125079 |
| LOC119766839 | uncharacterized LOC119766839 | -6.444296141 | 0.00012546 |
| LOC6039174 | putative hydroxypyruvate isomerase | -1.203534729 | 0.000125637 |
| LOC6042421 | troponin T, skeletal muscle | 1.014834613 | 0.000125776 |
| LOC6032792 | 39S ribosomal protein L42, mitochondrial | 1.391775436 | 0.000130219 |
| LOC6043914 | X-ray repair cross-complementing protein 5 | -2.163778738 | 0.000130787 |
| LOC6053709 | protein Fe65 homolog | -1.405747765 | 0.000132491 |
| LOC6032998 | cuticle protein | 8.196682971 | 0.000132664 |
| LOC6034907 | uncharacterized LOC6034907 | 1.552379364 | 0.000139297 |
| LOC6040845 | 40S ribosomal protein S3a | 1.034310128 | 0.000140425 |
| LOC6040241 | putative mediator of RNA polymerase II transcription subunit 29 | -6.312866254 | 0.000141235 |
| LOC6034671 | uncharacterized LOC6034671 | -1.011936247 | 0.000141441 |
| LOC6037587 | bifunctional 3'-phosphoadenosine 5'-phosphosulfate synthase | -1.344228215 | 0.000141441 |
| LOC6039903 | general odorant-binding protein 19d | -8.219272635 | 0.000146154 |
| LOC6047962 | ribosomal protein 63, mitochondrial | 1.225949361 | 0.000149435 |
| LOC6046725 | cuticle protein 7 | 1.839341114 | 0.000149978 |
| LOC6051868 | uncharacterized LOC6051868 | 1.188296052 | 0.000149978 |
| LOC6048278 | protein crossbronx homolog | 1.412127195 | 0.000155561 |
| LOC6050687 | general odorant-binding protein 19d | 1.768252086 | 0.000156355 |
| LOC6036013 | blastoderm-specific protein 25D | -1.292863745 | 0.000158109 |
| LOC6034380 | ejaculatory bulb-specific protein 3 | 1.633382964 | 0.000158471 |
| LOC6053989 | vanin-like protein 2 | -1.144555808 | 0.00015936 |
| LOC6046994 | uncharacterized LOC6046994 | 1.826481862 | 0.000161093 |
| LOC119767674 | uncharacterized LOC119767674 | 4.503165916 | 0.000167902 |
| LOC6039312 | polyadenylate-binding protein | -3.864651857 | 0.000167902 |
| LOC119766567 | cathepsin B-like | -8.403638612 | 0.000169815 |
| LOC6034807 | nucleolar protein 56 | 1.0157125 | 0.000170705 |
| LOC6043253 | E3 ubiquitin-protein ligase TRIM37 | -2.695962195 | 0.000172585 |
| LOC6045187 | uncharacterized LOC6045187 | -2.650917196 | 0.000172585 |
| LOC6054154 | uncharacterized LOC6054154 | -5.295069899 | 0.000173685 |
| LOC6034552 | polyadenylate-binding protein, cytoplasmic and nuclear | -7.934345504 | 0.00017445 |
| LOC6046262 | microtubule-associated protein futsch | 1.530213863 | 0.000176255 |
| LOC119770816 | uncharacterized LOC119770816 | -7.954096301 | 0.000176307 |
| LOC119771131 | uncharacterized LOC119771131 | -4.299537775 | 0.000177106 |
| LOC6041016 | mRNA turnover protein 4 homolog | 1.699114122 | 0.000180196 |
| LOC6050945 | cytosolic non-specific dipeptidase | -1.157941352 | 0.000189535 |
| LOC6040958 | muscle-specific protein 20 | 1.420005584 | 0.000190818 |
| LOC6044349 | 28S ribosomal protein S29, mitochondrial | 1.130168017 | 0.000193811 |
| LOC6035810 | larval cuticle protein A2B | 2.286962616 | 0.000195965 |
| LOC6035871 | stAR-related lipid transfer protein 7, mitochondrial | -1.236667036 | 0.000196119 |
| LOC6031545 | cathepsin B | -7.872982949 | 0.000196727 |
| LOC119769756 | uncharacterized LOC119769756 | -8.007106847 | 0.000201338 |
| LOC6041748 | 40S ribosomal protein S24 | 1.092744565 | 0.000204766 |
| LOC6034682 | uncharacterized LOC6034682 | 2.132796359 | 0.000221478 |
| LOC6034603 | coatomer subunit zeta-1 | 1.018396697 | 0.000225146 |
| LOC6051345 | histone-lysine N-methyltransferase NSD2 | 4.660764962 | 0.000228679 |
| LOC6040255 | uncharacterized LOC6040255 | 3.749461101 | 0.000231548 |
| LOC6034620 | adult-specific cuticular protein ACP-20 | 8.529914388 | 0.000232461 |
| LOC6034862 | adult cuticle protein 1 | 4.390083599 | 0.000242299 |
| LOC119770380 | uncharacterized LOC119770380 | 8.02162507 | 0.000242459 |
| LOC6036595 | collagenase | 4.216951736 | 0.00024808 |
| LOC6036735 | bifunctional methylenetetrahydrofolate dehydrogenase/cyclohydrolase, mitochondrial | 1.283269279 | 0.000249084 |
| LOC6046726 | cuticle protein 7 | 8.031343837 | 0.00024953 |
| LOC119766315 | AT-rich interactive domain-containing protein 2-like | 2.407250319 | 0.000252764 |
| LOC6034000 | iron-sulfur cluster assembly 1 homolog, mitochondrial | 1.233499864 | 0.000252764 |
| LOC6043205 | E3 UFM1-protein ligase 1 homolog | -1.0364015 | 0.000253092 |
| LOC119765022 | uncharacterized LOC119765022 | 2.335168655 | 0.000257142 |
| LOC6049910 | very long-chain specific acyl-CoA dehydrogenase, mitochondrial | -1.40808089 | 0.000257355 |
| LOC119765538 | uncharacterized LOC119765538 | 6.869378143 | 0.000259644 |
| LOC6035655 | coiled-coil domain-containing protein 43 | 1.043086942 | 0.000259644 |
| LOC6031819 | uncharacterized LOC6031819 | 7.986475953 | 0.000261833 |
| LOC6036403 | 60S ribosomal protein L22 | 1.04925863 | 0.000261833 |
| LOC6034667 | cuticle protein LPCP-23 | 6.985386761 | 0.00026443 |
| LOC6052036 | odorant receptor 7a | 5.903643632 | 0.000265046 |
| LOC6038350 | arylsulfatase B | -1.50334727 | 0.000270191 |
| LOC6042707 | complex I intermediate-associated protein 30, mitochondrial | 1.200599815 | 0.000270191 |
| LOC6045141 | regulator of nonsense transcripts 1 homolog | -1.001374498 | 0.000274361 |
| LOC6045174 | uncharacterized LOC6045174 | -1.056256948 | 0.000278408 |
| LOC6033456 | folliculin-interacting protein 1 | -1.554189464 | 0.000281288 |
| LOC6038067 | uncharacterized LOC6038067 | 1.199381401 | 0.000282655 |
| LOC6045004 | uncharacterized LOC6045004 | 1.270747861 | 0.000282655 |
| LOC6047090 | 40S ribosomal protein S18 | 1.03200574 | 0.000289397 |
| LOC119767550 | peptidyl-prolyl cis-trans isomerase Fkbp12 | 1.123429786 | 0.00029025 |
| LOC6031537 | uncharacterized LOC6031537 | 1.619261601 | 0.00029025 |
| LOC6052898 | transferrin | -8.283445968 | 0.000292967 |
| LOC6048573 | 60S ribosomal protein L27a | 1.047466245 | 0.000294416 |
| LOC6039705 | GON-4-like protein | -1.455327718 | 0.000298553 |
| LOC6053908 | glutathione S-transferase theta-1 | -1.268150537 | 0.000302678 |
| LOC119765640 | uncharacterized LOC119765640 | 8.087368528 | 0.000302836 |
| LOC6045810 | leucine-rich repeat transmembrane neuronal protein 3 | 2.085470385 | 0.00030496 |
| LOC6031993 | juvenile hormone epoxide hydrolase 1 | -1.458365056 | 0.000306631 |
| LOC6033612 | cytidine deaminase | 1.217078859 | 0.000307753 |
| LOC119765099 | uncharacterized LOC119765099 | -1.655849271 | 0.000310645 |
| LOC6037168 | uncharacterized LOC6037168 | 3.001633079 | 0.000310645 |
| LOC6045405 | zinc finger protein 761 | 5.57138207 | 0.000311226 |
| LOC6047705 | ferritin subunit | 1.225859526 | 0.000313637 |
| LOC6048717 | uncharacterized LOC6048717 | -1.284034337 | 0.000317509 |
| LOC6034548 | serine/threonine-protein kinase PITSLRE | -1.028863601 | 0.000320315 |
| LOC119767176 | uncharacterized LOC119767176 | 8.123894077 | 0.000322372 |
| LOC6042314 | sphingosine kinase 2 | -1.469421232 | 0.000326731 |
| LOC6035221 | zinc finger protein OZF | 1.670936236 | 0.000333279 |
| LOC6048705 | something about silencing protein 10 | 1.744418803 | 0.000333279 |
| LOC6041781 | alpha,alpha-trehalose-phosphate synthase [UDP-forming] | -1.749734654 | 0.000336938 |
| LOC119765370 | uncharacterized LOC119765370 | -8.327112647 | 0.000345111 |
| LOC119765172 | uncharacterized LOC119765172 | 6.105110667 | 0.000346631 |
| LOC6038178 | protein stoned-A | -2.447275914 | 0.000356593 |
| LOC6042431 | GDP-fucose transporter 1 | 1.688442346 | 0.000356593 |
| LOC6032038 | phospholipase A1 member A | -6.009273168 | 0.000356838 |
| LOC6035071 | uncharacterized LOC6035071 | -2.368704803 | 0.000358686 |
| LOC6032897 | multiple epidermal growth factor-like domains protein 6 | 2.58258075 | 0.000358897 |
| LOC6041320 | CLIP domain-containing serine protease B15 | 1.606606349 | 0.000362958 |
| LOC6036732 | G2/mitotic-specific cyclin-B3 | -7.697393552 | 0.000364139 |
| LOC6053632 | protein disulfide-isomerase A5 | -1.172998208 | 0.000365544 |
| LOC119770843 | uncharacterized LOC119770843 | -8.113737329 | 0.000388321 |
| LOC6040384 | protein preli-like | 1.547359606 | 0.000388321 |
| LOC119767118 | uncharacterized LOC119767118 | -2.842474466 | 0.000400231 |
| LOC6038962 | SET and MYND domain-containing protein 5 | 1.894255296 | 0.000409579 |
| LOC6034604 | guanine nucleotide-binding protein G(f) subunit alpha | 2.330599087 | 0.000410996 |
| LOC6048531 | protein suppressor 2 of zeste | -1.728250735 | 0.000415849 |
| LOC6046214 | 40S ribosomal protein S24 | 1.034859444 | 0.00043559 |
| LOC6042565 | bromodomain adjacent to zinc finger domain protein 1A | -1.027490791 | 0.000439603 |
| LOC6038557 | uncharacterized LOC6038557 | 1.105763781 | 0.000439756 |
| LOC6041156 | lon protease homolog, mitochondrial | -1.45978567 | 0.000443955 |
| LOC119770455 | nose resistant to fluoxetine protein 6 | -1.832342675 | 0.000447921 |
| LOC6051562 | 39S ribosomal protein L12, mitochondrial | 1.121882152 | 0.000447921 |
| LOC6032550 | uncharacterized protein C11D3.03c | 1.680758652 | 0.000464644 |
| LOC6037418 | nucleolar protein dao-5 | -1.286563914 | 0.000465165 |
| LOC6042425 | uncharacterized LOC6042425 | -7.833257197 | 0.000465893 |
| LOC6039503 | uncharacterized LOC6039503 | -1.353265708 | 0.000469485 |
| LOC6039219 | myb-binding protein 1A | 1.442169887 | 0.000478502 |
| LOC6031176 | UDP-glycosyltransferase UGT5 | 1.453944851 | 0.000484979 |
| LOC6037612 | uncharacterized LOC6037612 | 1.448789236 | 0.000490866 |
| LOC6040663 | 39S ribosomal protein L55, mitochondrial | 1.464359416 | 0.000490866 |
| LOC6036767 | uncharacterized LOC6036767 | 1.234988511 | 0.000492695 |
| LOC6037159 | leucine-rich repeat-containing protein 26 | 1.064149127 | 0.000495823 |
| LOC6046814 | methanethiol oxidase | -1.031055927 | 0.000510651 |
| LOC6035388 | prostatic acid phosphatase | -1.085413054 | 0.000522317 |
| LOC6031210 | ribosome-releasing factor 2, mitochondrial | 1.424631553 | 0.000525074 |
| LOC6043997 | protein ILRUN | 1.002012749 | 0.000525074 |
| LOC6042727 | cytoplasmic dynein 1 light intermediate chain 2 | -1.056793981 | 0.000525378 |
| LOC6034843 | adult cuticle protein 1 | 3.80275397 | 0.000530038 |
| LOC119765659 | uncharacterized LOC119765659 | -7.549387289 | 0.00053275 |
| LOC6043123 | 60S ribosomal protein L31 | 1.043368428 | 0.00053275 |
| LOC6050403 | ubiquitin carboxyl-terminal hydrolase 20 | -1.25459266 | 0.00053275 |
| LOC6039905 | probable phosphoserine aminotransferase | 1.252875026 | 0.000536741 |
| LOC6039901 | probable multidrug resistance-associated protein lethal(2)03659 | 1.027517972 | 0.000539036 |
| LOC6040830 | UDP-glucosyltransferase 2 | -1.467086003 | 0.000542863 |
| LOC6049993 | gustatory receptor for sugar taste 64b | 4.224491357 | 0.000547297 |
| LOC6046891 | SH2B adapter protein 2 | -1.265196921 | 0.000555861 |
| LOC119765347 | uncharacterized LOC119765347 | 7.837093702 | 0.000556418 |
| LOC6050932 | tumor necrosis factor receptor superfamily member wengen | 1.253381886 | 0.000556418 |
| LOC6039508 | uncharacterized LOC6039508 | 2.570111087 | 0.000556961 |
| LOC6042005 | nuclear pore membrane glycoprotein 210 | -1.677564643 | 0.000563248 |
| LOC6044995 | ATP synthase subunit delta, mitochondrial | 1.02622729 | 0.000563248 |
| LOC6033231 | uncharacterized LOC6033231 | 1.574722239 | 0.000565739 |
| LOC6031135 | leukocyte elastase inhibitor | 4.362648967 | 0.000568749 |
| LOC119766608 | uncharacterized LOC119766608 | 1.491752007 | 0.000570184 |
| LOC119768639 | uncharacterized LOC119768639 | -7.625040059 | 0.000570184 |
| LOC6050355 | uncharacterized LOC6050355 | -3.555093528 | 0.000570184 |
| LOC6050854 | la protein homolog | 1.402996252 | 0.000570184 |
| LOC6046892 | DDB1- and CUL4-associated factor 12 | -1.850876794 | 0.000575501 |
| LOC6038903 | protein l(2)37Cc | 1.080582174 | 0.000580423 |
| LOC6042554 | vacuolar protein sorting-associated protein 37A | -1.391015266 | 0.000580423 |
| LOC6033982 | uncharacterized LOC6033982 | 1.414719908 | 0.000581539 |
| LOC6035072 | mitochondrial pyruvate carrier 1 | 1.433264615 | 0.000592756 |
| LOC119768767 | uncharacterized LOC119768767 | 6.09350874 | 0.00059421 |
| LOC119769715 | uncharacterized LOC119769715 | 2.796961572 | 0.00059421 |
| LOC6046560 | zinc finger protein 665 | -1.316416503 | 0.000594995 |
| LOC119770659 | uncharacterized LOC119770659 | -7.481605471 | 0.000598318 |
| LOC6053355 | cryptochrome-1 | -1.31253339 | 0.00060307 |
| LOC6036306 | F-box/LRR-repeat protein 6 | -1.344963527 | 0.000604963 |
| LOC6050354 | phospholipase B1, membrane-associated | -7.478395764 | 0.000614929 |
| LOC6049242 | 28S ribosomal protein S18a, mitochondrial | 1.171727468 | 0.000615441 |
| LOC6043488 | uncharacterized LOC6043488 | -1.799016576 | 0.000617205 |
| LOC6037447 | beta-1-syntrophin | -1.581753806 | 0.000618006 |
| LOC6048515 | U6 snRNA-associated Sm-like protein LSm6 | 1.386330866 | 0.000618006 |
| LOC6042911 | histone H2A-beta, sperm | 3.465895849 | 0.000621184 |
| LOC119766691 | uncharacterized LOC119766691 | -1.882703217 | 0.000622273 |
| LOC6032626 | GIGYF family protein CG11148 | -1.169671468 | 0.000624105 |
| LOC6035866 | ATP synthase membrane subunit DAPIT, mitochondrial | 1.236403543 | 0.000624647 |
| LOC6035435 | transmembrane protease serine 9 | -1.123345531 | 0.000632257 |
| LOC6042279 | uncharacterized LOC6042279 | -1.135322178 | 0.000633715 |
| LOC6041071 | coiled-coil domain-containing protein 170 | -7.793299703 | 0.000636632 |
| LOC6030809 | UDP-glucosyltransferase 2 | -2.440946425 | 0.000641755 |
| LOC6047885 | nucleolar protein 58 | 1.309555405 | 0.000648064 |
| LOC6032080 | homeobox protein Hox-A3a | -1.218480541 | 0.000660906 |
| LOC6037700 | pantothenate kinase 3 | 1.208635929 | 0.000661003 |
| LOC6049624 | probable cytochrome P450 313a4 | 1.909776568 | 0.000661003 |
| LOC6047012 | rhodanese domain-containing protein CG4456 | 3.399303093 | 0.00066136 |
| LOC6051359 | uncharacterized LOC6051359 | 1.068629877 | 0.00067576 |
| LOC6042757 | uncharacterized LOC6042757 | 2.58702563 | 0.000676089 |
| LOC6036156 | laminin subunit alpha-1 | -1.178732143 | 0.000678789 |
| LOC6031521 | uncharacterized LOC6031521 | 1.520430515 | 0.000693485 |
| LOC6031284 | uncharacterized LOC6031284 | 8.313339627 | 0.000694755 |
| LOC6031393 | uncharacterized LOC6031393 | -2.28640806 | 0.00070059 |
| LOC119770548 | mucin-5AC-like | -4.405012997 | 0.000718803 |
| LOC6039902 | multidrug resistance-associated protein 4 | 7.636725607 | 0.000718803 |
| LOC6048936 | uncharacterized LOC6048936 | -6.398385004 | 0.000720926 |
| LOC6034765 | serine/threonine-protein kinase MARK2 | -1.320491723 | 0.00072435 |
| LOC6046598 | esterase B1 | 1.285544944 | 0.000729529 |
| LOC6041515 | gustatory and pheromone receptor 39a | 4.043842569 | 0.000734799 |
| LOC6047732 | 40S ribosomal protein S25 | 1.027646837 | 0.000737922 |
| LOC6039313 | nuclear pore complex protein Nup58 | -1.106323588 | 0.000739906 |
| LOC6049413 | uncharacterized LOC6049413 | -4.290930999 | 0.00074433 |
| LOC6030792 | antigen 5 like allergen Cul n 1 | 7.600287823 | 0.000763365 |
| LOC6044611 | inorganic pyrophosphatase | 1.005500983 | 0.00077801 |
| LOC119769207 | uncharacterized LOC119769207 | -7.383612841 | 0.000780138 |
| LOC6053943 | uncharacterized LOC6053943 | 7.635104382 | 0.000782291 |
| LOC6033929 | uncharacterized LOC6033929 | 3.37734834 | 0.000794387 |
| LOC6033321 | transport and Golgi organization protein 6 | -1.575212803 | 0.000796113 |
| LOC6033387 | uncharacterized LOC6033387 | 1.585323469 | 0.000799416 |
| LOC6053346 | rutC family protein UK114 | 1.347129096 | 0.000824045 |
| LOC6039896 | huntingtin-interacting protein K | 1.200077963 | 0.000825696 |
| LOC6038508 | thymic stromal cotransporter homolog | 3.156702354 | 0.000825853 |
| LOC119770341 | pre-mRNA polyadenylation factor FIP1-like | -7.701067873 | 0.000832755 |
| LOC6052629 | uncharacterized LOC6052629 | -7.429948012 | 0.000840187 |
| LOC6054263 | cyclin-Y-like protein 1 | -1.661561406 | 0.000856311 |
| LOC119765259 | mitochondrial transcription rescue factor 1 | 1.472984972 | 0.000858477 |
| LOC6037064 | COP9 signalosome complex subunit 8 | 1.256100314 | 0.000871927 |
| LOC6031074 | protein ECT2 | -1.376090425 | 0.000874346 |
| LOC6032199 | nucleolar protein 8 | 1.491978854 | 0.000874346 |
| LOC119770223 | 60S ribosomal protein L30 | 1.052810613 | 0.000883438 |
| LOC6042776 | 26S proteasome complex subunit SEM1 | 1.087478131 | 0.000889084 |
| LOC119769664 | uncharacterized LOC119769664 | 5.644849775 | 0.000893141 |
| LOC119769768 | uncharacterized LOC119769768 | 1.737291388 | 0.000902022 |
| LOC6053318 | ionotropic receptor 25a | 1.402826548 | 0.000902022 |
| LOC6032392 | uncharacterized LOC6032392 | 1.010468384 | 0.000904073 |
| LOC6053386 | lambda-crystallin homolog | -1.67362576 | 0.000904073 |
| LOC6034425 | exosome complex component CSL4 | 1.448809061 | 0.000913367 |
| LOC6045247 | uncharacterized LOC6045247 | -1.471737388 | 0.000924072 |
| LOC6032134 | 40S ribosomal protein S21 | 1.193584156 | 0.000927584 |
| LOC6042825 | transcription elongation factor SPT4 | 1.100600832 | 0.000929244 |
| LOC6051379 | uncharacterized LOC6051379 | 1.885174385 | 0.000929244 |
| LOC119766089 | uncharacterized LOC119766089 | 2.03996658 | 0.000929305 |
| LOC119770534 | uncharacterized LOC119770534 | 3.581999689 | 0.000933765 |
| LOC6041324 | ras-related protein Rab-9B | -1.064570597 | 0.000940286 |
| LOC6047377 | D-glucuronyl C5-epimerase B | -2.343518773 | 0.000940969 |
| LOC6038592 | cell wall protein DAN4 | -1.686940773 | 0.00094174 |
| LOC6049584 | uncharacterized LOC6049584 | 1.122715322 | 0.00094174 |
| LOC119770032 | uncharacterized LOC119770032 | -7.348522315 | 0.000944951 |
| LOC6040414 | uncharacterized LOC6040414 | -1.229559043 | 0.000944951 |
| LOC6050016 | uncharacterized LOC6050016 | 7.594179727 | 0.000957174 |
| LOC6034861 | adult cuticle protein 1 | 4.398381047 | 0.000975152 |
| LOC6049143 | luciferin sulfotransferase | -1.609830103 | 0.000997692 |
| LOC119768075 | uncharacterized LOC119768075 | 1.912289424 | 0.001000891 |
| LOC6037693 | transmembrane channel-like protein 7 | -1.773680911 | 0.0010118 |
| LOC6042670 | 39S ribosomal protein L20, mitochondrial | 1.585786165 | 0.001016871 |
| LOC6045679 | syndetin | -1.177410316 | 0.001018277 |
| LOC6035694 | ORM1-like protein | 1.112830564 | 0.001021804 |
| LOC6052594 | protein wings apart-like | -1.038681199 | 0.001024868 |
| LOC6038948 | uncharacterized LOC6038948 | 1.082288401 | 0.001033173 |
| LOC119765371 | uncharacterized LOC119765371 | -4.748201624 | 0.001046689 |
| LOC6041862 | rRNA methyltransferase 3, mitochondrial | 1.667422974 | 0.001048849 |
| LOC6036842 | adenylate cyclase type 2 | -1.339831598 | 0.001060362 |
| LOC6041941 | AP-2 complex subunit sigma | 1.065870602 | 0.001060362 |
| LOC6049217 | splicing factor 3A subunit 1 | -1.011849447 | 0.00106381 |
| LOC6035865 | putative fatty acyl-CoA reductase CG5065 | -7.519369259 | 0.001090847 |
| LOC6040520 | aminopeptidase N | 1.609275431 | 0.001090847 |
| LOC6041984 | tRNA (guanine(37)-N1)-methyltransferase | 1.031620006 | 0.001099521 |
| LOC6037047 | putative ferric-chelate reductase 1 homolog | 2.902407713 | 0.001108547 |
| LOC119769129 | uncharacterized LOC119769129 | -7.237472227 | 0.001112037 |
| LOC6035667 | uncharacterized LOC6035667 | -1.675271743 | 0.001126172 |
| LOC119768862 | uncharacterized LOC119768862 | -3.955676145 | 0.001127236 |
| LOC6036517 | cadherin-99C | -1.417285304 | 0.001129046 |
| LOC119765112 | uncharacterized LOC119765112 | 1.269231285 | 0.001138693 |
| LOC6043215 | zinc finger protein 37 homolog | -1.380642068 | 0.001146651 |
| LOC6050642 | uncharacterized LOC6050642 | 1.492094105 | 0.00114964 |
| LOC6043440 | uncharacterized LOC6043440 | 6.168157237 | 0.001149981 |
| LOC6046342 | sorbitol dehydrogenase | -2.204416473 | 0.001150053 |
| LOC6040686 | uncharacterized LOC6040686 | 1.071546453 | 0.00115267 |
| LOC6036919 | 40S ribosomal protein S2 | 8.055624104 | 0.001155797 |
| LOC6046054 | E3 ubiquitin-protein ligase TRIP12 | -1.103359075 | 0.001156748 |
| LOC119767793 | uncharacterized LOC119767793 | 7.595906571 | 0.001177101 |
| LOC6043200 | 60S acidic ribosomal protein P1 | 1.034785155 | 0.001177101 |
| LOC6045093 | uncharacterized LOC6045093 | 2.445757879 | 0.001181808 |
| LOC6040781 | E3 ubiquitin-protein ligase RBBP6 | -1.055882228 | 0.001190732 |
| LOC6051904 | mitochondrial import inner membrane translocase subunit Tim8 | 1.121957732 | 0.001214336 |
| LOC119770365 | epoxide hydrolase 4-like | -1.882810942 | 0.001217371 |
| LOC6047234 | uncharacterized LOC6047234 | 1.429944698 | 0.001224808 |
| LOC6041431 | uncharacterized LOC6041431 | -7.244331609 | 0.001233482 |
| LOC6048237 | meiotic nuclear division protein 1 homolog | -3.380599558 | 0.001233482 |
| LOC6049626 | protein HGH1 homolog | 1.370444663 | 0.001245877 |
| LOC6034696 | zinc finger protein 266 | -2.247867567 | 0.001256943 |
| LOC6050543 | V-type proton ATPase 116 kDa subunit a1 | 1.586456542 | 0.001256943 |
| LOC119765960 | uncharacterized LOC119765960 | -4.494911544 | 0.001277848 |
| LOC6033135 | restin homolog | 1.039434344 | 0.001281374 |
| LOC6040158 | GATA-binding factor A | -7.554382889 | 0.001307261 |
| LOC6049700 | uncharacterized LOC6049700 | 1.607128748 | 0.001315973 |
| LOC6037283 | phospholipid scramblase 2 | 1.946799596 | 0.001316818 |
| LOC6046385 | uncharacterized LOC6046385 | 1.114783021 | 0.001319489 |
| LOC119766069 | uncharacterized LOC119766069 | 2.50262927 | 0.001332085 |
| LOC119768576 | 40S ribosomal protein S24-like | 2.280888122 | 0.001348335 |
| LOC6036459 | SNAPIN protein homolog | 1.001528447 | 0.001355593 |
| LOC6039202 | lysM and putative peptidoglycan-binding domain-containing protein 2 | -7.180312469 | 0.001364146 |
| LOC6032331 | uncharacterized LOC6032331 | 1.666363775 | 0.001371765 |
| LOC6041005 | lactase-like protein | -7.54615749 | 0.001398397 |
| LOC6032330 | dihydroceramide fatty acyl 2-hydroxylase FAH2 | -1.705276535 | 0.001455352 |
| LOC6032629 | uncharacterized LOC6032629 | -1.02874455 | 0.001475427 |
| LOC6051969 | beta-1,4-glucuronyltransferase 1 | -1.433208612 | 0.001506338 |
| LOC6034882 | protein fuzzy homolog | 1.482498369 | 0.001621908 |
| LOC119769039 | uncharacterized LOC119769039 | -7.152716849 | 0.001651084 |
| LOC6037197 | U11/U12 small nuclear ribonucleoprotein 35 kDa protein | -7.187568154 | 0.001657683 |
| LOC119769695 | uncharacterized LOC119769695 | 7.273718893 | 0.001684576 |
| LOC6035433 | serine protease 7 | -1.224131271 | 0.001691405 |
| LOC6044999 | MICOS complex subunit Mic10 | 1.266575796 | 0.00173669 |
| LOC6048328 | uncharacterized LOC6048328 | 1.287842551 | 0.001752365 |
| LOC6031308 | rutC family protein UK114 | 1.127087465 | 0.001761172 |
| LOC6037266 | protein lethal(2)essential for life | 3.812237143 | 0.001763549 |
| LOC6053615 | D-beta-hydroxybutyrate dehydrogenase, mitochondrial | 1.013989167 | 0.001764968 |
| LOC119769842 | uncharacterized LOC119769842 | 2.465253827 | 0.00178312 |
| LOC6050833 | uncharacterized LOC6050833 | 6.33848377 | 0.001805976 |
| LOC6040499 | 40S ribosomal protein S12, mitochondrial | 1.218223978 | 0.001808689 |
| LOC6045070 | RNA polymerase II degradation factor 1 | 3.046253585 | 0.001852859 |
| LOC119765400 | myb-like protein AA | -7.060736223 | 0.001856989 |
| LOC119769612 | cytochrome c oxidase subunit 7C, mitochondrial-like | 1.029052945 | 0.001871706 |
| LOC6046917 | frataxin homolog, mitochondrial | 1.120088004 | 0.001874271 |
| LOC6048148 | protein sarah | -1.643854543 | 0.001890816 |
| LOC6033631 | rRNA-processing protein FCF1 homolog | 1.515158168 | 0.00190034 |
| LOC119765168 | transmembrane protein 17-like | 2.535708825 | 0.001902267 |
| LOC6048737 | programmed cell death protein 2 | 1.426391556 | 0.001932396 |
| LOC6032328 | eukaryotic translation initiation factor 5A | 1.069072203 | 0.001955369 |
| LOC6045826 | thrombospondin-4 | 1.30531019 | 0.001977487 |
| LOC6040082 | pleckstrin homology domain-containing family G member 5 | -1.585453067 | 0.001978367 |
| LOC6038882 | 18S rRNA aminocarboxypropyltransferase | 1.382356365 | 0.001981277 |
| LOC119770818 | uncharacterized LOC119770818 | -2.499773363 | 0.002002593 |
| LOC6040622 | peroxisomal N(1)-acetyl-spermine/spermidine oxidase | 1.480779508 | 0.002019776 |
| LOC6050834 | pupal cuticle protein | 3.65237061 | 0.002036611 |
| LOC6051803 | clotting factor B | 3.301493359 | 0.002053176 |
| LOC6045918 | serine/threonine-protein kinase WNK1 | -1.046304904 | 0.002055605 |
| LOC119766640 | uncharacterized LOC119766640 | -7.00049412 | 0.00206319 |
| LOC6031325 | cytochrome c oxidase subunit 6C | 1.070683886 | 0.00206319 |
| LOC6050720 | probable cytochrome P450 6a20 | 1.275344932 | 0.002129505 |
| LOC119768024 | uncharacterized LOC119768024 | -2.446673197 | 0.002133031 |
| LOC6044213 | mitochondrial amidoxime-reducing component 1 | -1.205878035 | 0.002174547 |
| LOC6044659 | natterin-1 | 4.303558829 | 0.002188621 |
| LOC6050484 | glutamate-rich WD repeat-containing protein 1 | 1.277849921 | 0.002200336 |
| LOC6040700 | alkyldihydroxyacetonephosphate synthase | -1.282580977 | 0.002200418 |
| LOC6031531 | tetracycline resistance protein, class D | 2.52941035 | 0.002226196 |
| LOC6049318 | uncharacterized LOC6049318 | 3.347997461 | 0.002235845 |
| LOC119770411 | uncharacterized LOC119770411 | 7.193313078 | 0.002249649 |
| LOC6046856 | WD repeat-containing protein 48 homolog | -1.00088276 | 0.002249649 |
| LOC6043177 | testisin | 4.023053237 | 0.002264117 |
| LOC6033964 | tetratricopeptide repeat protein 30 homolog | -1.815918005 | 0.002282937 |
| LOC6050685 | uncharacterized LOC6050685 | -4.967670446 | 0.00229515 |
| LOC119770603 | uncharacterized LOC119770603 | 3.463437502 | 0.002305572 |
| LOC6032382 | zinc finger HIT domain-containing protein 3 | 1.969048724 | 0.002316184 |
| LOC6041278 | putative sulfiredoxin | 1.692297434 | 0.002320415 |
| LOC6041994 | 39S ribosomal protein L36, mitochondrial | 1.145139812 | 0.00233776 |
| LOC6041015 | mucin-5AC | 1.185795468 | 0.002343854 |
| LOC6031511 | coiled-coil domain-containing protein 86 | 1.766981982 | 0.002371114 |
| LOC6053332 | ATP-dependent RNA helicase DDX42 | -1.040193586 | 0.002371114 |
| LOC6049110 | enkurin | 4.231257297 | 0.002376589 |
| LOC6038124 | cytochrome b-c1 complex subunit 10 | 1.036587842 | 0.002401745 |
| LOC119769400 | uncharacterized LOC119769400 | -7.088097546 | 0.002429013 |
| LOC6049137 | RNA-binding motif protein, X-linked 2 | 1.912509543 | 0.002429013 |
| LOC119765349 | uncharacterized LOC119765349 | 2.805202471 | 0.002448776 |
| LOC6048847 | serine/threonine-protein kinase PAK 2 | -1.018808605 | 0.002448928 |
| LOC6047324 | retinaldehyde-binding protein 1 | 2.953797842 | 0.002490136 |
| LOC6042387 | uncharacterized LOC6042387 | -6.927333835 | 0.002510749 |
| LOC6033216 | protein arginine N-methyltransferase 5 | 1.270602387 | 0.002518374 |
| LOC6045839 | phenoloxidase-activating factor 2 | 1.879225692 | 0.002521333 |
| LOC6036302 | probable multidrug resistance-associated protein lethal(2)03659 | -1.001809885 | 0.002552957 |
| LOC6030826 | malate synthase | -1.240521661 | 0.002583007 |
| LOC6038950 | maternal effect protein oskar | -2.901618313 | 0.002583007 |
| LOC6046626 | apolipoprotein D | 7.05441747 | 0.002591298 |
| LOC119769512 | uncharacterized LOC119769512 | 1.123757252 | 0.002601903 |
| LOC119770528 | THAP domain-containing protein 2-like | 4.480528293 | 0.002601903 |
| LOC6049099 | uncharacterized LOC6049099 | -1.643800119 | 0.002616914 |
| LOC6042527 | guanylate cyclase 32E | 1.28776359 | 0.002626352 |
| LOC6049529 | arginase, hepatic | 2.051716637 | 0.002642356 |
| LOC6041266 | adult-specific cuticular protein ACP-20 | 1.992290366 | 0.002653132 |
| LOC119765010 | 1,5-anhydro-D-fructose reductase-like | 1.467970116 | 0.002679833 |
| LOC6034159 | uncharacterized LOC6034159 | -1.359972881 | 0.002679833 |
| LOC6049623 | probable cytochrome P450 313a4 | 1.668633235 | 0.002700898 |
| LOC6045077 | uncharacterized LOC6045077 | -1.143869479 | 0.002712634 |
| LOC6036777 | sodium-independent sulfate anion transporter | 1.234493163 | 0.002715763 |
| LOC6036269 | nucleoside diphosphate kinase | 1.014241952 | 0.002743944 |
| LOC6039337 | proton-coupled folate transporter | 1.035296014 | 0.002758519 |
| LOC6054480 | late secretory pathway protein AVL9 homolog | -1.19937914 | 0.002764553 |
| LOC6035774 | engulfment and cell motility protein 1 | -1.086013276 | 0.002779592 |
| LOC6036119 | putative mediator of RNA polymerase II transcription subunit 26 | -1.226712459 | 0.00279352 |
| LOC6049154 | NADH dehydrogenase [ubiquinone] 1 beta subcomplex subunit 1 | 1.059294577 | 0.002797712 |
| LOC6036649 | uncharacterized LOC6036649 | -2.013508235 | 0.002798175 |
| LOC119767686 | uncharacterized serine-rich protein C215.13 | -2.364943716 | 0.002815462 |
| LOC6038728 | regucalcin | -2.208940634 | 0.002819334 |
| LOC6048513 | uncharacterized LOC6048513 | -1.241912076 | 0.002826663 |
| LOC6034471 | pyridoxal-dependent decarboxylase domain-containing protein 1 | -1.092886625 | 0.002831234 |
| LOC6033359 | canalicular multispecific organic anion transporter 1 | -5.198088147 | 0.002846702 |
| LOC119766739 | uncharacterized LOC119766739 | 3.835657798 | 0.002870692 |
| LOC6051538 | scavenger receptor class B member 1 | 1.586528621 | 0.00288858 |
| LOC6036411 | uncharacterized LOC6036411 | 1.30624991 | 0.002943963 |
| LOC6036904 | calpain-C | -1.43762245 | 0.002960521 |
| LOC119765491 | transcription factor grauzone-like | -1.644587012 | 0.002971706 |
| LOC6033224 | dedicator of cytokinesis protein 9 | -1.081034614 | 0.003009883 |
| LOC6039013 | protein disulfide-isomerase A6 homolog | -1.059069708 | 0.003018709 |
| LOC6032985 | hexamerin-1.1 | 1.652802143 | 0.00306781 |
| LOC119767575 | fibrinogen-like protein A | -1.414320779 | 0.003080209 |
| LOC6050133 | outer membrane lipoprotein Blc | -1.63124913 | 0.003080209 |
| LOC119767845 | uncharacterized LOC119767845 | 4.669534371 | 0.003090196 |
| LOC6046066 | RNA polymerase II-associated factor 1 homolog | -1.076464084 | 0.003092139 |
| LOC119767345 | uncharacterized LOC119767345 | 1.405092119 | 0.003106073 |
| LOC6051047 | solute carrier family 22 member 21 | -1.017810839 | 0.003106073 |
| LOC6053086 | sodium-dependent nutrient amino acid transporter 1 | -1.038409005 | 0.003110546 |
| LOC6047629 | protein virilizer | -1.129778239 | 0.003168735 |
| LOC6034937 | cadherin-87A | -1.351694386 | 0.003195999 |
| LOC6038893 | uncharacterized LOC6038893 | 3.212354156 | 0.003204889 |
| LOC119766929 | uncharacterized LOC119766929 | -2.498760615 | 0.003219114 |
| LOC6031014 | probable cytochrome P450 4ac1 | -1.339129099 | 0.003240022 |
| LOC6052418 | dnaJ homolog subfamily C member 22 | -1.550597172 | 0.003240022 |
| LOC6044293 | TBC1 domain family member whacked | -1.172141538 | 0.003264165 |
| LOC6045851 | leucine-rich repeat neuronal protein 2 | -6.740990851 | 0.003276683 |
| LOC6032284 | putative helicase MOV-10 | -2.91476307 | 0.00328537 |
| LOC6047298 | hydroxyacid oxidase 1 | -1.586121406 | 0.003301019 |
| LOC6036261 | probable chitinase 2 | -1.724376626 | 0.003321026 |
| LOC119770732 | glycine-rich cell wall structural protein 2-like | -1.375580095 | 0.003328051 |
| LOC6038313 | uncharacterized LOC6038313 | -6.72559744 | 0.003333356 |
| LOC6036379 | developmental protein eyes absent | -1.091629851 | 0.003336167 |
| LOC119770623 | uncharacterized LOC119770623 | -1.311379064 | 0.00338072 |
| LOC119767312 | uncharacterized LOC119767312 | 2.228972358 | 0.003386468 |
| LOC6043834 | general odorant-binding protein 99a | 1.541378723 | 0.003409215 |
| LOC6035141 | prostaglandin reductase 1 | 3.266271575 | 0.003472454 |
| LOC6040698 | odorant receptor 13a | -6.731941114 | 0.003472454 |
| LOC119770552 | uncharacterized LOC119770552 | -3.870087214 | 0.003505805 |
| LOC6042060 | general odorant-binding protein 84a | 1.567450701 | 0.003543101 |
| LOC6042393 | alpha-mannosidase 2 | -1.219844341 | 0.003597156 |
| LOC6045432 | actin-1 | 1.060369482 | 0.003597156 |
| LOC6030985 | UPF0489 protein C5orf22 homolog | -1.385149912 | 0.003643034 |
| LOC6036437 | cysteine-rich venom protein | 1.91859735 | 0.003648483 |
| LOC6036301 | farnesol dehydrogenase | 2.957349796 | 0.003681204 |
| LOC6037122 | 60S ribosomal protein L38 | 1.029985967 | 0.003681655 |
| LOC6033215 | U3 small nucleolar RNA-associated protein 18 homolog | 1.194392275 | 0.00368737 |
| LOC6035727 | odorant receptor 67a | 2.714576902 | 0.003699453 |
| LOC6041319 | CLIP domain-containing serine protease B15 | -3.63890733 | 0.003699453 |
| LOC6044256 | secretion-regulating guanine nucleotide exchange factor | 1.203752719 | 0.003699453 |
| LOC6048144 | 39S ribosomal protein L46, mitochondrial | 1.162017081 | 0.003722799 |
| LOC6041270 | glycerol-3-phosphate phosphatase | -1.169310523 | 0.003730297 |
| LOC6042051 | putative odorant receptor 85e | 3.66477254 | 0.003731174 |
| LOC6046881 | probable ATP-dependent RNA helicase DDX28 | 1.646256618 | 0.003737224 |
| LOC6046125 | ATP-binding cassette sub-family G member 4 | -1.128067292 | 0.003748251 |
| LOC119769408 | uncharacterized LOC119769408 | 2.525278197 | 0.003799947 |
| LOC119771155 | induced during hyphae development protein 1-like | -1.078483517 | 0.003799947 |
| LOC6032836 | general odorant-binding protein 66 | 1.426628237 | 0.003799947 |
| LOC6042419 | N-acetylglucosamine-6-phosphate deacetylase | -1.113434678 | 0.003799947 |
| LOC119769800 | uncharacterized LOC119769800 | -1.558458869 | 0.003807093 |
| LOC6039535 | uncharacterized LOC6039535 | 1.317432076 | 0.003834065 |
| LOC6035038 | beta-1,3-glucan-binding protein | -5.648900523 | 0.003858036 |
| LOC6033496 | uncharacterized LOC6033496 | -1.794065602 | 0.003877988 |
| LOC119768739 | uncharacterized LOC119768739 | 1.919579278 | 0.003890538 |
| LOC6035812 | larval cuticle protein A2B | 2.838370109 | 0.003905584 |
| LOC6044017 | protein FAM136A | 1.467731964 | 0.003921542 |
| LOC119765673 | general odorant-binding protein 45-like | 1.815704146 | 0.003936411 |
| LOC6033569 | uncharacterized LOC6033569 | 1.255573955 | 0.003942295 |
| LOC6035875 | phospholipase A1 | -2.236026243 | 0.004002931 |
| LOC6038946 | translation machinery-associated protein 16 homolog | 1.463676807 | 0.00401751 |
| LOC6045194 | ATP synthase subunit C lysine N-methyltransferase | 1.530663751 | 0.00401751 |
| LOC6037298 | surfeit locus protein 6 homolog | 1.178474079 | 0.004081208 |
| LOC119767362 | uncharacterized LOC119767362 | 1.660838836 | 0.00411314 |
| LOC6041227 | pre-mRNA-splicing factor 38 | -1.577029974 | 0.00411314 |
| LOC119770027 | shematrin-like protein 2 | 2.048838143 | 0.004116092 |
| LOC6052157 | toll-like receptor 6 | -1.934310914 | 0.004124542 |
| LOC6048831 | lectin subunit alpha | 2.435205622 | 0.004133947 |
| LOC6031256 | alpha-aminoadipic semialdehyde synthase, mitochondrial | 1.438228691 | 0.004172348 |
| LOC6032513 | polyhomeotic-proximal chromatin protein | -1.055633682 | 0.004184736 |
| LOC119765928 | uncharacterized LOC119765928 | 6.74991387 | 0.004246923 |
| LOC6054290 | probable basic-leucine zipper transcription factor I | 1.794488042 | 0.004255541 |
| LOC6045821 | cytosolic purine 5'-nucleotidase | 1.001694242 | 0.004263505 |
| LOC6053422 | mucin-2 | 1.862909793 | 0.004317657 |
| LOC6035115 | uncharacterized LOC6035115 | -1.491179473 | 0.004360064 |
| LOC6046348 | sorbitol dehydrogenase | -1.323961383 | 0.004379986 |
| LOC6034150 | DNA-directed RNA polymerases I and III subunit RPAC1 | 1.227440654 | 0.004392053 |
| LOC6037748 | zinc finger protein 845 | 1.250757853 | 0.004411097 |
| LOC6045171 | uncharacterized LOC6045171 | -1.22398815 | 0.004411097 |
| LOC6054371 | CLIP domain-containing serine protease 14D | 1.427057291 | 0.004429648 |
| LOC119770851 | UDP-glucosyltransferase 2-like | 1.088411966 | 0.004447606 |
| LOC119770064 | uncharacterized LOC119770064 | 4.305440984 | 0.004498305 |
| LOC6043376 | uncharacterized LOC6043376 | 1.294628465 | 0.00454592 |
| LOC6042322 | fas-associated death domain protein | 1.941442059 | 0.004557123 |
| LOC119770459 | uncharacterized LOC119770459 | 2.811309482 | 0.00459118 |
| LOC6044077 | uncharacterized LOC6044077 | 1.467012359 | 0.004636787 |
| LOC6049574 | uncharacterized LOC6049574 | -3.667415926 | 0.004726467 |
| LOC6047737 | histone-lysine N-methyltransferase trithorax | -1.11978504 | 0.004736242 |
| LOC6048707 | constitutive coactivator of peroxisome proliferator-activated receptor gamma | 1.375790116 | 0.004746258 |
| LOC6041553 | transmembrane and coiled-coil domains protein 1 | -1.008526497 | 0.004792542 |
| LOC6035068 | protein C3orf33 homolog | 1.052475429 | 0.004857748 |
| LOC6034233 | ecdysone receptor | -1.386986714 | 0.004870988 |
| LOC6048090 | uncharacterized LOC6048090 | -1.035742212 | 0.004953223 |
| LOC6034331 | corticotropin-releasing factor-binding protein | 2.079249853 | 0.004956731 |
| LOC6048117 | E3 ubiquitin-protein ligase RNF12-B | 5.189438818 | 0.004956731 |
| LOC6038312 | uncharacterized LOC6038312 | -1.060711113 | 0.005004006 |
| LOC6035085 | ecotropic viral integration site 5 ortholog | -1.419349366 | 0.005005274 |
| LOC6042107 | cuticle protein CP14.6 | 5.199136772 | 0.005027686 |
| LOC6036104 | protein chiffon | -1.061635753 | 0.005041597 |
| LOC6043301 | UPF0193 protein EVG1 homolog | 1.487157435 | 0.005079131 |
| LOC119767637 | uncharacterized LOC119767637 | 2.143924624 | 0.005149212 |
| LOC6052602 | Kv channel-interacting protein 1 | -1.599980548 | 0.005197887 |
| LOC6031703 | LRR receptor-like serine/threonine-protein kinase ERECTA | 1.745738327 | 0.005243605 |
| LOC119771142 | ras-interacting protein RIP3 | -1.191742236 | 0.005269302 |
| LOC6046147 | endochitinase | -1.268650137 | 0.005281104 |
| LOC119766981 | uncharacterized LOC119766981 | 1.255289793 | 0.005426914 |
| LOC6049002 | probable nuclear hormone receptor HR3 | -1.265229768 | 0.005448867 |
| LOC119765905 | uncharacterized LOC119765905 | 1.861145438 | 0.005468801 |
| LOC6031475 | zinc finger CCCH domain-containing protein 18 | -1.00369205 | 0.005545602 |
| LOC6046094 | uncharacterized LOC6046094 | 1.094210764 | 0.005553651 |
| LOC6049361 | spondin-2 | 1.005894674 | 0.00556508 |
| LOC119769415 | uncharacterized LOC119769415 | 1.607591111 | 0.005570658 |
| LOC6049604 | esterase B1 | -4.801270525 | 0.005573376 |
| LOC6052739 | uncharacterized LOC6052739 | 1.128176058 | 0.005607896 |
| LOC6036776 | 60S ribosome subunit biogenesis protein NIP7 homolog | 1.217441878 | 0.00563799 |
| LOC6034103 | zonadhesin | 1.907248705 | 0.005640778 |
| LOC6039250 | CLIP domain-containing serine protease 14D | 1.744437533 | 0.005651823 |
| LOC6051166 | uncharacterized LOC6051166 | 3.962442462 | 0.005659088 |
| LOC6046249 | uncharacterized LOC6046249 | 1.703123999 | 0.00574577 |
| LOC6045703 | uncharacterized LOC6045703 | -3.973018338 | 0.005810305 |
| LOC119765655 | uncharacterized LOC119765655 | -3.659091764 | 0.005858294 |
| LOC119767457 | uncharacterized LOC119767457 | 3.670436749 | 0.00594873 |
| LOC6036223 | tubulin alpha-8 chain | -4.061987664 | 0.005977271 |
| LOC6034665 | cuticle protein 16.5 | 1.826393345 | 0.005996046 |
| LOC6051499 | uncharacterized protein F54F2.9 | -1.155779085 | 0.006054772 |
| LOC6034700 | zinc finger protein 587 | -2.860903333 | 0.006090756 |
| LOC6052140 | fatty acyl-CoA reductase wat | -1.542080863 | 0.006094896 |
| LOC6037686 | neuralized-like protein 4 | -1.146669974 | 0.006101784 |
| LOC6052250 | homeobox protein 5 | -1.01151313 | 0.006112937 |
| LOC6044043 | lipase maturation factor 2 | -1.141518302 | 0.006183631 |
| LOC6051842 | periodic tryptophan protein 1 homolog | 1.362436595 | 0.006437978 |
| LOC119769570 | uncharacterized LOC119769570 | -4.55244866 | 0.006446946 |
| LOC6042010 | 40S ribosomal protein S29 | 1.076745761 | 0.006457515 |
| LOC6051380 | glycerol-3-phosphate phosphatase | -1.077813425 | 0.006463776 |
| LOC119769015 | uncharacterized LOC119769015 | 1.492997553 | 0.006466922 |
| LOC119770819 | uncharacterized LOC119770819 | 1.682158512 | 0.006476895 |
| LOC6031216 | uncharacterized LOC6031216 | -1.500226367 | 0.006476895 |
| LOC6034669 | ice-structuring glycoprotein | 3.122442164 | 0.006476895 |
| LOC6051861 | frizzled-2 | -1.181257464 | 0.006564066 |
| LOC6049808 | extracellular serine/threonine protein CG31145 | -1.379083543 | 0.00657746 |
| LOC119767430 | venom serine protease Bi-VSP-like | 2.4752946 | 0.006660201 |
| LOC6037221 | uncharacterized LOC6037221 | -1.595267454 | 0.006660201 |
| LOC6036623 | uncharacterized LOC6036623 | 1.374206109 | 0.006702076 |
| LOC6037470 | trafficking protein particle complex subunit 6b | 1.085501623 | 0.006704978 |
| LOC119768574 | uncharacterized LOC119768574 | -3.166358947 | 0.006717551 |
| LOC6042033 | general odorant-binding protein 72 | 1.291941943 | 0.00674409 |
| LOC6050573 | laccase-4 | 1.772593397 | 0.006817269 |
| LOC6034885 | uncharacterized LOC6034885 | 1.063462174 | 0.006895928 |
| LOC6034919 | tetratricopeptide repeat protein 28 | -1.138508827 | 0.006955996 |
| LOC6040729 | acrosin | -2.153535414 | 0.006986848 |
| LOC119766418 | homeodomain-interacting protein kinase 2-like | -2.91604189 | 0.007087506 |
| LOC119770348 | phosphoglycolate phosphatase 2-like | -1.54774533 | 0.007102406 |
| LOC6031817 | golgin subfamily A member 6-like protein 6 | 2.162556182 | 0.007129333 |
| LOC6048273 | probable 28S ribosomal protein S25, mitochondrial | 1.202073299 | 0.007188554 |
| LOC119769462 | uncharacterized LOC119769462 | -4.699395126 | 0.007206642 |
| LOC6052454 | uncharacterized LOC6052454 | -4.720134621 | 0.007345763 |
| LOC6031731 | uncharacterized LOC6031731 | 1.467622982 | 0.00734862 |
| LOC119769817 | SLIT and NTRK-like protein 6 | 3.178597524 | 0.007408449 |
| LOC119771186 | uncharacterized LOC119771186 | -1.479621732 | 0.007433913 |
| LOC6041500 | ATP-dependent RNA helicase vasa | -1.270485847 | 0.007448047 |
| LOC119767041 | uncharacterized LOC119767041 | 3.186164204 | 0.007588244 |
| LOC6043364 | glutathione S-transferase 4 | -1.220245151 | 0.007611724 |
| LOC6032938 | dynein assembly factor with WDR repeat domains 1 | -5.119514067 | 0.007637728 |
| LOC119770633 | H/ACA ribonucleoprotein complex subunit 1 | 1.471160581 | 0.007651884 |
| LOC6038505 | inhibitor of growth protein 5 | -1.002981005 | 0.007652061 |
| LOC6035507 | glutamyl-tRNA(Gln) amidotransferase subunit A, mitochondrial | 1.568182012 | 0.007667376 |
| LOC6046666 | KH homology domain-containing protein 4 | 3.84024368 | 0.007765931 |
| LOC6044092 | clavesin-1 | 1.064141367 | 0.007839139 |
| LOC6047231 | uncharacterized LOC6047231 | 1.228271017 | 0.007839139 |
| LOC6050265 | keratin, type I cytoskeletal 10 | 5.018288043 | 0.007839139 |
| LOC6031054 | ficolin-1 | 1.1541585 | 0.007903263 |
| LOC6049262 | 5-hydroxytryptamine receptor 1 | -1.489334128 | 0.007914528 |
| LOC6041052 | superoxide dismutase [Cu-Zn] | 1.08136135 | 0.007927348 |
| LOC6043801 | uncharacterized LOC6043801 | -3.799980086 | 0.007958172 |
| LOC119767467 | alkaline phosphatase 4 | 1.008243816 | 0.008195596 |
| LOC119768638 | histidine triad nucleotide-binding protein 1 | 1.130537525 | 0.008230328 |
| LOC6037543 | arrestin domain-containing protein 3 | -2.825833084 | 0.008264511 |
| LOC6048915 | transcription factor 25 | -1.075533461 | 0.008391561 |
| LOC6037263 | protein lethal(2)essential for life | 5.231488505 | 0.008419531 |
| LOC119767840 | amyloid protein-binding protein 2-like | -1.15141407 | 0.008481171 |
| LOC6036006 | zinc finger protein 808 | -1.08124855 | 0.00859726 |
| LOC6039928 | uncharacterized LOC6039928 | -1.112699075 | 0.008598384 |
| LOC6048136 | cuticle protein 21 | 2.146051643 | 0.00877527 |
| LOC6043183 | mucin-5AC | 2.088699671 | 0.008788079 |
| LOC6031303 | intraflagellar transport protein 172 homolog | -1.814288134 | 0.008927678 |
| LOC6043698 | solute carrier family 28 member 3 | -1.43589034 | 0.008971057 |
| LOC6031939 | structural maintenance of chromosomes protein 4 | -1.016929255 | 0.008978404 |
| LOC6047252 | xanthine dehydrogenase/oxidase | -1.294532074 | 0.009056496 |
| LOC119770208 | uncharacterized LOC119770208 | -1.234184957 | 0.009095187 |
| LOC6046259 | opsin-1 | -1.038137272 | 0.009098217 |
| LOC119766738 | uncharacterized LOC119766738 | 1.900713461 | 0.009139674 |
| LOC6031568 | DNA-dependent metalloprotease SPRTN | -1.107686488 | 0.009155936 |
| LOC119767677 | uncharacterized LOC119767677 | 2.167635036 | 0.009254 |
| LOC6050920 | protein bowel | -1.449185 | 0.009279906 |
| LOC6054427 | leucine-rich repeat-containing protein 40 | 2.813922393 | 0.009667168 |
| LOC6035348 | protein Spindly | 1.424633166 | 0.009970331 |
| LOC6052340 | fibrillin-1 | 1.320591051 | 0.010009425 |
| LOC6032966 | A disintegrin and metalloproteinase with thrombospondin motifs adt-2 | 4.578982345 | 0.010010222 |
| LOC6042708 | progestin and adipoQ receptor family member 4 | -1.344124511 | 0.010010222 |
| LOC6045882 | putative helicase mov-10-B.1 | -1.451417084 | 0.010010222 |
| LOC6046138 | uncharacterized LOC6046138 | -1.090692201 | 0.010010222 |
| LOC119769405 | uncharacterized LOC119769405 | 3.234036828 | 0.010079042 |
| LOC6049374 | uncharacterized LOC6049374 | -1.084593873 | 0.010093695 |
| LOC6035831 | ribosomal RNA small subunit methyltransferase NEP1 | 1.373765748 | 0.010193087 |
| LOC6044244 | uncharacterized LOC6044244 | 1.32260917 | 0.010238395 |
| LOC119767032 | uncharacterized LOC119767032 | -3.021361128 | 0.01032606 |
| LOC6043514 | protein FAM117B | -1.837635076 | 0.010351571 |
| LOC119766728 | uncharacterized LOC119766728 | 1.161329591 | 0.010400641 |
| LOC119767055 | histone-lysine N-methyltransferase SETD1A | -1.023590413 | 0.010409152 |
| LOC6039213 | glucose dehydrogenase [FAD, quinone] | -4.294064401 | 0.010516783 |
| LOC6038050 | zinc finger protein 79 | -1.254598794 | 0.010553601 |
| LOC6051732 | chitinase-3-like protein 2 | -1.16490771 | 0.010726604 |
| LOC6045167 | protein scarlet | 2.310209964 | 0.010757621 |
| LOC6038838 | G-protein coupled receptor 52 | -1.529430738 | 0.010790723 |
| LOC6040734 | mitogen-activated protein kinase kinase kinase 15 | -1.066870318 | 0.010800916 |
| LOC6039581 | repressor of filamentous growth 1 | -1.059724021 | 0.010866768 |
| LOC6040077 | tuberin | -1.028018364 | 0.010880281 |
| LOC6035024 | choline transporter-like protein 1 | -1.596377844 | 0.010929416 |
| LOC6037148 | pikachurin | -1.027050222 | 0.010929416 |
| LOC119769635 | uncharacterized LOC119769635 | 1.22880526 | 0.010946353 |
| LOC119769894 | uncharacterized LOC119769894 | 3.835317242 | 0.010946353 |
| LOC6036467 | chaoptin | -1.516612403 | 0.010969931 |
| LOC6033799 | zwei Ig domain protein zig-8 | -1.355990957 | 0.011039269 |
| LOC119770710 | uncharacterized LOC119770710 | 1.118280087 | 0.011125889 |
| LOC6033059 | leucine-rich PPR motif-containing protein, mitochondrial | -1.091764312 | 0.011276293 |
| LOC6045815 | leucine-rich repeats and immunoglobulin-like domains protein 2 | 1.428126048 | 0.011276293 |
| LOC6039207 | glucose dehydrogenase [FAD, quinone] | 2.11231108 | 0.011309839 |
| LOC6035932 | aquaporin-11 | 1.117030062 | 0.011337364 |
| LOC6049218 | xylulose kinase | -1.216443713 | 0.011418379 |
| LOC6036216 | CDGSH iron-sulfur domain-containing protein 3, mitochondrial | 1.093567598 | 0.011490463 |
| LOC6047830 | 5'-3' exoribonuclease 2 homolog | -1.187866562 | 0.011535883 |
| LOC119767415 | uncharacterized LOC119767415 | 1.958568737 | 0.011579022 |
| LOC6037548 | protein bicaudal C | -2.743063484 | 0.01213199 |
| LOC119769580 | uncharacterized LOC119769580 | 3.929219656 | 0.012273073 |
| LOC6039840 | glucosamine-6-phosphate isomerase | -1.16923116 | 0.012528683 |
| LOC119768893 | uncharacterized LOC119768893 | 2.164077956 | 0.01257833 |
| LOC6046181 | uncharacterized LOC6046181 | 1.126395138 | 0.012592284 |
| LOC6040770 | folylpolyglutamate synthase, mitochondrial | 1.032513067 | 0.012596186 |
| LOC119767780 | uncharacterized LOC119767780 | -1.955980523 | 0.012621587 |
| LOC6040168 | trafficking protein particle complex subunit 10 | -1.10987531 | 0.012638415 |
| LOC6035801 | uncharacterized LOC6035801 | -2.293691624 | 0.01267807 |
| LOC6034009 | tryptophan 2,3-dioxygenase | 1.13756066 | 0.012695654 |
| LOC6050629 | uncharacterized LOC6050629 | -1.775322867 | 0.012805512 |
| LOC6045627 | uncharacterized LOC6045627 | -1.011571452 | 0.012920202 |
| LOC119765570 | thioredoxin domain-containing protein 17-like | 1.04045951 | 0.013113012 |
| LOC6042285 | uncharacterized LOC6042285 | -1.541032347 | 0.013181144 |
| LOC119765507 | uncharacterized LOC119765507 | 3.541554995 | 0.013215473 |
| LOC119768082 | uncharacterized LOC119768082 | 3.533459802 | 0.013289348 |
| LOC6037683 | transcription termination factor 2 | -1.244634289 | 0.013291625 |
| LOC6040229 | ribosome biogenesis regulatory protein homolog | 1.214378796 | 0.013357085 |
| LOC6053933 | uncharacterized LOC6053933 | -1.14275926 | 0.013385169 |
| LOC6033723 | uncharacterized LOC6033723 | -1.044572851 | 0.013482674 |
| LOC119766360 | ninjurin-2-like | 2.470786748 | 0.013508197 |
| LOC6049616 | cytochrome P450 4C1 | 1.346729844 | 0.013508197 |
| LOC6032516 | perlucin | 2.124254979 | 0.013538576 |
| LOC6037687 | uncharacterized LOC6037687 | 2.042298025 | 0.013655598 |
| LOC6039552 | WD repeat-containing protein 92 | -1.616267396 | 0.013711269 |
| LOC6047617 | tyrosine-protein phosphatase 99A | -1.215360377 | 0.013847355 |
| LOC119770095 | cuticle protein 8-like | 1.83315916 | 0.013897971 |
| LOC6033349 | zinc finger protein 585A | 1.307666685 | 0.013905249 |
| LOC6054171 | nitric oxide synthase | -1.520949679 | 0.013940004 |
| LOC6051422 | probable small nuclear ribonucleoprotein E | 1.192301712 | 0.014220676 |
| LOC6043795 | A disintegrin and metalloproteinase with thrombospondin motifs adt-1 | -1.410334439 | 0.014338136 |
| LOC6047701 | uncharacterized LOC6047701 | 1.787993336 | 0.014355412 |
| LOC6049793 | flexible cuticle protein 12 | 1.732930059 | 0.014355412 |
| LOC6051255 | hydroxyacyl-coenzyme A dehydrogenase, mitochondrial | 1.111414985 | 0.014377458 |
| LOC119770580 | transmembrane protease serine 12-like | -1.055402931 | 0.014453635 |
| LOC6032824 | nurim homolog | 1.252517402 | 0.014453635 |
| LOC6054717 | protein archease-like | 1.03423209 | 0.014453635 |
| LOC119769904 | uncharacterized LOC119769904 | -3.975736784 | 0.014466278 |
| LOC6042069 | angiotensin-converting enzyme | -1.339822776 | 0.014466278 |
| LOC6049811 | nose resistant to fluoxetine protein 6 | 2.149751868 | 0.014782685 |
| LOC6046141 | endochitinase | -1.520446258 | 0.014795404 |
| LOC6047828 | trypsin-1 | 1.664696595 | 0.014857282 |
| LOC6047224 | clavesin-1 | -1.224338594 | 0.014883292 |
| LOC6031736 | tyrosine-protein kinase Btk29A | -1.113907404 | 0.014885934 |
| LOC6032086 | low molecular weight phosphotyrosine protein phosphatase | 2.953539669 | 0.014932851 |
| LOC119769841 | uncharacterized LOC119769841 | 1.266270695 | 0.014933695 |
| LOC6040137 | uncharacterized LOC6040137 | -1.054003654 | 0.014976465 |
| LOC6041046 | transmembrane protein 35A | 1.01551368 | 0.015040024 |
| LOC6035250 | uncharacterized LOC6035250 | 3.721149035 | 0.015193108 |
| LOC119767475 | putative helicase MOV-10 | -1.288921743 | 0.015223277 |
| LOC6051001 | sodium-independent sulfate anion transporter | -2.459081874 | 0.015254891 |
| LOC119768709 | uncharacterized LOC119768709 | 2.256682323 | 0.015746574 |
| LOC6035854 | protein slit | -1.435389316 | 0.015785156 |
| LOC6037964 | tyramine/octopamine receptor | -2.339373272 | 0.015879734 |
| LOC6039445 | vesicular glutamate transporter 2 | 1.139269801 | 0.015949468 |
| LOC6044377 | ubiquitin-like protein 5 | 1.211907144 | 0.016095134 |
| LOC6035857 | phospholipase A2 inhibitor | 1.230511746 | 0.016097101 |
| LOC6054065 | CDK5 and ABL1 enzyme substrate 2 | -1.078311195 | 0.016097101 |
| LOC6031624 | sodium-independent sulfate anion transporter | -1.149135149 | 0.016097169 |
| LOC6035574 | ATP-dependent RNA helicase SUV3 homolog, mitochondrial | 1.041207891 | 0.016100832 |
| LOC6031783 | ficolin-2 | 2.364291819 | 0.01620019 |
| LOC6051490 | protein O-mannosyl-transferase Tmtc3 | -1.412406215 | 0.016246168 |
| LOC6047230 | uncharacterized LOC6047230 | 1.813567361 | 0.016367253 |
| LOC6037424 | phosphopantothenate--cysteine ligase | -1.066593953 | 0.016487588 |
| LOC6046846 | perlucin | 2.631715095 | 0.016640385 |
| LOC119765539 | uncharacterized LOC119765539 | 4.165567473 | 0.016883487 |
| LOC6043540 | uncharacterized LOC6043540 | 1.981859179 | 0.017067933 |
| LOC6049016 | uncharacterized LOC6049016 | -1.435536841 | 0.017067933 |
| LOC6051477 | protein Wnt-2 | 1.065941055 | 0.017102593 |
| LOC6037439 | protein FRA10AC1 homolog | -1.159100856 | 0.017270376 |
| LOC119770000 | uncharacterized LOC119770000 | 1.292050084 | 0.017405368 |
| LOC6049911 | very long-chain specific acyl-CoA dehydrogenase, mitochondrial | 1.573112755 | 0.017453556 |
| LOC6036689 | RUS family member 1 | -1.160687721 | 0.01749615 |
| LOC6032544 | another transcription unit protein | -1.099963438 | 0.01770191 |
| LOC119769474 | uncharacterized LOC119769474 | 3.626551837 | 0.017855294 |
| LOC6049901 | uncharacterized LOC6049901 | 2.16151736 | 0.01790848 |
| LOC119769682 | uncharacterized LOC119769682 | 1.185188647 | 0.017955061 |
| LOC6043287 | uncharacterized LOC6043287 | 1.575481693 | 0.017955061 |
| LOC119767070 | uncharacterized LOC119767070 | -3.089135338 | 0.018075834 |
| LOC119769389 | uncharacterized LOC119769389 | -1.874007294 | 0.018113348 |
| LOC6032057 | serine protease Hayan | 1.127539656 | 0.018382994 |
| LOC6047471 | stearoyl-CoA desaturase 5 | -2.683017843 | 0.018382994 |
| LOC6040591 | bifunctional heparan sulfate N-deacetylase/N-sulfotransferase | -1.254457162 | 0.018700046 |
| LOC6043835 | uncharacterized LOC6043835 | 1.09699784 | 0.018700046 |
| LOC6039010 | protein male-specific lethal-3 | -1.294663933 | 0.018706692 |
| LOC6039606 | kelch-like ECH-associated protein 1B | -1.034958783 | 0.018874851 |
| LOC6048095 | uncharacterized LOC6048095 | -1.117854124 | 0.018939064 |
| LOC6054226 | gustatory receptor for sugar taste 43a | -1.628708663 | 0.019067281 |
| LOC6054374 | pupal cuticle protein Edg-91 | 3.931894521 | 0.019103065 |
| LOC6037431 | solute carrier family 26 member 10 | -2.503940322 | 0.019134714 |
| LOC6045251 | midnolin homolog | -1.238183085 | 0.01924968 |
| LOC119765573 | uncharacterized LOC119765573 | -2.770715593 | 0.019310248 |
| LOC6039048 | proton-coupled amino acid transporter-like protein CG1139 | 1.350054414 | 0.019311034 |
| LOC6042443 | uncharacterized LOC6042443 | 2.378760735 | 0.019481669 |
| LOC6033009 | dynein beta chain, ciliary | -1.489556307 | 0.019873186 |
| LOC6050931 | uncharacterized LOC6050931 | -1.706240592 | 0.019923754 |
| LOC6031923 | fibrinogen C domain-containing protein 1 | 3.733908832 | 0.02012092 |
| LOC6050486 | WD repeat-containing protein 89 | 1.663474519 | 0.020455698 |
| LOC6031850 | probable cytochrome P450 313a4 | 2.800257371 | 0.02050581 |
| LOC119768781 | uncharacterized LOC119768781 | -1.765702008 | 0.020830678 |
| LOC6047201 | prostasin | 1.289641717 | 0.020916615 |
| LOC6045192 | UDP-N-acetylglucosamine transferase subunit ALG13 homolog | 1.156600464 | 0.020925482 |
| LOC6041251 | uncharacterized LOC6041251 | 1.113204111 | 0.020928717 |
| LOC6036049 | zinc finger protein 11 | -2.196325891 | 0.021068482 |
| LOC6031876 | transmembrane protease serine 9 | 1.171470843 | 0.02109813 |
| LOC119770658 | coiled-coil domain-containing protein 58 | 1.210731162 | 0.021128242 |
| LOC6037926 | protein white | -1.135615597 | 0.021128242 |
| LOC6033346 | mediator of RNA polymerase II transcription subunit 15 | 1.061236348 | 0.021141843 |
| LOC6032866 | ribosomal L1 domain-containing protein CG13096 | 1.045299567 | 0.02148277 |
| LOC119766834 | sodium-dependent nutrient amino acid transporter 1-like | -1.342020115 | 0.021676909 |
| LOC6046588 | aminoacylase-1B | -2.543558843 | 0.021676909 |
| LOC6040512 | 39S ribosomal protein L33, mitochondrial | 1.064732858 | 0.021835995 |
| LOC6034193 | DNA-directed RNA polymerases I, II, and III subunit RPABC4 | 1.065084357 | 0.022051116 |
| LOC6047495 | UDP-glucosyltransferase 2 | 1.139149768 | 0.022093403 |
| LOC6045636 | U-scoloptoxin(16)-Ssd1a | 1.378790452 | 0.022107041 |
| LOC6044481 | N-alpha-acetyltransferase 80 | 1.508781076 | 0.022510471 |
| LOC6047453 | glycine receptor subunit alpha-3 | -1.28746161 | 0.022631147 |
| LOC6046213 | uncharacterized LOC6046213 | 1.811088414 | 0.022717749 |
| LOC6046471 | uncharacterized LOC6046471 | 1.12788551 | 0.022829496 |
| LOC6032291 | uncharacterized LOC6032291 | -1.697913194 | 0.023034411 |
| LOC6047446 | uncharacterized LOC6047446 | -1.16193781 | 0.023206799 |
| LOC119766762 | uncharacterized LOC119766762 | 1.079977125 | 0.023233765 |
| LOC6043796 | G2/mitotic-specific cyclin-B | -1.6645153 | 0.023241408 |
| LOC6034379 | ejaculatory bulb-specific protein 3 | 1.228301133 | 0.023290539 |
| LOC6035762 | putative odorant receptor 85d | 1.611885519 | 0.023471359 |
| LOC119769066 | uncharacterized LOC119769066 | 2.429714538 | 0.023518021 |
| LOC6032925 | estradiol 17-beta-dehydrogenase 8 | 1.281471813 | 0.023766936 |
| LOC6035140 | prostaglandin reductase 1 | 1.555360109 | 0.023856692 |
| LOC119767849 | larval/pupal cuticle protein H1C-like | 1.337259904 | 0.023960349 |
| LOC6040583 | uncharacterized LOC6040583 | -1.045594953 | 0.023960349 |
| LOC6050056 | angiotensin-converting enzyme | 1.075115015 | 0.02400248 |
| LOC6038630 | ras-GEF domain-containing family member 1B | -2.187520085 | 0.024079111 |
| LOC6033626 | adapter molecule Crk | -1.119875623 | 0.024286162 |
| LOC6040623 | spermine oxidase | 1.544954529 | 0.024295405 |
| LOC6035372 | titin | 1.398477596 | 0.024407742 |
| LOC6052111 | tetratricopeptide repeat protein 39C | -1.270224406 | 0.024813016 |
| LOC6042145 | uncharacterized LOC6042145 | -3.043143364 | 0.024902332 |
| LOC6037522 | zinc finger protein 2 homolog | -1.070616076 | 0.025117003 |
| LOC119768043 | adenylate cyclase-like | 2.874652985 | 0.025153463 |
| LOC6054293 | inositol monophosphatase 2 | -2.173830021 | 0.025305762 |
| LOC119769062 | uncharacterized LOC119769062 | -1.54446519 | 0.025336258 |
| LOC6047797 | cytochrome P450 9e2 | 1.231381058 | 0.025541244 |
| LOC119765319 | uncharacterized LOC119765319 | -2.202015886 | 0.025677356 |
| LOC119769840 | uncharacterized LOC119769840 | 1.192716024 | 0.025677356 |
| LOC6042197 | ribosome biogenesis protein NOP53 | 1.198316409 | 0.025677356 |
| LOC6036128 | drosulfakinins | 1.338382019 | 0.025712351 |
| LOC6035863 | putative fatty acyl-CoA reductase CG5065 | -2.281054611 | 0.026257044 |
| LOC6037920 | neural-cadherin | -1.431893093 | 0.026257044 |
| LOC6046728 | larval cuticle protein A2B | 2.435996955 | 0.026257044 |
| LOC6037241 | neuroglobin | -1.160173142 | 0.026384974 |
| LOC6051439 | homeobox protein SIX3 | 1.203961734 | 0.026557449 |
| LOC6032044 | chitooligosaccharidolytic beta-N-acetylglucosaminidase | -1.821254823 | 0.026566494 |
| LOC119767233 | fibroblast growth factor receptor homolog 2-like | 2.299254363 | 0.026579709 |
| LOC6052078 | cocaine esterase | 3.210211381 | 0.026645412 |
| LOC6041132 | small nuclear ribonucleoprotein-associated protein B' | 1.14153916 | 0.026781189 |
| LOC6041936 | protein arginine N-methyltransferase 1 | 1.394926214 | 0.026814417 |
| LOC6035201 | uncharacterized LOC6035201 | -1.11618211 | 0.026933006 |
| LOC6045116 | peptidyl-prolyl cis-trans isomerase FKBP2 | 1.012548244 | 0.027158634 |
| LOC6037782 | uncharacterized LOC6037782 | 1.047248545 | 0.027211686 |
| LOC6033812 | uncharacterized LOC6033812 | -1.083306222 | 0.027282191 |
| LOC6041796 | uncharacterized protein C1orf131 homolog | 1.000346706 | 0.027282191 |
| LOC6032063 | serine protease grass | -1.412174617 | 0.027409083 |
| LOC6038653 | tyrosine-protein phosphatase non-receptor type 23 | 1.267496059 | 0.027484573 |
| LOC6053794 | solute carrier family 2, facilitated glucose transporter member 3 | -1.64693476 | 0.027532391 |
| LOC6039393 | uncharacterized LOC6039393 | -2.181892536 | 0.027665707 |
| LOC6036502 | uncharacterized LOC6036502 | 1.311878888 | 0.028127546 |
| LOC6043538 | uncharacterized LOC6043538 | 1.072850845 | 0.02821258 |
| LOC119767639 | uncharacterized LOC119767639 | 1.041673095 | 0.02849315 |
| LOC6046297 | uncharacterized LOC6046297 | 2.708430626 | 0.028569967 |
| LOC6051165 | intracellular protein transport protein USO1 | -1.897817998 | 0.028768715 |
| LOC119766064 | uncharacterized LOC119766064 | -2.637620303 | 0.028780995 |
| LOC6049844 | uncharacterized LOC6049844 | 1.802929408 | 0.029125229 |
| LOC6048495 | microfibril-associated glycoprotein 4 | 1.620132694 | 0.029203378 |
| LOC119765336 | replication factor C subunit 5 | -1.275003666 | 0.029217532 |
| LOC6042820 | uncharacterized LOC6042820 | -1.364567052 | 0.029372932 |
| LOC6047103 | uncharacterized LOC6047103 | 1.329300733 | 0.02937548 |
| LOC6036412 | origin recognition complex subunit 4 | -1.273174162 | 0.029494699 |
| LOC6045170 | furin-like protease 1 | -1.007273092 | 0.030066373 |
| LOC119770850 | glutamate receptor ionotropic, kainate 2-like | 2.069070758 | 0.030095058 |
| LOC6037052 | estradiol 17-beta-dehydrogenase 11 | 1.988881764 | 0.030679902 |
| LOC6042094 | pupal cuticle protein 36 | -1.31360368 | 0.030777083 |
| LOC6047792 | uncharacterized LOC6047792 | -1.177598443 | 0.030798374 |
| LOC6034096 | transcription factor SPT20 homolog | -1.285526931 | 0.030817716 |
| LOC6039007 | gustatory and odorant receptor 24 | 1.154194044 | 0.030822743 |
| LOC6042437 | uncharacterized LOC6042437 | -2.131834913 | 0.030822743 |
| LOC6040790 | uncharacterized LOC6040790 | 2.153527293 | 0.031039693 |
| LOC6045063 | melanization protease 1 | 1.236972215 | 0.031191154 |
| LOC6044706 | cytochrome P450 4c21 | 1.554492279 | 0.031335276 |
| LOC119770760 | transcription initiation factor TFIID subunit 6-like | -1.050373446 | 0.031337813 |
| LOC6039942 | uncharacterized LOC6039942 | 2.431449916 | 0.03144434 |
| LOC6050923 | probable 2-oxoglutarate dehydrogenase E1 component DHKTD1 homolog, mitochondrial | 1.012569381 | 0.031446488 |
| LOC119767001 | uncharacterized LOC119767001 | -2.073652256 | 0.031496239 |
| LOC6031540 | uncharacterized LOC6031540 | 2.499476684 | 0.031608242 |
| LOC6032003 | augmin complex subunit dgt3 | -1.427806464 | 0.031716478 |
| LOC6053930 | uncharacterized LOC6053930 | 1.105056336 | 0.031801509 |
| LOC6033461 | uncharacterized LOC6033461 | 1.008297883 | 0.031803054 |
| LOC6032718 | valacyclovir hydrolase | 1.143377405 | 0.03219237 |
| LOC6050856 | PIN2/TERF1-interacting telomerase inhibitor 1 | 1.39811572 | 0.032255621 |
| LOC6041010 | uncharacterized LOC6041010 | -2.185098193 | 0.032388051 |
| LOC6037533 | uncharacterized LOC6037533 | 1.654258471 | 0.032485297 |
| LOC6038113 | uncharacterized LOC6038113 | 1.040150135 | 0.032730168 |
| LOC119769728 | uncharacterized LOC119769728 | -1.397923327 | 0.032735895 |
| LOC6048295 | zinc finger and SCAN domain-containing protein 12 | 1.445036011 | 0.032748883 |
| LOC119768943 | uncharacterized LOC119768943 | -1.861575282 | 0.032930931 |
| LOC119769831 | uncharacterized LOC119769831 | -2.415158229 | 0.032932715 |
| LOC6047809 | protein KRTCAP2 homolog | 1.37876727 | 0.03294059 |
| LOC6043954 | neuroligin-3 | -1.565281235 | 0.033109191 |
| LOC119768881 | uncharacterized LOC119768881 | -1.424515528 | 0.033220743 |
| LOC6045410 | zinc finger protein 660 | 1.330239666 | 0.033321931 |
| LOC119767167 | RNA-binding protein squid-like | -1.457158262 | 0.033349915 |
| LOC6047205 | carboxypeptidase N subunit 2 | -1.127817838 | 0.03339431 |
| LOC6040988 | clavesin-2 | 1.000352903 | 0.033583183 |
| LOC6045577 | WW domain-binding protein 4 | -1.101855399 | 0.033669054 |
| LOC6050679 | uncharacterized LOC6050679 | 2.132858534 | 0.033769975 |
| LOC6034673 | uncharacterized LOC6034673 | -1.974633965 | 0.033920965 |
| LOC119768795 | uncharacterized LOC119768795 | -1.100483202 | 0.034041661 |
| LOC6040441 | 39S ribosomal protein L52, mitochondrial | 1.144175895 | 0.034142212 |
| LOC6041116 | nose resistant to fluoxetine protein 6 | -2.018704799 | 0.034427717 |
| LOC6034175 | dynein heavy chain 12, axonemal | -2.2585041 | 0.035014071 |
| LOC6041539 | uncharacterized LOC6041539 | 1.125781706 | 0.035098456 |
| LOC6039906 | guanosine-3',5'-bis(diphosphate) 3'-pyrophosphohydrolase MESH1 | 1.085828373 | 0.035449029 |
| LOC6050650 | E3 ubiquitin-protein ligase TRIM37 | -2.238772295 | 0.035449029 |
| LOC119766781 | uncharacterized LOC119766781 | 1.904132624 | 0.035744062 |
| LOC119766940 | uncharacterized LOC119766940 | 1.394466817 | 0.03582528 |
| LOC6033627 | protein nubbin | -1.120674561 | 0.035881068 |
| LOC6037391 | uncharacterized LOC6037391 | -1.240415196 | 0.036000988 |
| LOC6046736 | cuticle protein 8 | 2.047166108 | 0.036069087 |
| LOC6033844 | F-box/LRR-repeat protein 7 | -1.258698936 | 0.036338707 |
| LOC6047660 | uncharacterized LOC6047660 | -1.152772446 | 0.036338707 |
| LOC6040693 | odorant receptor 94a | 1.471679414 | 0.036559744 |
| LOC6044781 | uncharacterized LOC6044781 | -1.151661908 | 0.036580228 |
| LOC6040926 | uncharacterized LOC6040926 | 2.498258875 | 0.036961528 |
| LOC119770878 | 5.8S ribosomal RNA | 2.12358979 | 0.037231479 |
| LOC119770176 | uncharacterized LOC119770176 | 1.792821447 | 0.037306462 |
| LOC119766416 | uncharacterized LOC119766416 | -1.113799338 | 0.037385015 |
| LOC6043742 | hemicentin-1 | -1.061699378 | 0.03767691 |
| LOC6034956 | coiled-coil domain-containing protein 151 | -1.915306404 | 0.037723789 |
| LOC6047331 | chitooligosaccharidolytic beta-N-acetylglucosaminidase | 2.095693342 | 0.03777189 |
| LOC6043449 | zinc finger protein 493 | -1.444589382 | 0.037800178 |
| LOC6033843 | uncharacterized LOC6033843 | 1.053167422 | 0.038098904 |
| LOC6036912 | zinc finger protein 708 | 1.298877046 | 0.038121161 |
| LOC6043139 | carboxypeptidase B | 1.47025254 | 0.038185777 |
| LOC6038879 | protein C1orf194 | -1.046302192 | 0.038280016 |
| LOC6040486 | arrestin domain-containing protein 3 | 1.048386898 | 0.038284364 |
| LOC119770469 | uncharacterized LOC119770469 | 1.344714731 | 0.038430936 |
| LOC6037490 | probable cytochrome P450 6a14 | 1.151791424 | 0.039000533 |
| LOC6031402 | neuroguidin | 1.097781722 | 0.039088664 |
| LOC6052586 | dual specificity protein phosphatase MPK-4 | -1.418987271 | 0.040218751 |
| LOC6041808 | cytochrome P450 4c3 | -1.222769597 | 0.0404842 |
| LOC6047675 | antigen 5 like allergen Cul n 1 | 1.351674833 | 0.040954177 |
| LOC6031152 | uncharacterized LOC6031152 | 1.134743973 | 0.041229895 |
| LOC6039413 | cysteinyl leukotriene receptor 1 | -1.203665235 | 0.041272686 |
| LOC6031772 | uncharacterized protein C19orf47 | -1.010170324 | 0.041431389 |
| LOC6052070 | CLIP domain-containing serine protease 14D | 1.001324231 | 0.041599305 |
| LOC119768701 | uncharacterized LOC119768701 | 2.053026005 | 0.041713133 |
| LOC6050827 | uncharacterized LOC6050827 | 1.157864179 | 0.041719434 |
| LOC6045741 | uncharacterized LOC6045741 | -1.070595416 | 0.041866032 |
| LOC6042398 | proline-, glutamic acid- and leucine-rich protein 1 | 1.092447666 | 0.042026525 |
| LOC119766576 | uncharacterized LOC119766576 | -1.306032956 | 0.04233738 |
| LOC6049867 | uncharacterized LOC6049867 | -1.572989259 | 0.042580697 |
| LOC119766835 | sodium-dependent nutrient amino acid transporter 1-like | 1.238358913 | 0.042694128 |
| LOC6051162 | lipase member H | 1.249479625 | 0.042775872 |
| LOC6048005 | probable cytochrome P450 4d14 | -1.095409327 | 0.042890017 |
| LOC6053204 | elongation of very long chain fatty acids protein | -1.017484676 | 0.042901428 |
| LOC6050141 | chitin synthase chs-2 | -1.39296124 | 0.043282982 |
| LOC6039670 | chorion peroxidase | -1.005232982 | 0.043502585 |
| LOC119767033 | uncharacterized LOC119767033 | 1.240814978 | 0.043706161 |
| LOC6034433 | mpv17-like protein | 1.110898237 | 0.043884563 |
| LOC6052230 | zinc finger and BTB domain-containing protein 14 | -1.131623566 | 0.044233101 |
| LOC6042626 | zinc finger protein OZF | -1.120070941 | 0.044818644 |
| LOC119765484 | uncharacterized LOC119765484 | -1.085871849 | 0.044847302 |
| LOC6032000 | atrial natriuretic peptide receptor 1 | -1.316686439 | 0.044943839 |
| LOC6041339 | protein RRNAD1 | -1.560767933 | 0.044943839 |
| LOC6036782 | myb-like protein Q | 1.450074008 | 0.045212738 |
| LOC6035754 | uncharacterized LOC6035754 | 1.96717006 | 0.045372237 |
| LOC6035747 | cell adhesion molecule 4 | 1.75956691 | 0.045386153 |
| LOC119769492 | zinc finger protein 771-like | -1.783725122 | 0.045420749 |
| LOC6033904 | ankyrin repeat and KH domain-containing protein mask | -1.236868143 | 0.045895181 |
| LOC6044739 | uncharacterized LOC6044739 | -1.195439326 | 0.045901472 |
| LOC6046508 | maltase 2 | -1.622874832 | 0.046709065 |
| LOC6053554 | uncharacterized LOC6053554 | 1.121782612 | 0.046974733 |
| LOC6046049 | thyrostimulin alpha-2 subunit | 1.049465072 | 0.047204324 |
| LOC6037563 | glutathione peroxidase | 1.83667028 | 0.047249245 |
| LOC6041051 | ionotropic receptor 75a | 1.50698114 | 0.047276402 |
| LOC6040818 | uncharacterized LOC6040818 | 1.545727974 | 0.04740649 |
| LOC6054454 | TBC1 domain family member 19 | 1.067666707 | 0.047441018 |
| LOC6048289 | sodium- and chloride-dependent glycine transporter 2 | 1.803776178 | 0.047567073 |
| LOC6033159 | uncharacterized LOC6033159 | 1.638784747 | 0.048378955 |
| LOC6037900 | LIM domain transcription factor LMO4 | -1.147404731 | 0.048691163 |
| LOC6047740 | histidine-rich glycoprotein | 1.860137919 | 0.049086619 |
| LOC6052404 | carbonic anhydrase 2 | 1.353190295 | 0.049098852 |
| LOC119767380 | uncharacterized LOC119767380 | -1.676486218 | 0.04935167 |
| LOC6040045 | phospholipase A1 member A | 1.713205581 | 0.049420779 |
| LOC6035836 | DNA repair endonuclease XPF | -1.125428913 | 0.04963983 |
| LOC6048244 | caspase-8 | 1.063416122 | 0.04995117 |

Table S5. Inferred *Culex* and *Wyeomyia* orthologs based on *Culex quinquefasciatus* locus tags (CPIJ_ID). M-value refers to *Wyeomyia* differential expression in biters relative to non-biters in microarray experiments as described in Bradshaw et al. (2018). Culex Log2FC refers to Log2 fold change based in biting Pipiens relative to non-biting molestus based on RNAseq experiments.

| **Ws_contig** | **Ws_**  **M-value** | **Ws_pvalue** | **culex_LOCID** | **Culex_Log2FC** | **culex_adjp** | **culex_ID** |
| --- | --- | --- | --- | --- | --- | --- |
| CONTIG02166 | -1.2819012 | 0.00059 | LOC6031152 | 1.134744 | 0.0412299 | CPIJ000230 |
| CONTIG12831 | 1.26446781 | 2.93E-07 | LOC6031256 | 1.4382287 | 0.0041723 | CPIJ000416 |
| CONTIG07304 | -0.8880373 | 0.00023 | LOC6031538 | 1.8263271 | 2.83E-07 | CPIJ000560 |
| F5BTJ3O01BUJKZ | -1.2834183 | 5.52E-05 | LOC6031460 | 1.4444491 | 4.53E-06 | CPIJ000699 |
| CONTIG11435 | 0.82886933 | 0.00018 | LOC6031475 | -1.0036921 | 0.0055456 | CPIJ000710 |
| CONTIG11156 | -1.0834435 | 0.00142 | LOC6031511 | 1.766982 | 0.0023711 | CPIJ000736 |
| CONTIG17612 | -0.7205101 | 0.0004 | LOC6031876 | 1.1714708 | 0.0210981 | CPIJ000768 |
| F5BTJ3O02HYSPN | -1.3800163 | 3.68E-06 | LOC6031732 | 1.5614705 | 7.17E-05 | CPIJ000830 |
| CONTIG09924 | -0.5555263 | 6.23E-05 | LOC6031767 | -1.1129195 | 6.45E-05 | CPIJ000855 |
| CONTIG04485 | -1.193781 | 0.00095 | LOC6032249 | 1.3506732 | 2.69E-06 | CPIJ001218 |
| CONTIG03864 | -0.9555822 | 0.0015 | LOC6032307 | 1.0772641 | 3.83E-05 | CPIJ001270 |
| CONTIG05860 | -0.8279251 | 0.0012 | LOC6032330 | -1.7052765 | 0.0014554 | CPIJ001291 |
| CONTIG03819 | -0.7552618 | 0.00059 | LOC6032731 | 1.9226683 | 1.28E-05 | CPIJ001380 |
| CONTIG00368 | -1.110508 | 0.00124 | LOC6032382 | 1.9690487 | 0.0023162 | CPIJ001435 |
| CONTIG05057 | -0.6437788 | 0.00057 | LOC6032392 | 1.0104684 | 0.0009041 | CPIJ001438 |
| F5BTJ3O01BLGSQ | 0.45702914 | 0.00156 | LOC6033345 | 2.4395626 | 4.12E-07 | CPIJ001507 |
| F5BTJ3O01BF2UC | -1.0639555 | 0.00109 | LOC6033118 | -2.8562618 | 1.45E-12 | CPIJ001748 |
| CONTIG09477 | 1.01571808 | 0.00019 | LOC6032966 | 4.5789823 | 0.0100102 | CPIJ001808 |
| CONTIG00862 | 0.39850404 | 0.00098 | LOC6032982 | 4.6807467 | 4.77E-07 | CPIJ001820 |
| CONTIG13315 | 0.97574915 | 0.00028 | LOC6032985 | 1.6528021 | 0.0030678 | CPIJ001822 |
| CONTIG11387 | 0.42483901 | 0.00097 | LOC6033284 | 1.2172318 | 7.60E-05 | CPIJ001977 |
| F5BTJ3O01CKVBI | -1.0545023 | 0.00086 | LOC6032792 | 1.3917754 | 0.0001302 | CPIJ002063 |
| CONTIG20938 | -1.3246487 | 1.60E-05 | LOC6032836 | 1.4266282 | 0.0037999 | CPIJ002105 |
| CONTIG04231 | -0.5334739 | 0.00041 | LOC6032866 | 1.0452996 | 0.0214828 | CPIJ002123 |
| CONTIG13126 | 1.18411487 | 0.00096 | LOC6033704 | 1.1909283 | 1.11E-05 | CPIJ002383 |
| CONTIG04553 | -1.0891454 | 0.00155 | LOC6033631 | 1.5151582 | 0.0019003 | CPIJ002449 |
| F5BTJ3O02H4HN2 | -1.0788262 | 1.37E-05 | LOC6033844 | -1.2586989 | 0.0363387 | CPIJ002568 |
| F5BTJ3O02IZ3CS | -1.151383 | 3.38E-06 | LOC6033904 | -1.2368681 | 0.0458952 | CPIJ002710 |
| F5BTJ3O02IUEOM | -1.72366 | 1.62E-07 | LOC6033929 | 3.3773483 | 0.0007944 | CPIJ002735 |
| F5BTJ3O02GSH5X | -0.5453112 | 0.001 | LOC6033964 | -1.815918 | 0.0022829 | CPIJ002769 |
| CONTIG11284 | -0.643531 | 0.00101 | LOC6033979 | -1.4862624 | 1.60E-09 | CPIJ002784 |
| CONTIG15225 | -0.9494891 | 0.00063 | LOC6034000 | 1.2334999 | 0.0002528 | CPIJ002856 |
| CONTIG02993 | 1.67604154 | 1.96E-05 | LOC6034631 | 2.7039964 | 4.97E-06 | CPIJ003030 |
| CONTIG01126 | -0.6749236 | 0.00113 | LOC6034116 | 1.4960802 | 1.64E-06 | CPIJ003098 |
| CONTIG06957 | -0.5259691 | 0.00087 | LOC6034765 | -1.3204917 | 0.0007244 | CPIJ003312 |
| CONTIG23005 | 4.24558119 | 1.45E-06 | LOC6034839 | 2.8245246 | 3.97E-05 | CPIJ003470 |
| CONTIG19133 | 4.24981375 | 1.48E-06 | LOC6034842 | 3.5509176 | 1.22E-09 | CPIJ003473 |
| CONTIG08236 | 4.43489048 | 2.91E-07 | LOC6034843 | 3.802754 | 0.00053 | CPIJ003474 |
| CONTIG05099 | 0.71971726 | 6.10E-05 | LOC6034907 | 1.5523794 | 0.0001393 | CPIJ003534 |
| CONTIG12772 | 0.88967143 | 5.77E-05 | LOC6035075 | 1.5242384 | 3.99E-07 | CPIJ003738 |
| F5BTJ3O01DEICM | -0.5426086 | 0.00073 | LOC6035646 | 5.1216694 | 2.83E-09 | CPIJ004117 |
| F5BTJ3O01B9GT1 | -1.1907759 | 4.13E-05 | LOC6035808 | 3.5927509 | 1.44E-06 | CPIJ004288 |
| CONTIG01929 | 2.32909355 | 6.27E-05 | LOC6035976 | -5.9346245 | 2.01E-31 | CPIJ004497 |
| CONTIG07587 | -0.9605123 | 0.0011 | LOC6036269 | 1.014242 | 0.0027439 | CPIJ004571 |
| F5BTJ3O02ICMT0 | -1.5452106 | 4.01E-05 | LOC6036301 | 2.9573498 | 0.0036812 | CPIJ004603 |
| CONTIG14472 | 1.17550189 | 7.83E-05 | LOC6036330 | 1.474839 | 2.86E-05 | CPIJ004782 |
| F5BTJ3O02JZ5HS | -1.1499904 | 0.00054 | LOC6036459 | 1.0015284 | 0.0013556 | CPIJ004860 |
| CONTIG02476 | -0.6897766 | 0.00165 | LOC6036411 | 1.3062499 | 0.002944 | CPIJ004943 |
| F5BTJ3O01EV156 | -1.4988674 | 8.77E-05 | LOC6036776 | 1.2174419 | 0.005638 | CPIJ005330 |
| F5BTJ3O01AS4SP | -1.2094992 | 0.00059 | LOC6037321 | 1.4479041 | 1.92E-06 | CPIJ005762 |
| CONTIG02438 | 0.90332476 | 0.00102 | LOC6037346 | 1.1940499 | 9.05E-06 | CPIJ005785 |
| CONTIG11327 | -1.2248898 | 0.00087 | LOC6037992 | 3.01238 | 1.24E-08 | CPIJ005818 |
| CONTIG06422 | -1.3997502 | 6.67E-05 | LOC6037828 | 1.0166105 | 7.50E-05 | CPIJ005878 |
| CONTIG09504 | -1.294054 | 0.00043 | LOC6037470 | 1.0855016 | 0.006705 | CPIJ005932 |
| F5BTJ3O01DPAUU | -0.8021519 | 0.00059 | LOC6037391 | -1.2404152 | 0.036001 | CPIJ005996 |
| CONTIG09777 | -1.2726981 | 0.00016 | LOC6038312 | -1.0607111 | 0.005004 | CPIJ006382 |
| CONTIG01510 | 3.78232379 | 5.99E-08 | LOC6038592 | -1.6869408 | 0.0009417 | CPIJ006594 |
| CONTIG18717 | -1.2582904 | 0.00041 | LOC6038275 | 1.425201 | 9.48E-08 | CPIJ006623 |
| CONTIG05232 | -1.2624804 | 0.00043 | LOC6038431 | 1.1409427 | 6.85E-07 | CPIJ006671 |
| F5BTJ3O02JHNJQ | -0.676655 | 0.00024 | LOC6039503 | -1.3532657 | 0.0004695 | CPIJ006731 |
| CONTIG13991 | -0.8704608 | 0.00084 | LOC6038339 | 1.0258263 | 2.69E-05 | CPIJ006763 |
| CONTIG00927 | -0.6232456 | 0.00107 | LOC6039379 | 1.0036042 | 2.71E-05 | CPIJ006946 |
| CONTIG13190 | 1.16915262 | 0.00049 | LOC6039397 | 1.6306387 | 8.13E-05 | CPIJ006963 |
| CONTIG18037 | -0.9289832 | 0.00094 | LOC6038882 | 1.3823564 | 0.0019813 | CPIJ007052 |
| CONTIG06017 | -0.5306012 | 0.00061 | LOC6038948 | 1.0822884 | 0.0010332 | CPIJ007470 |
| CONTIG09688 | -1.270892 | 0.00018 | LOC6040134 | 1.4348951 | 8.92E-10 | CPIJ007698 |
| CONTIG07436 | -0.5538709 | 0.00044 | LOC6040012 | 1.7305551 | 3.54E-08 | CPIJ007823 |
| F5BTJ3O01C8VO5 | -1.0265028 | 0.00097 | LOC6040567 | 1.2029819 | 1.68E-05 | CPIJ007993 |
| F5BTJ3O01BE680 | 0.90128946 | 0.0002 | LOC6039942 | 2.4314499 | 0.0314443 | CPIJ008187 |
| F5BTJ3O01DDWGJ | -0.7510726 | 0.001 | LOC6039896 | 1.200078 | 0.0008257 | CPIJ008278 |
| CONTIG13136 | 0.85094109 | 4.72E-05 | LOC6039905 | 1.252875 | 0.0005367 | CPIJ008287 |
| F5BTJ3O01CLB51 | -0.8290074 | 0.00016 | LOC6039906 | 1.0858284 | 0.035449 | CPIJ008288 |
| F5BTJ3O01CUBMM | -1.0784054 | 0.00109 | LOC6040499 | 1.218224 | 0.0018087 | CPIJ008389 |
| CONTIG08513 | -0.6670402 | 9.51E-05 | LOC6040734 | -1.0668703 | 0.0108009 | CPIJ008487 |
| F5BTJ3O01D8KDB | -1.1768764 | 0.00035 | LOC6041010 | -2.1850982 | 0.0323881 | CPIJ008536 |
| CONTIG12329 | 1.36734281 | 0.00067 | LOC6040955 | 1.1821907 | 1.23E-08 | CPIJ008542 |
| CONTIG00722 | 0.86866738 | 0.00099 | LOC6041015 | 1.1857955 | 0.0023439 | CPIJ008587 |
| CONTIG07299 | -1.0805585 | 0.00016 | LOC6041796 | 1.0003467 | 0.0272822 | CPIJ008927 |
| CONTIG13010 | -1.611966 | 6.98E-05 | LOC6041266 | 1.9922904 | 0.0026531 | CPIJ008974 |
| CONTIG00885 | 1.12939319 | 0.00138 | LOC6041441 | 6.6268876 | 1.44E-39 | CPIJ009033 |
| CONTIG04910 | 5.09361883 | 1.33E-07 | LOC6042062 | 2.7104125 | 1.35E-09 | CPIJ009100 |
| CONTIG17903 | 4.07743501 | 1.06E-07 | LOC6042063 | 1.6068974 | 1.03E-07 | CPIJ009101 |
| CONTIG12142 | 2.43434308 | 6.74E-06 | LOC6042072 | 3.9261379 | 6.16E-08 | CPIJ009109 |
| CONTIG11370 | 3.54578679 | 6.91E-08 | LOC6042074 | 2.1989872 | 6.69E-07 | CPIJ009111 |
| CONTIG08536 | -1.3651493 | 0.00024 | LOC6041884 | 1.2337874 | 6.86E-06 | CPIJ009411 |
| CONTIG08510 | 0.48280957 | 0.0003 | LOC6041917 | 1.1942665 | 7.32E-05 | CPIJ009438 |
| F5BTJ3O02IARMS | -1.0374187 | 0.00161 | LOC6042208 | 1.3989711 | 9.46E-08 | CPIJ009651 |
| F5BTJ3O02HA9Y3 | -1.0123393 | 8.32E-06 | LOC6042434 | 12.523626 | 8.44E-16 | CPIJ009829 |
| CONTIG06732 | -1.0841372 | 0.00056 | LOC6042322 | 1.9414421 | 0.0045571 | CPIJ009900 |
| CONTIG04236 | -0.7880285 | 0.00122 | LOC6043021 | 1.1615221 | 8.86E-07 | CPIJ010114 |
| CONTIG02560 | -0.6750611 | 0.00156 | LOC6054402 | 1.3220386 | 8.08E-08 | CPIJ010281 |
| F5BTJ3O02FXME2 | -1.7851962 | 3.26E-09 | LOC6042953 | -1.8425762 | 7.44E-05 | CPIJ010395 |
| CONTIG10652 | -0.9706425 | 0.00111 | LOC6043301 | 1.4871574 | 0.0050791 | CPIJ010404 |
| F5BTJ3O02FUX9T | 0.49563554 | 0.00051 | LOC6042975 | 1.0121783 | 1.96E-06 | CPIJ010645 |
| CONTIG05477 | -1.1740834 | 0.00085 | LOC6043123 | 1.0433684 | 0.0005328 | CPIJ010692 |
| CONTIG09214 | -1.1374898 | 0.00101 | LOC6043699 | 1.0576444 | 8.92E-06 | CPIJ010827 |
| CONTIG18265 | -1.0578754 | 0.00043 | LOC6043200 | 1.0347852 | 0.0011771 | CPIJ010918 |
| CONTIG09139 | -0.9228146 | 0.00023 | LOC6044008 | 1.1551501 | 3.47E-05 | CPIJ011350 |
| F5BTJ3O01EWOIY | -1.1604854 | 0.00138 | LOC6044377 | 1.2119071 | 0.0160951 | CPIJ011517 |
| CONTIG01167 | 0.96426045 | 0.00145 | LOC6045132 | 1.4285812 | 7.84E-09 | CPIJ011791 |
| CONTIG02111 | 4.03885652 | 2.17E-07 | LOC6045354 | 3.6967346 | 3.03E-13 | CPIJ012466 |
| F5BTJ3O01C3VBA | -0.6552888 | 6.03E-05 | LOC6045194 | 1.5306638 | 0.0040175 | CPIJ012477 |
| CONTIG12595 | -0.6047733 | 0.0004 | LOC6045063 | 1.2369722 | 0.0311912 | CPIJ012502 |
| CONTIG00354 | 1.33942164 | 2.49E-05 | LOC6045070 | 3.0462536 | 0.0018529 | CPIJ012507 |
| CONTIG24010 | 1.58147882 | 0.00037 | LOC6045432 | 1.0603695 | 0.0035972 | CPIJ012570 |
| CONTIG10792 | 1.25763698 | 4.07E-05 | LOC6046141 | -1.5204463 | 0.0147954 | CPIJ013082 |
| CONTIG07077 | -0.9958186 | 0.00032 | LOC6046094 | 1.0942108 | 0.0055537 | CPIJ013222 |
| F5BTJ3O01D6GZQ | 1.47564596 | 0.00015 | LOC6047137 | 1.4645522 | 2.63E-08 | CPIJ013361 |
| F5BTJ3O02HEOIC | -0.8139133 | 0.00018 | LOC6046560 | -1.3164165 | 0.000595 | CPIJ013499 |
| F5BTJ3O02HND1O | -1.0372308 | 0.00027 | LOC6046560 | -1.3164165 | 0.000595 | CPIJ013499 |
| F5BTJ3O01E2MLQ | -0.682523 | 0.00039 | LOC6046495 | -8.3642601 | 3.91E-05 | CPIJ013724 |
| CONTIG07106 | -1.2224425 | 4.89E-06 | LOC6046726 | 8.0313438 | 0.0002495 | CPIJ013784 |
| CONTIG19722 | -0.9341026 | 0.00129 | LOC6046728 | 2.435997 | 0.026257 | CPIJ013786 |
| CONTIG02953 | -0.7571435 | 0.00061 | LOC6048459 | 1.4393608 | 6.83E-08 | CPIJ013899 |
| CONTIG18745 | -0.82088 | 4.28E-05 | LOC6047252 | -1.2945321 | 0.0090565 | CPIJ013920 |
| CONTIG04644 | -1.1827735 | 0.00073 | LOC6047732 | 1.0276468 | 0.0007379 | CPIJ013966 |
| CONTIG10321 | -1.0410011 | 0.00092 | LOC6047052 | 1.3240064 | 1.69E-05 | CPIJ014062 |
| F5BTJ3O02HN4B3 | -2.1109131 | 3.35E-07 | LOC6046856 | -1.0008828 | 0.0022496 | CPIJ014111 |
| CONTIG19946 | -0.9934771 | 0.00136 | LOC6047090 | 1.0320057 | 0.0002894 | CPIJ014165 |
| CONTIG04652 | -0.6872687 | 9.72E-05 | LOC6047234 | 1.4299447 | 0.0012248 | CPIJ014239 |
| CONTIG06460 | 0.86065732 | 0.00052 | LOC6047714 | 1.177722 | 1.41E-06 | CPIJ014296 |
| CONTIG14090 | -1.7943488 | 7.97E-05 | LOC6047791 | 3.1927743 | 1.49E-05 | CPIJ014327 |
| CONTIG20541 | -0.8158467 | 0.00018 | LOC6048136 | 2.1460516 | 0.0087753 | CPIJ014778 |
| CONTIG06896 | 1.75877488 | 1.74E-06 | LOC6048046 | -1.4040686 | 8.31E-05 | CPIJ014933 |
| CONTIG21034 | -1.327212 | 0.00035 | LOC6048573 | 1.0474662 | 0.0002944 | CPIJ015042 |
| CONTIG16041 | -0.800332 | 0.00023 | LOC6048278 | 1.4121272 | 0.0001556 | CPIJ015056 |
| CONTIG06809 | 1.26159971 | 0.00055 | LOC6048349 | 1.3247653 | 0.0001137 | CPIJ015335 |
| CONTIG18888 | 0.63541983 | 0.00047 | LOC6048537 | -1.2359672 | 4.24E-05 | CPIJ015463 |
| CONTIG12179 | 1.05711726 | 8.08E-05 | LOC6049236 | 3.0454973 | 6.16E-08 | CPIJ015743 |
| F5BTJ3O01DGITJ | 1.34587816 | 0.00016 | LOC6049222 | -7.9411851 | 9.94E-12 | CPIJ015761 |
| CONTIG00852 | -0.8779934 | 0.00116 | LOC6049723 | 2.491598 | 5.61E-17 | CPIJ015882 |
| CONTIG02559 | -0.6025066 | 0.00145 | LOC6049626 | 1.3704447 | 0.0012459 | CPIJ015964 |
| CONTIG12386 | 1.94329334 | 1.54E-05 | LOC6049711 | 1.8273748 | 0.0001251 | CPIJ016129 |
| F5BTJ3O01AW2F7 | 2.17582583 | 4.95E-06 | LOC6050002 | 3.2768443 | 1.12E-07 | CPIJ016210 |
| CONTIG01807 | 1.1071476 | 0.00115 | LOC6050141 | -1.3929612 | 0.043283 | CPIJ016255 |
| CONTIG19861 | 2.83762093 | 3.56E-06 | LOC6049791 | 1.6170202 | 1.12E-06 | CPIJ016316 |
| CONTIG13674 | -0.5237254 | 0.00044 | LOC6049700 | 1.6071287 | 0.001316 | CPIJ016374 |
| CONTIG23942 | 1.07547223 | 0.00051 | LOC6050225 | 1.320173 | 3.67E-05 | CPIJ016462 |
| CONTIG04471 | 1.97263657 | 7.30E-05 | LOC6050265 | 5.018288 | 0.0078391 | CPIJ016509 |
| F5BTJ3O01A15O5 | 0.68840298 | 0.00024 | LOC6036302 | -1.0018099 | 0.002553 | CPIJ016918 |
| CONTIG00141 | -0.8527545 | 0.00131 | LOC6036297 | 1.7086365 | 2.24E-05 | CPIJ016922 |
| F5BTJ3O01CA5TV | -0.7698463 | 0.00066 | LOC6050480 | 5.9108354 | 2.59E-58 | CPIJ016927 |
| CONTIG05744 | 0.98911137 | 0.0016 | LOC6053994 | 9.1892986 | 2.70E-80 | CPIJ017024 |
| CONTIG02271 | -0.9248561 | 0.0014 | LOC6050856 | 1.3981157 | 0.0322556 | CPIJ017220 |
| CONTIG05465 | -1.2679605 | 4.89E-06 | LOC6050932 | 1.2533819 | 0.0005564 | CPIJ017286 |
| CONTIG03998 | 0.6827957 | 0.00063 | LOC6051047 | -1.0178108 | 0.0031061 | CPIJ017440 |
| F5BTJ3O01EFOBF | -1.6031478 | 4.48E-05 | LOC6051379 | 1.8851744 | 0.0009292 | CPIJ017495 |
| CONTIG19956 | 4.52185122 | 9.89E-08 | LOC6051867 | 3.299131 | 2.97E-14 | CPIJ017874 |
| F5BTJ3O01BF5EM | -1.1858592 | 1.90E-06 | LOC6051803 | 3.3014934 | 0.0020532 | CPIJ017967 |
| CONTIG08527 | -1.225107 | 0.00113 | LOC6051904 | 1.1219577 | 0.0012143 | CPIJ018054 |
| CONTIG00351 | 0.81440349 | 0.0008 | LOC6052229 | -1.5295541 | 4.31E-11 | CPIJ018102 |
| CONTIG03428 | 0.69925837 | 0.00126 | LOC6040384 | 1.5473596 | 0.0003883 | CPIJ018369 |
| F5BTJ3O02FWTR9 | -0.8308428 | 0.00045 | LOC6052404 | 1.3531903 | 0.0490989 | CPIJ018437 |
| CONTIG14592 | -1.0404273 | 0.00154 | LOC6052583 | 1.3700918 | 1.58E-05 | CPIJ018444 |
| CONTIG05281 | -0.5111315 | 0.0002 | LOC6052987 | 6.9833582 | 1.16E-35 | CPIJ018858 |
| F5BTJ3O01BJT8Z | -1.5445879 | 1.08E-06 | LOC6053712 | 4.4938563 | 2.12E-12 | CPIJ019498 |
| CONTIG01254 | 0.73965396 | 6.82E-06 | LOC6053930 | 1.1050563 | 0.0318015 | CPIJ019681 |

Table S6: Differences in mRNA abundance between biting *Cx. pipiens pipiens* relative to non-biting *Cx. pipiens molestus* are generally consistent in independent samples when measured with either RNAseq or qRT-PCR**.**

| Gene ID | Gene Name | RNA seq results | | qRT-PCR Results | |
| --- | --- | --- | --- | --- | --- |
|  |  | Log2 Fold change | Adjusted p-value | Log2 fold change | p-value |
| LOC6052987 | *angiopoietin-related protein 6* | 6.98 | 1.16E-35 | 6.78 | 0.00110 |
| LOC6037495 | *probable cytochrome P450 6a14* | 3.72 | 1.93E-20 | 4.60 | 0.000944 |
| LOC6046433 | *ficolin-3* | 4.87 | 7.67E-28 | 3.76 | 1.7E-05 |
| LOC6041441 | *hexamerin-1.1* | 6.63 | 1.44E-39 | 1.76 | 0.102 |
| LOC6033003 | *larval cuticle protein A3A* | 9.40 | 2.15E-20 | -0.70 | 0.197 |
| LOC6031357 | *cuticle protein 38* | 9.52 | 5.09E-22 | 0.43 | 0.298 |
| LOC6043252 | *vitellogenin-A1* | -9.65 | 1.42E-11 | -9.55 | 0.0242 |
| LOC6052229 | *fumarylacetoacetase* | -1.53 | 4.31E-11 | -3.19 | 0.0319 |
| LOC6030831 | *esterase B1* | -10.80 | 2.91E-11 | -10.93 | 0.0308 |
| LOC6049222 | *cathepsin B* | -7.94 | 9.94E-12 | -12.15 | 0.0371 |
| LOC6037771 | *L-galactose dehydrogenase* | -7.48 | 8.39E-12 | -11.73 | 0.0178 |

Figure S1. Principle components plot of gene expression profiles in *Cx. p. molestus* (non-biting) *Cx. p. pipiens* (biting) samples. M (red) dots = biological replicate samples of *Cx. p. molestus* (non-biting), P (aqua) dots = biological replicate samples of *Cx. p. pipiens* (biting).

Figure S2. Transcripts that were upregulated (A) or downregulated (B) in biting *Culex pipiens pipiens* relative to non-biting *Culex pipiens molestus* RNAseq are also significantly differentially expressed in independent samples measured with qRT-PCR. Red circles = biting *Cx. pipiens pipiens*; blue squares = non-biting *Cx. pipiens molestus*. Straight lines indicate means and brackets indicate standard deviation. Significant differences in relative transcript abundance were are indicated with asterisks (Student’s t-test; * indicates p <0.05; ** indicates p < 0.01 and **** indicates p < 0.0001).

B

**
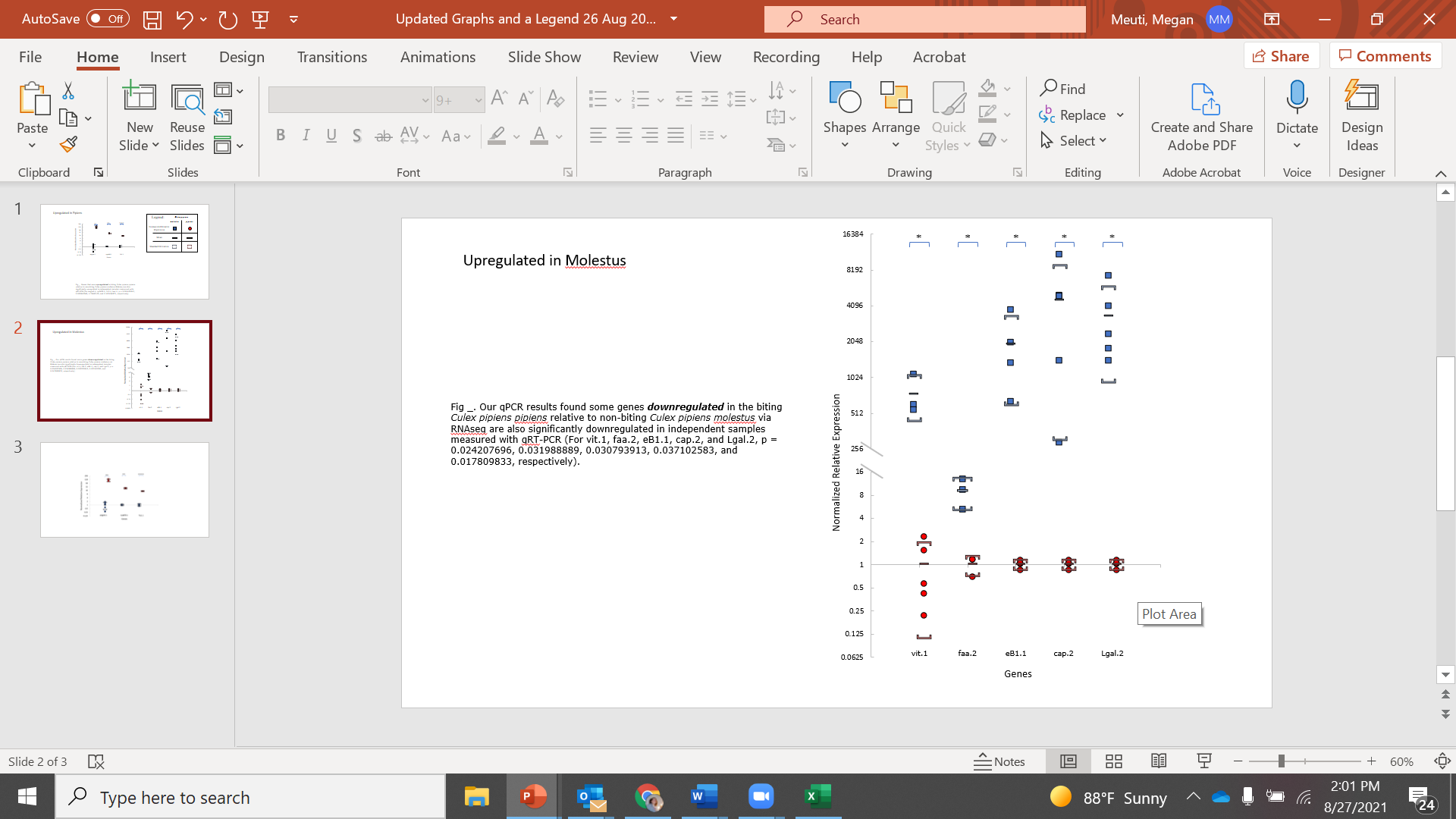

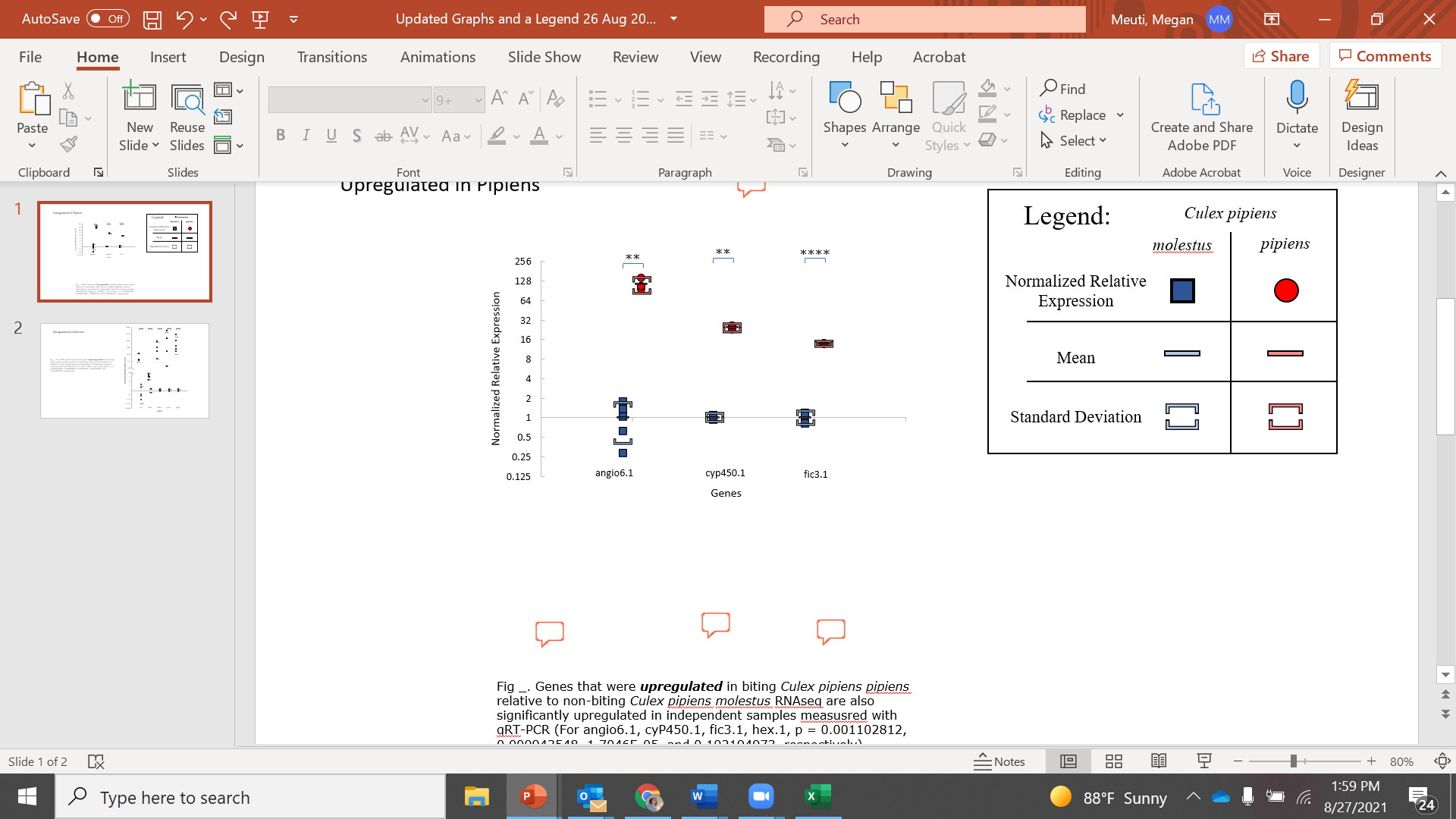
**

A

Figure S3. KEGG Ribosomal protein genes. Boxes without a color indicate genes that were not detected in the RNAseq data. Boxes in green indicate genes identified in the RNAseq data that were not differentially expressed. Boxes in red indicate genes significantly upregulated in biting Pipiens relative to non-biting Molestus. Red circles indicate genes significantly upregulated in biters relative to non-biters in both *Culex* and *Wyeomyia*.


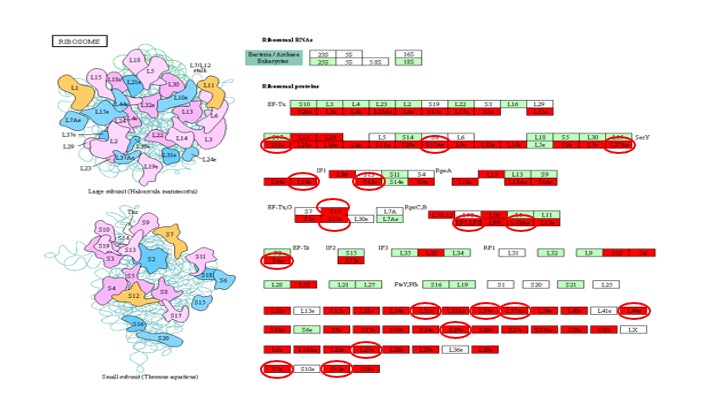


Figure S4. KEGG Oxidate phosphorylation genes. Boxes without a color indicate genes that were not detected in the RNAseq data. Boxes in green indicate genes identified in the RNAseq data that were not differentially expressed. Boxes in red indicate genes significantly upregulated in biting Pipiens relative to non-biting Molestus. None of these were genes in biters relative to non-biters in *Wyeomyia*.


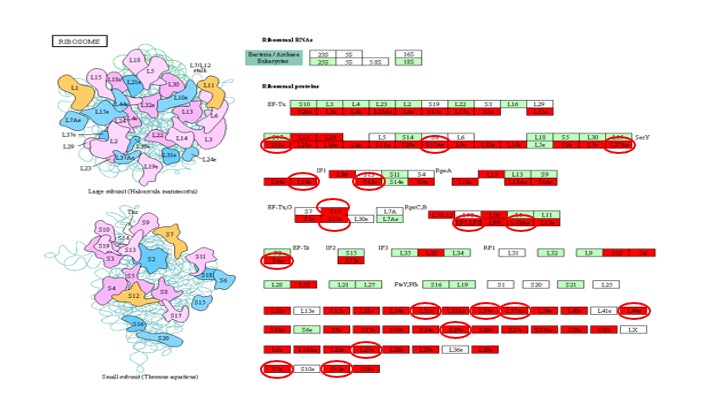

Supplement: Supplementary file 1 — Supplementary Material [file EVA-15-878-s001.docx]
